# Battle of the Bots: Solving Clinical Cases in Osteoarticular Infections With Large Language Models

**DOI:** 10.1016/j.mcpdig.2025.100230

**Published:** 2025-05-23

**Authors:** Fabio Borgonovo, Takahiro Matsuo, Francesco Petri, Seyed Mohammad Amin Alavi, Laura Chelsea Mazudie Ndjonko, Andrea Gori, Elie F. Berbari

**Affiliations:** a Division of Public Health, Infectious Diseases and Occupational Medicine, Department of Medicine, Mayo Clinic College of Medicine and Science, Mayo Clinic, Rochester, MN; b Department of Infectious Diseases, “Luigi Sacco” University Hospital, Milan, Italy; c Faculty of Medicine, Ahvaz Jundishapur University of Medical Sciences, Ahvaz, Iran; d Northwestern University, Evanston, IL; e Department of Biomedical and Clinical Sciences, University of Milan, Italy

## Abstract

**Objective:**

To evaluate the ability of 15 different large language models (LLMs) to solve clinical cases with osteoarticular infections following published guidelines.

**Materials and Methods:**

The study evaluated 15 LLMs across 5 categories of osteoarticular infections: periprosthetic joint infection, diabetic foot infection, native vertebral osteomyelitis, fracture-related infections, and septic arthritis. Models were selected systematically, including general-purpose and medical-specific systems, ensuring robust English support. In total, 126 text-based questions, developed by the authors from published guidelines and validated by experts, assessed diagnostic, management, and treatment strategies. Each model answered individually, with responses classified as correct or incorrect based on guidelines. All tests were conducted between April 17, 2025, and April 28, 2025. Results, presented as percentages of correct answers and aggregated scores, highlight performance trends. Mixed-effects logistic regression with a random question effect was used to quantify how each LLM compared in answering the study questions.

**Results:**

The performance of 15 LLMs was evaluated, with the percentage of correct answers reported. OpenEvidence and Microsoft Copilot achieved the highest score (119/126 [94.4%]), excelling in multiple categories. ChatGPT-4o and Gemini 2.5 Pro scored 117 of the 126 (92.8%). When used as references, OpenEvidence was not inferior to any comparator and was superior to 5 LLMs. Performance varied across categories, highlighting the strengths and limitations of individual models.

**Conclusion:**

OpenEvidence and Miccrosoft Copilot achieved the highest accuracy among evaluated LLMs, highlighting their potential for precisely addressing complex clinical cases. This study emphasizes the need for specialized, validated artificial intelligence tools in medical practice. Although promising, current models face limitations in real-world applications, requiring further refinement to support clinical decision making reliably.

Osteoarticular infections (OAIs) represent a growing challenge in modern health care. With advancements in medical care leading to increased global life expectancy and a surge in the use of orthopedic implants, the incidence of these OAIs continues to rise. By 2030, the annual number of total knee and hip arthroplasties is projected to exceed 4.0 million in the United States.^1^ This increase in joint replacements inevitably elevates the risk of prosthetic joint infections (PJIs), which affect up to 1% to 2% of primary arthroplasties.^2^^,^^3^

According to the latest data, hospital costs associated with hip and knee PJI are projected to reach $1.85 billion annually within the same timeframe.^4^ These conditions require prolonged antibiotic therapy, multiple surgical interventions, and extensive rehabilitation, which not only compromise patient outcomes but also place a substantial burden on health care systems,^5^^,^^6^ further exacerbated by aging and more comorbid populations.^1^

Despite the growing prevalence and complexity of OAIs, there is a global shortage of specialists trained to manage these conditions.^7^ The multifaceted nature of diagnosis, surgical management, and antimicrobial therapy often presents significant coordination challenges for orthopedic surgeons and infectious disease specialists.^7^^,^^8^ In many regions, limited access to specialized expertise further widens the gap in care quality, highlighting the urgent need for innovative solutions to support clinicians.

Generative artificial intelligence (AI), particularly large language models (LLMs), has emerged as a promising tool to address these challenges. Large language models are advanced AI models that use large data sets and transformer architectures to generate human-like text.^9^^,^^10^ They perform tasks such as text generation, information extraction, and classification with high accuracy.^11^^,^^12^ Large language models are increasingly recognized in the medical field for their potential to transform clinical practice by synthesizing large volumes of knowledge and offering guideline-based recommendations.^13^^,^^14^

A recent systematic review by Omar et al^15^ further underscored this potential, highlighting the use of natural language processing and LLMs across various infectious disease domains, including the detection of catheter-associated urinary tract infections, surveillance of pneumonia from imaging reports, and tracking of epidemiologic trends via social media and electronic health records.

Although these models have shown high accuracy in areas such as cardiology,^16^ oncology,^17^ and general infectious diseases,^18^^,^^19^ their application in OAIs remains largely underexplored.^20^ Furthermore, the limited studies available indicate that LLMs currently exhibit low accuracy when addressing OAIs-related questions.^21^^,^^22^

This study aimed to evaluate LLMs’ ability to solve OAI clinical cases in accordance with published guidelines, measuring the concordance of their responses. It also aimed to provide a ranking and comparative analysis of 15 AI software tools, offering an immediate and intuitive comparison of their overall performance. Complementing the quantitative and comparative analysis, we performed a detailed review of the 4 top-scoring models, assessing answer accuracy, reasoning rigor, and clinical validity.

## Materials and Methods

### Selection of LLMs

To identify a diverse range of LLMs, we conducted a comprehensive search using Google and PubMed to locate available chat-based systems. Through this process, we identified the following 15 distinct LLMs suitable for evaluation: Amboss (GPT), ChatGPT 4o mini, Claude-3.7 Sonnet, Consensus (GPT), DeepSeek, Gemini 2.0 Flash, Gemini 2.5 Pro, GPT-4o, MedGPT, Microsoft Copilot, OpenEvidence, Perplexity, Perplexity Pro, PubMed Buddy, and Scite.ai Plus. We included both commercial and open-access LLMs. For commercial models, we obtained subscriptions to ensure full access to their features. When a model offered both free and paid versions, we prioritized evaluating the free version first because it reflects the experience of most users. We assessed a combination of general-purpose LLMs and specialized medical LLMs to capture a broad spectrum of capabilities. The selection ensured the representation of models designed specifically for medical applications alongside more generalist systems with potential applicability in clinical contexts. Only LLMs that used English as their primary language or were multilingual with robust English support were included in the study, ensuring consistency in language interpretation and response generation across models. A comparison and detailed characteristics of each model are presented in Table 1.

**Table 1:** Characteristics of 15 LLMs

LLM name	Type of LLM	Availability	Developer	Launch year	Primary use cases	Languages supported	Multimodal capabilities	Fine-tuning support
AMBOSS GPT	Medical	Commercial	AMBOSS	2024	Medical information retrieval	English	No	No
ChatGPT 4o mini	General	Open	OpenAI	2024	Cost-efficient reasoning, coding	Multilingual	Yes	Yes
Claude 3.7 Sonnet	General	Open	Anthropic	2025	Data analytics, customer-facing agents	English	Yes	Yes
Consensus GPT	Medical	Commercial	Consensus	2023	Academic research, citation generation	English	No	No
DeepSeek	General	Open	DeepSeek	2024	Coding, mathematics, multilingual tasks	Multilingual	Yes	Yes
Gemini 2.0 Flash	General	Open	Google DeepMind	2024	Text, image, audio processing	Multilingual	Yes	Yes
Gemini 2.5 Pro	General	Commercial	Google DeepMind	2025	Complex problem solving, coding	Multilingual	Yes	Yes
GPT-4o	General	Commercial	OpenAI	2024	Text, image, audio processing	Multilingual	Yes	Yes
MedGPT	Medical	Commercial	MedGPT	2024	Health information guidance	English	No	Yes
Microsoft Copilot	General	Open	Microsoft	2023	Office automation, content generation	Multilingual	Yes	Limited
OpenEvidence	Medical	Open	OpenEvidence	2023	Medical information synthesis	English	No	No
Perplexity	General	Open	Perplexity AI	2022	Conversational search	English	No	No
Perplexity Pro	General	Commercial	Perplexity AI	2023	Enhanced search with advanced models	English	No	No
PubMed Buddy	Medical	Commercial	Samual Hatfield	2023	Literature exploration, full-text access	English	No	No
Scite.ai Plus	Medical	Commercial	Scite	2023	Citation analysis, literature review	English	No	No

AI, artificial intelligence; LLM, large language model.

### Clinical Cases and Reference Standards

This study focused on 5 major categories of orthopedic infections: (1) diabetic foot infection (DFI), (2) fracture-related infections (FRI), (3) PJI, (4) septic arthritis (SA) and (5) vertebral osteomyelitis (VO). Clinical scenarios and corresponding questions were created based on published guidelines and consensus statements to evaluate the accuracy and utility of different AI and LLMs.^23^, ^24^, ^25^, ^26^, ^27^, ^28^, ^29^, ^30^, ^31^

A complete list of the guidelines used, along with their consensus methodology and evidence grading, is provided in Supplemental Appendix 1 (available online at https://www.mcpdigitalhealth.org/). Two authors (F.B. and T.M.), developed clinical questions for each category based on realistic clinical scenarios. The questions aimed to assess diagnostic approaches, management strategies, and treatment recommendations. Subsequently, 2 additional authors (E.F.B. and F.P.) reviewed and validated these questions to ensure clinical relevance and adherence to guidelines.

Official guidelines and consensus documents were used as primary references to ensure the creation of evidence-based and clinically relevant questions for evaluating LLM performance. A deliberate decision was made to include only the strongest recommendations within each guideline, ensuring the robustness and reliability of the developed questions. A detailed process and selection are provided in Supplemental Appendix 1.

### Question Structure and Administration

We developed 126 multiple-choice questions spanning 5 infection types (20 DFI, 29 FRI, 26 PJI, 31 SA, and 20 VO) through a structured 10-step process led by 2 board-certified infectious disease specialists (Supplemental Appendix 1). Each clinical vignette incorporated 4 to 6 questions directly aligned with a single high-consensus guideline recommendation, with distractors crafted to reflect authentic clinical pitfalls. This design mandated genuine clinical reasoning by AI systems, rather than reliance on superficial textual cues.

To preserve complete model agnosticism and eliminate any advantage from multimodal or retrieval-enabled features (as described for GPT-4 Vision and Claude Sonnet 3.5), all items were delivered in plain text without images, tables, or charts.^32^ Administration was strictly sequential: we first issued the standardized board-certified ID consultant instruction (PROMPT) and then provided the clinical background and a single multiple-choice question.^33^^,^^34^ The model’s response was recorded before introducing the next question, preventing interquestion context carryover and disallowing live internet queries. All LLMs, regardless of application programming interface availability, were accessed exclusively through their web or chat interfaces under identical offline conditions. All tests were conducted between April 17, 2025, and April 28, 2025.

Each response was adjudicated as correct or incorrect against predefined, guideline-based answer keys to yield our primary accuracy metric. In addition, the 4 highest accuracy models underwent an auxiliary 6+3-point Likert-scale evaluation of explanation quality, conducted independently by 2 blinded board-certified reviewers (T.M. and S.M.A.A.) using a standardized rubric. All raw outputs, selected answers, and reviewer ratings were meticulously logged in a centralized database to ensure complete transparency and reproducibility.

### Statistical Analyses

Results were summarized as number of correct answers for each clinical question and aggregated scores for predefined categories, as detailed in Table 2. We used mixed-effects logistic regression with a random question effect to quantify how each LLM compared in answering the study questions. Pairwise odds ratios and odds ratios (ORs) between LLM were computed from the model. *P* values were adjusted by Holm method (α=.05). Results are reported as OR (95% CI, Holm-adjusted *P*) in a formatted table (Table 3) and as a log-scaled forest plot (Figure). The statistical analysis used BlueSky Statistics software, version 10.3 (BlueSky Statistics LLC). For the Likert-scale evaluation, we assessed the top 4 LLMs across all topics and reported only medians and IQRs to reflect central tendency and variability.

**Table 2:** Performance Scores of the 15 LLMs Across the 5 Clinical Topics

LLM name	DFI		FRI		PJI		SA		VO		Total score		
	Correct	Total	Correct	Total	Correct	Total	Correct	Total	Correct	Total	Correct	Total	%
Amboss (GPT)	20	20	25	29	22	26	30	31	18	20	115	126	91.27
ChatGPT 4o mini	20	20	23	29	21	26	29	31	15	20	108	126	85.71
Claude-3.7 Sonnet	18	20	25	29	21	26	30	31	17	20	111	126	88.10
Consensus (GPT)	20	20	26	29	23	26	30	31	17	20	116	126	92.06
DeepSeek	19	20	26	29	23	26	30	31	17	20	115	126	91.27
Gemini 2.0 Flash	20	20	22	29	19	26	31	31	17	20	109	126	86.51
Gemini 2.5 Pro	19	20	27	29	24	26	30	31	17	20	117	126	92.86
GPT-4o	20	20	27	29	21	26	31	31	18	20	117	126	92.86
MedGPT	20	20	25	29	22	26	30	31	17	20	114	126	90.48
Microsoft copilot	20	20	27	29	25	26	30	31	17	20	119	126	94.44
OpenEvidence	20	20	27	29	23	26	31	31	18	20	119	126	94.44
Perplexity	19	20	22	29	21	26	30	31	18	20	110	126	87.30
Perplexity Pro	18	20	22	29	23	26	31	31	17	20	111	126	88.10
PubMed Buddy	20	20	25	29	23	26	30	31	17	20	115	126	91.27
Scite.ai Plus	19	20	22	29	20	26	30	31	16	20	107	126	84.92

DFI, diabetic foot infection; FRI, fracture-related infection; LLM, large language model; PJI, prosthetic joint infection; SA, septic arthritis; VO, vertebral osteomyelitis.

**Table 3:** Mixed-Effects Logistic Regression Contrasts for Each AI Tool Versus OpenEvidence

LLMs	Odds ratio	95% CI	*P* (Holm)
Scite.ai Plus	0.077	0.009-0.658	.007
ChatGPT 4o mini	0.092	0.011-0.796	.017
Gemini 2.0 Flash	0.092	0.011-0.796	.017
Perplexity Pro	0.112	0.013-0.969	.034
Perplexity	0.112	0.013-0.969	.034
(Claude-3.7 Sonnet)	0.167	0.019-1.473	.150
MedGPT	0.259	0.029-2.340	.590
Amboss (GPT)	0.327	0.035-3.013	.998
PubMed Buddy	0.327	0.035-3.017	.998
Consensus (GPT)	0.419	0.044-3.964	>.999
DeepSeek	0.419	0.044-3.964	>.999
GPT-4o	0.546	0.056-5.343	>.999
Gemini 2.5 Pro	0.546	0.056-5.343	>.999
Microsoft Copilot	1.000	0.092-10.883	>.999

AI, artificial intelligence; LLM, large language model.

**Figure 1:**
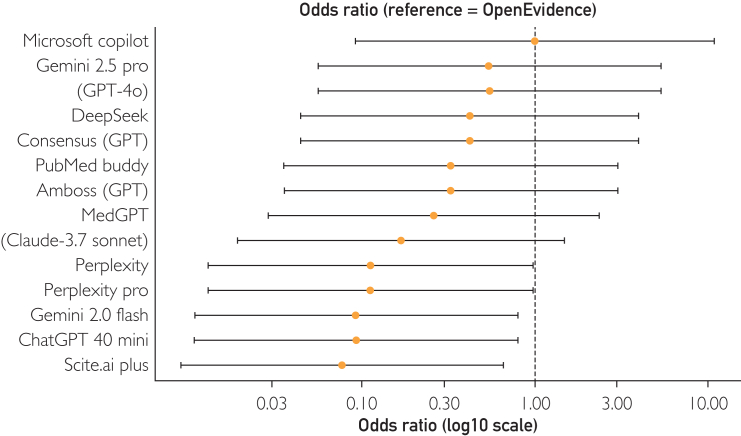
Forest plot of odds ratios for the 15 artificial intelligence tools compared with OpenEvidence.

## Results

In the overall aggregate assessment, OpenEvidence and Microsoft Copilot were the top performers, each scoring 119 of 126 (94.4%). Gemini 2.5 Pro and ChatGPT-4o followed closely with 117 of 126 (92.9%). Scite.ai Plus recorded the lowest overall performance with a score of 107 of 126 (84.9%). Table 2 presents the final scores of each LLM for every topic, along with their total scores across all categories, and Figure summarizes the correct answers for each topic.

Multiple LLMs, including Amboss (GPT), ChatGPT-4o mini, Consensus (GPT), Gemini 2.0 Flash, ChatGPT-4o, MedGPT, Microsoft Copilot, OpenEvidence, and PubMed Buddy, achieved perfect scores (20/20 [100%]) in the domain of DFI. Claude 3.7 Sonnet and Perplexity Pro had the lowest scores in this category, each answering 18 of 20 (90%) questions correctly. In the SA category, LLMs demonstrated similarly strong performance: Gemini 2.0 Flash, ChatGPT-4o, OpenEvidence, and Perplexity Pro each attained a perfect score. The lowest score in this section was 29 of 31 (93.5%), recorded by ChatGPT-4o mini.

In the PJI section, Microsoft Copilot led with 25 correct answers of 26 (96.1%), followed by Gemini 2.5 Pro with 24 (92.3%) correct responses. Gemini 2.0 Flash scored the lowest, with 19 (73%) correct answers. Notably, none of the LLMs correctly answered question 1 of clinical case 2.

For VO, ChatGPT-4o, OpenEvidence, and Perplexity each scored 18 of 20 (90%), whereas GPT-4o mini had the lowest performance (15/20 [75%]). Question 1 of clinical case 1 was missed by all models, and only Claude 3.7 Sonnet, OpenEvidence, Perplexity Pro, and Scite.ai Plus correctly answered question 3 of clinical case 4.

In the field of FRI, Gemini 2.5 Pro, ChatGPT-4o, Microsoft Copilot, and OpenEvidence achieved the highest score (27/29 [93.1%]), whereas Gemini 2.0 Flash, Perplexty, Perplexity Pro, and Scite.ai Plus had the lowest (22/29 [75.8%]). Two questions posed particular difficulty: only Gemini 2.5 Pro correctly answered question 1 of clinical case 3, whereas question 1 of clinical case 1 was correctly answered by GPT-4o mini, Gemini 2.5 Pro, and OpenEvidence. It should be noted that the performance details of all LLMs across different clinical domains are available on Mendeley Data.

Using OpenEvidence as the reference in the mixed-effects logistic model, 5 LLMs showed significantly lower odds of producing a correct answer (Figure). Scite.ai Plus (OR, 0.08; 95% CI, 0.01-0.66; *P*=.007), ChatGPT 4o mini (OR, 0.09; 95% CI, 0.01-0.80; *P*=.017), Gemini 2.0 Flash (OR, 0.09; 95% CI, 0.01-0.80; *P*=.017), Perplexity Pro (OR, 0.11; 95% CI, 0.01-0.97; *P*=.034), and Perplexity (OR, 0.11; 95% CI, 0.01-0.97; *P*=.034) after Holm adjustment (Table 3).

For the remaining 9 systems, including GPT-4o, Gemini 2.5 Pro, and Microsoft Copilot, OR estimates were not statistically different from 1 (all Holm-adjusted *P*>.05), indicating no evidence of inferiority or superiority relative to OpenEvidence within the study’s power. No LLM displayed an OR significantly >1, so the analysis supports the conclusion that OpenEvidence was not inferior to any comparator and was superior to a subset of 5 tools.

Among the 4 highest-scoring LLMs, Gemini 2.5 Pro, GPT4o, Microsoft Copilot, and OpenEvidence, Likert-scale evaluation showed uniformly high performance, with a median accuracy of 6 (IQR, 6-6; range, 1-6 for Gemini and 2-6 for others) and median completeness of 3 (IQR, 3-3; range, 1-3). Total scores were 1079/1134 (95.1%), 1095/1134 (96.5%), 1100/1134 (97%), and 1103/1134 (97.2%), respectively. Performance was consistent across most domains; however, GPT4o showed slightly lower completeness in PJI, and Gemini had broader accuracy variation in VO. On FRI, scores were high but marginally lower than in SA, suggesting slightly reduced completeness despite perfect medians. Moreover, DFI and SA had the most uniform scores.

## Discussion

To our knowledge, this is the first study to offer a descriptive and comparative ranking of 15 AI software tools, providing an immediate and intuitive comparison of their overall performance in answering OAI clinical questions across 5 key domains: DFI, FRI, PJI, SA, and VO. In this evaluation, we challenged the LLMs with 126 questions. OpenEvidence and Microsoft Copilot emerged as the top-performing models, followed closely by ChatGPT-4o and Gemini 2.5 Pro. Moreover, when used as reference, OpenEvidence was not inferior to any comparator and was superior to 5 LLMs.

Interestingly, some questions were consistently answered incorrectly by all or most LLMs. Upon reviewing current clinical guidelines, we found that these items often reflect areas of limited evidence or nuanced recommendations that are not prominently emphasized in standard summaries. For example, in question 1 of clinical case 3 (FRI), the guideline identifies smoking as a host-related risk factor for FRI, supported by 100% expert consensus but based on low-level evidence. Despite this, most of the LLMs selected more familiar comorbidities, such as diabetes, chronic kidney disease, or hypertension, instead of smoking suggesting a tendency to prioritize well-known associations over those explicitly endorsed in specialty guidelines.

Despite using widely recognized international guidelines to frame the questions, there was notable variation in the accuracy among different LLMs. This underscores the current limitations of AI systems in addressing complex clinical scenarios. Moreover, because some international recommendations rest on limited evidence, they may have introduced ambiguities that led to misinterpretation, highlighting the need for further investigation.

OpenEvidence and Microsoft Copilot demonstrated the highest performance among the evaluated models and proved to be at least not inferior to any comparator and was superior to 5 LLMs. OpenEvidence, likely due to its specialized design for medical applications. Its responses, supported by high-quality references, highlight its capability to accurately address complex clinical cases. However, this tool lacks independent validation in peer-reviewed literature, contrasting with other LLMs such as Gemini and ChatGPT. Remarkably, OpenEvidence is the first LLM to exceed 90% accuracy on the United States Medical Licensing Examination, highlighting its potential for advanced clinical problem solving.^35^

Gemini 2.5 pro and GPT-4o also achieved commendable scores. However, these 2 tools provided limited references, reducing transparency and verifiability.

The Likert-scale results among the 4 LLMs offer an interesting insight into how LLMs perform across different infectious disease topics. Areas such as DFI and SA, where guidelines are straightforward and well-established, were associated with uniformly high scores, suggesting that LLMs are reliable when recommendations are clear. In contrast, PJI and VO revealed small but consistent drops in performance, reflecting the challenges models face in domains that require more nuanced interpretation or involve variable practices. Even on FRI, where median scores were perfect, slightly lower totals point to occasional gaps in explanation depth. These findings highlight that although LLMs can handle clear-cut cases well, their reasoning may be less consistent in complex clinical scenarios, an important consideration for their safe use in practice.

Several factors likely may explain why OpenEvidence, GPT-4o, Gemini 2.5 Pro, and Microsoft Copilot led in our study (Table 1). OpenEvidence uses a retrieval-augmented framework that dynamically incorporates up-to-date biomedical literature at inference time, markedly reducing hallucinations compared with static pretraining alone.^36^ This real-time access allows it to generate responses that often reflect current evidence, functioning more as a live literature summarizer than a purely generative model. In contrast, models like GPT-4o combines hundreds of billions of parameters with a multimodal architecture, processing text, images, and audio, to enhance chain-of-thought reasoning and factual accuracy.^37^ Gemini 2.5 Pro released most recently (2025), benefits from training on fresher scientific and clinical corpora, improving its grasp of evolving guidelines and terminology. Finally, enterprise-grade assistants such as Microsoft Copilot can tap structured organizational knowledge bases, although detailed vendor disclosures are needed to confirm the exact pipelines, giving them a de facto retrieval advantage. Together, these observations indicate that integrated retrieval mechanisms and recency of training data often outweigh raw parameter count when LLMs are stressed with heterogeneous, real-world clinical scenarios.

Our study demonstrates that most LLMs are not yet fully reliable for addressing complex clinical cases involving OAIs, even when trained in medical guidelines. Models specifically tailored to the medical domain, such as OpenEvidence, show promise but require further validation. Large language models must rely on authoritative, peer-reviewed medical literature and be continuously updated to maintain relevance and accuracy.^38^

Current LLMs often depend on unfiltered and nonvalidated internet data,^39^ leading to issues such as hallucinations (fabricated or incorrect outputs). This poses significant risks when applying these models in clinical settings, especially for patient care and decision making.

Layered safeguards can mitigate this risk. Embedding LLMs within EHR-driven clinical decision support systems ensures AI suggestions are integrated into established care pathways and require explicit clinician confirmation,^40^ whereas a human-in-the-loop framework adds expert review of every recommendation before clinical use.^41^ Retrieval-augmented generation enriches responses with real-time references to guidelines and institutional protocols, enabling immediate source verification.^36^ To prevent low-confidence hallucinations, semantic entropy–based confidence scoring triggers an “I don’t know” fallback when uncertainty exceeds safe thresholds.^42^ Finally, postgeneration plausibility filters alongside secondary model audits screen outputs for dosing errors, drug–drug interactions, or fabricated facts before any recommendation reaches the clinician.^37^ These combined measures ensure AI augments rather than replace clinical judgment, preserving both patient safety and care quality.

Most existing studies evaluate LLMs in controlled environments, such as board examination–style questions.^22^^,^^43^^,^^44^ Although these tests demonstrate LLMs’ potential, their performance declines in real-world clinical scenarios.^45^, ^46^, ^47^, ^48^, ^49^ Only specialized LLMs with robust referencing capabilities may be viable for assisting clinicians in specific tasks.^39^

Moreover, even large-scale synthetic benchmarks (eg, simulations against virtual patients) can create a misleading illusion of competence that collapses without rigorous human validation.^49^ Finally, clinical practice guidelines, derived from controlled trials and expert consensus, frequently overlook the complexity and variability of everyday care, further limiting the applicability of guideline-based LLM assessments.

We acknowledge several limitations. Our clinical vignettes were based on guidelines chosen arbitrarily and may have overlooked more recent evidence. Although each multiple-choice question was rigorously derived from these recommendations, they cannot fully capture the complexity of real-world decision making. Moreover, all cases involved adult patients with OAIs, limiting applicability to pediatric populations or other infection types. We focused exclusively on the infectious disease physician perspective, whereas patient management typically involves a multidisciplinary team. Explanation quality was assessed for only the 4 highest-performing models, leaving the interpretability of the remaining LLMs unexamined. A ceiling effect emerged in our Likert-scale ratings, scores less than 4 were seldom achieved, and although consensus discussions and interrater reliability analyses bolstered consistency, our small reviewer panel may limit generalizability. We did not benchmark model outputs against expert human responses, so the clinical validity of LLM answers remains untested. Finally, because language models evolve continuously, our findings capture only a single moment in time, and the limited number of incorrect responses constrained statistical power despite application of appropriate corrections.

Despite these limitations, this study offers several notable strengths. Using realistic, clinically relevant cases crafted by infectious disease specialists, we ensured practical applicability, and by simulating typical usage, submitting identical prompts through the same workflow used by clinicians without formal AI training, we mirrored real-world conditions. Evaluating a broad spectrum of contemporary LLMs, including medical specialty and general-purpose, open-access and commercial systems, provided a comprehensive performance landscape. A large sample of questions across 5 topic areas afforded robust analysis, and the standardized, rigid methodology minimized input variability. Finally, explanation quality was assessed under blind conditions using a validated Likert-scale rubric, bolstering the objectivity and reliability of our findings.

Looking ahead, LLMs have the potential to transform clinical practice. They could assist in solving complex cases by synthesizing vast amounts of medical data to provide context-specific recommendations. Additionally, LLMs could help maintain up-to-date clinical guidelines by continuously assessing their relevance and ensuring alignment with the latest evidence.^50^ The focus on OAIs, an area with global unmet needs and a shortage of experts, underscores the potential value of LLMs to improve diagnostic and treatment quality. Further research is required to enhance the performance of LLMs in managing OAIs. Our ongoing efforts focus on real-world studies that compare AI performance with clinical guidelines and infectious disease consultants of varying expertise. By evaluating AI across a spectrum of clinical scenarios, from straightforward to complex, we aim to gain a deeper understanding of its practical applications, advantages, and limitations in routine medical practice.

## Conclusion

OpenEvidence and Microsoft Copilot achieved the highest accuracy across various OAI scenarios, with ChatGPT-4o and Gemini 2.5 Pro close behind; no model outperformed OpenEvidence, whereas 5 performed significantly worse. Large language models have the potential to assist physicians in managing OAIs, but their practical use requires a careful selection of models specifically designed for medical applications and capable of providing accurate information.

## Potential Competing Interests

The author reports no competing interests.

## Ethics Statement

This project did not involve real patient encounters or any identifiable human data. All clinical cases were constructed as questions based solely on official guidelines for osteoarticular infections. No IRB review or informed consent was required under Mayo Clinic policy, and all participating physicians contributed on a voluntary basis as part of their professional activities.
