# Supplementary material for: Battle of the Bots: Solving Clinical Cases in Osteoarticular Infections With Large Language Models
Source: Mayo Clin Proc Digit Health. 2025 May 23;3(3):100230. doi: 10.1016/j.mcpdig.2025.100230 (PMC12205795; doi:10.1016/j.mcpdig.2025.100230)
Supplement: Supplemental Table 1 [file mmc1.docx]

**Supplementary Table 1:** LIKERT scale score.

| **LLM name** | **Accuracy** | | | | | | **Completeness** | | | | | | **Total score** |
| --- | --- | --- | --- | --- | --- | --- | --- | --- | --- | --- | --- | --- | --- |
|  | **Total score** | **Median** | **Q1** | **Q3** | **IQR** | **Range** | **Total score** | **Median** | **Q1** | **Q3** | **IQR** | **Range** |  |
| **Diabetes foot infection** | | | | | | | | | | | | | |
| Gemini 2.5 Pro | 118 | 6 | 6 | 6 | 0 | 1-6 | 59 | 3 | 3 | 3 | 0 | 2-3 | 177 |
| GPT-4o | 119 | 6 | 6 | 6 | 0 | 2-6 | 59 | 3 | 3 | 3 | 0 | 2-3 | 178 |
| Microsoft copilot | 120 | 6 | 6 | 6 | 0 | 2-6 | 60 | 3 | 3 | 3 | 0 | 2-3 | 180 |
| OpenEvidence | 119 | 6 | 6 | 6 | 0 | 2-6 | 59 | 3 | 3 | 3 | 0 | 2-3 | 178 |
| **Fracture related infection (FRI)** | | | | | | | | | | | | | |
| Gemini 2.5 Pro | 161 | 6 | 6 | 6 | 0 | 1-6 | 83 | 3 | 3 | 3 | 0 | 2-3 | 244 |
| GPT-4o | 166 | 6 | 6 | 6 | 0 | 2-6 | 84 | 3 | 3 | 3 | 0 | 2-3 | 250 |
| Microsoft copilot | 168 | 6 | 6 | 6 | 0 | 2-6 | 85 | 3 | 3 | 3 | 0 | 2-3 | 253 |
| OpenEvidence | 166 | 6 | 6 | 6 | 0 | 2-6 | 84 | 3 | 3 | 3 | 0 | 2-3 | 250 |
| **Prosthetic joint infection (PJI)** | | | | | | | | | | | | | |
| Gemini 2.5 Pro | 145 | 6 | 6 | 6 | 0 | 2-6 | 72 | 3 | 3 | 3 | 0 | 2-3 | 217 |
| GPT-4o | 143 | 6 | 5.25 | 6 | 0.75 | 2-6 | 70 | 3 | 2.25 | 3 | 0.75 | 1-3 | 213 |
| Microsoft copilot | 149 | 6 | 6 | 6 | 0 | 2-6 | 74 | 3 | 3 | 3 | 0 | 2-3 | 223 |
| OpenEvidence | 146 | 6 | 6 | 6 | 0 | 2-6 | 72 | 3 | 3 | 3 | 0 | 1-3 | 218 |
| **Septic arthritis (SA)** | | | | | | | | | | | | | |
| Gemini 2.5 Pro | 181 | 6 | 6 | 6 | 0 | 1-6 | 91 | 3 | 3 | 3 | 0 | 1-3 | 272 |
| GPT-4o | 186 | 6 | 6 | 6 | 0 | 6-6 | 93 | 3 | 3 | 3 | 0 | 3-3 | 279 |
| Microsoft copilot | 182 | 6 | 6 | 6 | 0 | 2-6 | 91 | 3 | 3 | 3 | 0 | 1-3 | 273 |
| OpenEvidence | 186 | 6 | 6 | 6 | 0 | 6-6 | 93 | 3 | 3 | 3 | 0 | 3-3 | 279 |
| **Vertebral osteomyelitis (VO)** | | | | | | | | | | | | | |
| Gemini 2.5 Pro | 112 | 6 | 6 | 6 | 0 | 2-6 | 57 | 3 | 3 | 3 | 0 | 1-3 | 169 |
| GPT-4o | 116 | 6 | 6 | 6 | 0 | 3-6 | 59 | 3 | 3 | 3 | 0 | 2-3 | 175 |
| Microsoft copilot | 113 | 6 | 6 | 6 | 0 | 3-6 | 58 | 3 | 3 | 3 | 0 | 2-3 | 171 |
| OpenEvidence | 118 | 6 | 6 | 6 | 0 | 5-6 | 60 | 3 | 3 | 3 | 0 | 3-3 | 178 |
| **Total score** | | | | | | | | | | | | | |
| Gemini 2.5 Pro | 717 | 6 | 6 | 6 | 0 | 1-6 | 362 | 3 | 3 | 3 | 0 | 1-3 | 1079 |
| GPT-4o | 730 | 6 | 6 | 6 | 0 | 2-6 | 365 | 3 | 3 | 3 | 0 | 1-3 | 1095 |
| Microsoft copilot | 732 | 6 | 6 | 6 | 0 | 2-6 | 368 | 3 | 3 | 3 | 0 | 1-3 | 1100 |
| OpenEvidence | 735 | 6 | 6 | 6 | 0 | 2-6 | 368 | 3 | 3 | 3 | 0 | 1-3 | 1103 |

LLM: large language model; Q: questions
