# Supplementary material for: Battle of the Bots: Solving Clinical Cases in Osteoarticular Infections With Large Language Models
Source: Mayo Clin Proc Digit Health. 2025 May 23;3(3):100230. doi: 10.1016/j.mcpdig.2025.100230 (PMC12205795; doi:10.1016/j.mcpdig.2025.100230)
Supplement: Supplemental Appendix 1 [file mmc2.docx]

**Supplemental Appendix 1: Clinical Case Development Process**

**Summary**

1. Step 1: List of guidelines and recommendations selection
2. Step 2: PROMPT design
3. Step 3: Case Structure and Narrative Construction
4. Step 4: Question Development
5. Step 5: Distractor Design and Clinical Plausibility
6. Step 6: AI-Resistance Optimization
7. Step 7: Documentation and Referencing
8. Step 8: Question submission
9. Step 9: Response Collection and Data Management
10. Step 10: Answer Evaluation and Qualitative Assessment
11. Model-Agnostic Evaluation Pipeline

**Step 1: List of guidelines and recommendations selection**

All clinical cases and associated multiple-choice questions used to evaluate large language models (LLMs) were developed using high-quality clinical guidelines and expert consensus statements relevant to infectious diseases. For each scenario, we selected only recommendations supported by strong or clearly defined consensus methodologies. All recommendations that met these inclusion criteria were systematically collected and reviewed. When multiple guidelines addressed the same condition, they were used complementarily to enhance clinical realism and completeness. In the event of overlap, priority was given to consistency; in cases of discrepancy, we deferred to the most recent or highest-consensus recommendation. The International Consensus Meeting on Musculoskeletal Infection (ICM) and Infectious Diseases Society of America (IDSA) guidelines were prioritized. When these were unavailable or insufficient, European guidelines (such as the Septic Arthritis – Native Joint guideline (SANJO)) were incorporated. Diagnostic gaps were addressed by integrating American College of Radiology (ACR) Appropriateness Criteria, while microbiological considerations were supplemented with relevant IDSA/American Society for Microbiology (ASM) laboratory guidance.

**Diabetes-Related Foot Infections (DFI)**

Case content was derived exclusively from the 2023 International Working Group on the Diabetic Foot (IWGDF)/IDSA Guidelines on the Diagnosis and Treatment of Diabetes-related Foot Infections^1^. All recommendations included were classified using GRADE methodology and limited to *Strong*, *Conditional*, or *Best Practice* statements^2^.

**Fracture-Related Infection (FRI)**

Guideline sources included the ICM 2018 Trauma section and the 2020 international expert consensus on systemic antimicrobial therapy on FRI^3^ ^4^. The ICM recommendations were developed using a structured Delphi method, with >90% agreement (except for clinical case 1, question N3 at 85%)^5^. The antimicrobial therapy guideline followed a narrative consensus approach, with recommendations categorized as therapeutic level V in orthopedic literature^6^.

**Prosthetic Joint Infection (PJI)**

Questions integrated content from both the ICM 2018 PJI guidelines ^7^ and the 2018 IDSA/ASM guidance on microbiologic diagnostics^8^. ICM recommendations were derived via Delphi consensus, with >90% agreement^5^. The IDSA/ASM guideline, while not formally graded, provides authoritative laboratory practice guidance widely adopted in clinical microbiology.

**Septic Arthritis (SA)**

This case was informed by three independent guidelines: the SANJO guideline (GRADE-based)^9^, the 2018 IDSA/ASM diagnostic document^8^, and the ACR Appropriateness Criteria for septic arthritis^10^. SANJO statements included in the case were classified as A/B/C/D-1 or A/B-2^2^. From ACR, only recommendations rated “Usually Appropriate” (7–9) were selected. The IDSA/ASM document provided microbiologic context where applicable.

**Vertebral Osteomyelitis (VO)**

Guidance was drawn from the 2015 IDSA guidelines on native vertebral osteomyelitis ^11^, the 2018 IDSA/ASM laboratory guide^8^, and the ACR Appropriateness Criteria for suspected spine infection^12^. The 2015 IDSA guideline followed GRADE methodology; only *Strong Recommendations* were used^2^ ^13^ ^14^ ^15^. ACR criteria with ratings of “Usually Appropriate” (7–9) or “May Be Appropriate” (4–6) were incorporated as appropriate. The IDSA/ASM document provided microbiologic context where applicable providing authoritative laboratory practice guidance.

**Supplementary Table 1:** list of guidelines

| **Reference** | **Type of consensus** | **Level of consensus** |
| --- | --- | --- |
| **Diabetes foot infection (DFI)** | | |
| IWGDF/IDSA Guidelines on the Diagnosis and Treatment of Diabetes-related Foot Infections (IWGDF/IDSA 2023) | GRADE | Strong/conditional Recommendation or Best Practice Statement only |
| **Fracture related infection (FRI)** | | |
| ICM 2018 TRAUMA | Delphi method | >90% agreement (Strongest Consensus or Unanimous) except for question number 3, clinical case 1, where the consensus level is 85% |
| Recommendations for Systemic Antimicrobial Therapy in Fracture-Related Infection: A Consensus from an International Expert Group | Level of evidence in orthopedic trauma literature | Key Recommendations on Antimicrobial Therapy: Therapeutic Level V |
| **Prosthetic joint infection (PJI)** | | |
| ICM 2018 PJI | Delphi method | >90% agreement (Strongest Consensus or Unanimous) |
| A Guide to Utilization of the Microbiology Laboratory for Diagnosis of Infectious Diseases: 2018 Update by the Infectious Diseases Society of America and the American Society for Microbiology (2018 IDSA micro) | Authoritative expert guidance document | The guideline does not provide level of evidence |
| **Septic arthritis (SA)** | | |
| Guideline for management of septic arthritis in native joints (SANJO) | GRADE | A/B/C/D-1 OR A/B-2 |
| A Guide to Utilization of the Microbiology Laboratory for Diagnosis of Infectious Diseases: 2018 Update by the Infectious Diseases Society of America and the American Society for Microbiology (2018 IDSA micro): authoritative expert guidance document | Authoritative expert guidance document. | The guideline does not provide level of evidence |
| Expert Panel on Musculoskeletal Imaging et al. ACR Appropriateness Criteria® Suspected Osteomyelitis, Septic Arthritis, or Soft Tissue Infection (ACR septic arthritis) | ACR Appropriateness Criteria rating scale | Usually appropriate (7-9 points) |
| **Vertebral osteomyelitis (VO)** | | |
| 2015 Infectious Diseases Society of America (IDSA) Clinical Practice Guidelines for the Diagnosis and Treatment of Native Vertebral Osteomyelitis in Adults (2015 IDSA NVO) | GRADE | Strong recommendation (> 90%) |
| A Guide to Utilization of the Microbiology Laboratory for Diagnosis of Infectious Diseases: 2018 Update by the Infectious Diseases Society of America and the American Society for Microbiology (2018 IDSA micro) | Authoritative expert guidance document | The guideline does not provide level of evidence |
| ACR Appropriateness Criteria® Suspected Spine Infection (ACR spine infection) | ACR Appropriateness Criteria rating scale | Usually appropriate (7-9 points) and May be appropriate (4-6 points) |

**Step 2: PROMPT design**

To standardize how large language models (LLMs) engaged with each clinical case, we developed a uniform prompt that framed the interaction as a simulated infectious disease consultation. The respondent was instructed to act as a board-certified infectious disease specialist evaluating a realistic case presented by a referring clinician. The prompt clarified the expected format, reasoning style, and constraints on response length. This approach ensured consistency across cases and preserved clinical authenticity in the benchmarking process.

**Prompt:**

***You are acting as a board-certified infectious disease consultant.***
 *Your role is to evaluate clinical scenarios presented to you by a practicing physician who is exploring how large language models like you can support decision-making in infectious disease care.*

*You will be given:*

- *A clinical case (including background, patient data, and key findings)*
- *A multiple-choice question with four options (a–d), only one of which is correct*

*Your task is to:*

1. *Identify the single best answer based on the clinical scenario.*
2. *Justify your choice using expert-level clinical reasoning, as if explaining your thought process to a fellow physician.*
3. *Keep your explanation under 500 words and avoid repeating the question or answer choices.*
4. *If evidence is unclear, acknowledge uncertainty and explain your reasoning as you would in a real consultation.*

*Please format your response like this:*

*makefile*

*CopyEdit*

*Answer: [a / b / c / d]*

*Justification:*
*[Your clinical reasoning – max 500 words]*

**Step 3: Case Structure and Narrative Construction**

Following guideline selection, each clinical vignette was constructed to reflect realistic scenarios encountered in osteoarticular infection (OAI) practice. Cases were authored by two infectious disease specialists to replicate the clinical complexity and ambiguity typical of real-world consultations while maintaining strict adherence to the guideline recommendations outlined in Step 1.

Each case followed a standardized structure:

- A focused clinical narrative including the presenting complaint, relevant medical history, comorbidities, physical exam findings, and diagnostic data (labs, imaging, microbiology).
- Disease progression over time to simulate evolving decision-making.
- Multiple-choice questions (MCQs), each targeting a specific high-consensus guideline recommendation.

MCQs were developed to test applied clinical reasoning rather than factual recall. Each correct answer corresponded to a clearly defined recommendation in the guidelines. When multiple guidelines addressed the same issue, the most recent or highest-consensus recommendation was prioritized. Items lacking a definitive guideline anchor were excluded.

Distractors were based on common clinical pitfalls or outdated practices and were reviewed for plausibility. To minimize superficial pattern recognition by AI systems, narrative and question phrasing resembled authentic consultant-level communication, avoiding stylized or exam-style wording. Questions were embedded directly into the narrative and not presented in isolation.

Each case was iteratively refined to ensure clarity, internal consistency, and faithful representation of source recommendations. The full set of references and recommendation levels is detailed in the supplementary material and methods 3.

**Step 4: Question Development**

Each case included from 4 to 6 multiple-choice questions (MCQs), each mapped to a specific, high-consensus recommendation from the selected guidelines (see Supplementary Material and Methods 2). Only recommendations with a clearly defined level of agreement—such as GRADE strong/conditional,>90% Delphi consensus, or formal best practice statements—were eligible.

Questions were structured to test clinical decision-making, not factual recall. Each question included four answer options, with only one correct answer, explicitly supported by the cited guideline. Distractors were evidence-based and designed to reflect realistic but incorrect clinical choices.

Answers were considered correct only if they aligned exactly with the selected recommendation from the source guideline. No partial credit or interpretive scoring was applied.

In cases where multiple guidelines addressed the same issue, we selected the most recent or highest-consensus source to define the correct answer. Questions without a single, clearly supported recommendation were excluded to avoid interpretive scoring.

All questions were written in natural clinical language, avoiding academic or test-like phrasing, to mirror real-world medical communication and reduce pattern recognition by LLMs.

**Step 5: Distractor Design and Clinical Plausibility**

Distractors were crafted to reflect common diagnostic errors, outdated practices, or plausible, but incorrect, clinical decisions. Each distractor was carefully designed to avoid obvious elimination based on keyword recognition, thereby requiring models (and clinicians) to apply context-specific reasoning.

**Step 6: AI-Resistance Optimization**

To minimize the influence of memorized training data or superficial pattern recognition, questions were embedded within clinical narratives rather than presented as isolated items. No standardized or USMLE-style formatting was used. Each question was iteratively refined to eliminate lexical cues or overly formulaic structure.

**Step 7: Documentation and Referencing**

Each question was mapped to a specific recommendation, with citation format standardized as: 📚 *[Guideline name, section number, question number]*. The complete list of guidelines, consensus methods, and evidence levels is provided in the supplementary material.

**Step 8: Question Submission**

Each clinical case was submitted manually to each large language model (LLM) in a standardized and controlled format to ensure consistency and prevent bias from prior context. For every LLM, we followed the same structured procedure.

Once an LLM was selected to answer a case, we proceeded as follows:

1. **We first pasted the prompt** (instructing the model to act as a board-certified infectious disease consultant).
2. **We then submitted the clinical background** and **only the first question (Question 1)**.
3. After the LLM generated its answer, we submitted **the next question (Question 2)**.
4. This process was repeated **sequentially, one question at a time**, until all questions for that case were completed.

At no point was the full case, including all questions, provided at once. This design minimized the risk of response contamination, where earlier answers could influence the correctness of later ones due to contextual inference.

After all questions for a clinical case were completed, we moved on to the next case and repeated the same step-by-step process.

The only exception to this process was **OpenEvidence**. This model does not allow pasting of custom prompts. For OpenEvidence, we submitted only the clinical background and questions, without the standardized consultant prompt used for other models.

**Step 9: Response Collection and Data Management**

All responses generated by the LLMs were recorded in their original, unedited format. These raw outputs are available in **Supplementary Material and Methods 4**, providing full transparency and allowing external review of each model’s reasoning and language.

In parallel, all answers were systematically extracted and compiled into a structured Excel file. Each row corresponds to one LLM’s response to a specific question, including metadata such as model ID, case number, question index, and selected answer. This format allowed for efficient scoring, comparison, and further analysis.

**Step 10: Answer Evaluation and Qualitative Assessment**

After completing all case submissions, we exported every selected answer into an Excel spreadsheet—the most straightforward way to organize and review our quantitative data. Because each MCQ had only one correct option (A–D), we recorded the letter chosen by each model and scored it as **Correct** or **Incorrect** against the predefined guideline-based answer.

For qualitative insights, we then focused on the five LLMs with the highest accuracy. Two board-certified infectious disease specialists independently evaluated each model’s free-text justifications for the first three questions of every case using a dual-domain Likert scoring protocol:

- **Accuracy** (6-point scale)
- **Completeness** (3-point scale)

The full scoring descriptors and procedures are detailed in the Expert-Based Scoring Protocol (see below). Any scoring discrepancies greater than one point were resolved by consensus discussion, and interrater reliability was calculated to ensure consistent application of the scale.

Expert-Based Scoring Protocol for LLM Responses

Purpose
To systematically evaluate the quality of answers generated by large language models (LLMs) in response to structured clinical questions regarding osteoarticular infections, using a validated dual-domain scoring system based on accuracy and completeness.

# **Scoring Overview**

Each answer will be assessed independently by two infectious disease experts according to two distinct dimensions:

1. Accuracy – degree of factual and clinical correctness.
2. Completeness – degree to which the answer addresses all relevant aspects of the question.

## **1. Accuracy Scoring (6-point Likert Scale)**

| Score | Description |
| --- | --- |
| 6 | Completely correct: Fully concordant with guidelines, no factual or clinical errors. Safe and appropriate. |
| 5 | Nearly all correct: Minor omissions or vague phrasing, but overall accurate and safe. |
| 4 | More correct than incorrect: Some factual issues, but directionally appropriate and not clinically dangerous. |
| 3 | Mixed: Equal parts correct and incorrect; ambiguous or misleading. Clinical safety unclear. |
| 2 | More incorrect than correct: Substantial inaccuracies, partially misleading, not aligned with clinical standards. |
| 1 | Completely incorrect: Factually wrong, clinically unsafe, or guideline-discordant. |

Note: If an answer is scored as 1 for accuracy, the completeness score should not be assigned.

## **2. Completeness Scoring (3-point Likert Scale)**

| Score | Description |
| --- | --- |
| 3 | Comprehensive: All key components of the clinical question are addressed with contextual or nuanced details. |
| 2 | Adequate: All major aspects are covered, though the answer may lack depth or precision. |
| 1 | Incomplete: Key components of the question are omitted or insufficiently addressed. |

# **Scoring Procedure**

• Each expert independently reviews the LLM-generated answer and assigns an accuracy and completeness score.
• Discrepancies >1 point will be discussed jointly to reach consensus.
• Interrater reliability will be calculated using Cohen’s Kappa (for categorical agreement) or ICC (for ordinal agreement), as appropriate.

# **Data Handling**

• Scores will be recorded in a shared spreadsheet with LLM identifier, question ID, and scores per reviewer.
• Mean and standard deviation or IQR for accuracy and completeness will be calculated per model and per question category (diagnosis, treatment, management).

# **Use of Results**

This scoring system supports a nuanced evaluation of LLM output quality in clinical infectious disease decision-making, helping benchmark model performance and identify opportunities for improvement in guideline-based reasoning.

# **References**

<https://jamanetwork.com/journals/jamanetworkopen/fullarticle/2809975>

**Model-Agnostic Evaluation Pipeline**

To eliminate any discrepancies arising from differences in model interfaces or capabilities, and thereby ensure that performance comparisons reflect true differences in reasoning rather than variations in how inputs are delivered, we implemented a fully standardized, model-agnostic workflow as follows:

**Prompt Standardization:**

We crafted a single, comprehensive instruction framing each task as a simulated consultation with a board-certified infectious disease specialist. This instruction was supplied verbatim to every LLM that permits custom prompt injection. In the one exception, OpenEvidence, which does not allow user-defined prompts, we nonetheless preserved the same structure by submitting only the clinical vignette and question text, without altering content or format. This approach guaranteed that all models received identical guidance on expected format, reasoning style, and response constraints.

**Uniform Clinical Vignettes:**

All evaluated LLMs were presented with precisely the same text-only clinical scenarios. We deliberately omitted any images, tables, charts, or other non-textual elements so that model performance could not benefit from specialized multimodal capabilities. Each vignette was written in consistent, consultant-level prose to reflect realistic clinical narratives and to prevent any model from gaining an edge through formatting cues.

**Sequential Submission Workflow:**

To prevent information carryover between questions, we adopted a one-by-one submission method. After issuing the standard prompt, we provided the model with the case background plus only the first multiple-choice question. Once the response was recorded in full, we introduced the next question in isolation. By never revealing subsequent questions or summary data, this procedure assured that each answer was generated solely on the basis of that question’s text and the model’s internal knowledge.

**Interface Consistency:**

Rather than mixing programmatic API calls with manual text entry, we accessed every LLM exclusively through its standard web or chat interface. We did not embed any retrieval or plugin instructions, URLs, or live-search commands in our prompts. Although some models offer APIs, we intentionally refrained from API use to maintain identical input methods across all systems. This “web-only” strategy ensured that no model could leverage external tool integrations or real-time internet searches.

**Controlled Knowledge Environment:**

All testing was conducted without providing the models with any external documents, hyperlinks, or search directives. While models inherently vary in their access to pre-indexed knowledge, we deliberately did not facilitate any live lookups or retrieval extensions. Consequently, each LLM was forced to rely exclusively on its pretrained internal knowledge base and the text we supplied, eliminating disparities introduced by dynamic internet queries.

**Unified Evaluation Metrics:**

We applied the same scoring rubric to every response. Each answer choice (A–D) was judged “correct” or “incorrect” against our a priori, guideline-derived answer keys to generate an objective accuracy metric. For the five models with the highest raw accuracy, we further conducted a five-point Likert-scale assessment of explanation depth and clinical reasoning quality, using a standardized rubric completed independently by two board-certified reviewers. All raw outputs, selected answers, and reviewer scores were logged verbatim in a centralized database, ensuring full transparency and enabling exact reproduction of our analyses.

All steps in the case construction, question delivery, and response management were documented in detail to support full reproducibility. All source guidelines, raw model outputs, and scoring frameworks are available in the supplementary material.

**References:**

1. É S, Z A, Sa van A, et al. IWGDF/IDSA guidelines on the diagnosis and treatment of diabetes-related foot infections (IWGDF/IDSA 2023). *Diabetes Metab Res Rev*. 2024;40(3). doi:10.1002/dmrr.3687

2. Guyatt GH, Oxman AD, Vist GE, et al. GRADE: an emerging consensus on rating quality of evidence and strength of recommendations. *BMJ*. 2008;336(7650):924-926. doi:10.1136/bmj.39489.470347.AD

3. Trauma. ICM Philly. September 18, 2018. Accessed December 29, 2024. https://icmphilly.com/document/icm-2018-trauma-document/

4. Recommendations for Systemic Antimicrobial Therapy in Fracture-Related Infection: A Consensus From an International Expert Group - PubMed. Accessed December 29, 2024. https://pubmed.ncbi.nlm.nih.gov/31567902/

5. Nasa P, Jain R, Juneja D. Delphi methodology in healthcare research: How to decide its appropriateness. *World J Methodol*. 2021;11(4):116-129. doi:10.5662/wjm.v11.i4.116

6. Luksameearunothai K, Chaudhry Y, Thamyongkit S, Jia X, Hasenboehler EA. Assessing the level of evidence in the orthopaedic literature, 2013–2018: a review of 3449 articles in leading orthopaedic journals. *Patient Saf Surg*. 2020;14(1):23. doi:10.1186/s13037-020-00246-6

7. Hip and Knee. ICM Philly. September 24, 2018. Accessed December 29, 2024. https://icmphilly.com/document/icm-2018-hip-and-knee-document/

8. Miller JM, Binnicker MJ, Campbell S, et al. A Guide to Utilization of the Microbiology Laboratory for Diagnosis of Infectious Diseases: 2018 Update by the Infectious Diseases Society of America and the American Society for Microbiologya. *Clin Infect Dis*. 2018;67(6):e1-e94. doi:10.1093/cid/ciy381

9. Ravn C, Neyt J, Benito N, et al. Guideline for management of septic arthritis in native joints (SANJO). *J Bone Jt Infect*. 2023;8(1):29-37. doi:10.5194/jbji-8-29-2023

10. Expert Panel on Musculoskeletal Imaging, Pierce JL, Perry MT, et al. ACR Appropriateness Criteria® Suspected Osteomyelitis, Septic Arthritis, or Soft Tissue Infection (Excluding Spine and Diabetic Foot): 2022 Update. *J Am Coll Radiol JACR*. 2022;19(11S):S473-S487. doi:10.1016/j.jacr.2022.09.013

11. Berbari EF, Kanj SS, Kowalski TJ, et al. 2015 Infectious Diseases Society of America (IDSA) Clinical Practice Guidelines for the Diagnosis and Treatment of Native Vertebral Osteomyelitis in Adults. *Clin Infect Dis Off Publ Infect Dis Soc Am*. 2015;61(6):e26-46. doi:10.1093/cid/civ482

12. Expert Panel on Neurological Imaging, Ortiz AO, Levitt A, et al. ACR Appropriateness Criteria® Suspected Spine Infection. *J Am Coll Radiol JACR*. 2021;18(11S):S488-S501. doi:10.1016/j.jacr.2021.09.001

13. Guyatt GH, Oxman AD, Kunz R, et al. Going from evidence to recommendations. *BMJ*. 2008;336(7652):1049-1051. doi:10.1136/bmj.39493.646875.AE

14. Schünemann HJ, Oxman AD, Brozek J, et al. Grading quality of evidence and strength of recommendations for diagnostic tests and strategies. *BMJ*. 2008;336(7653):1106-1110. doi:10.1136/bmj.39500.677199.AE

15. Brożek JL, Akl EA, Compalati E, et al. Grading quality of evidence and strength of recommendations in clinical practice guidelines part 3 of 3. The GRADE approach to developing recommendations. *Allergy*. 2011;66(5):588-595. doi:10.1111/j.1398-9995.2010.02530.x
