# Supplementary material for: Battle of the Bots: Solving Clinical Cases in Osteoarticular Infections With Large Language Models
Source: Mayo Clin Proc Digit Health. 2025 May 23;3(3):100230. doi: 10.1016/j.mcpdig.2025.100230 (PMC12205795; doi:10.1016/j.mcpdig.2025.100230)

**List of recommendations**

**DIABETIC FOOT INFECTION (DFI)**

# **Guideline:** IWGDF/IDSA Guidelines on the Diagnosis and Treatment of Diabetes-related Foot Infections (IWGDF/IDSA 2023)

### Recommendation 1

Diagnose a soft tissue diabetes-related infection clinically based on the presence of local or systemic signs and symptoms of inflammation. (Grading of Recommendations, Assessment, Development, and Evaluation (GRADE) recommendation: Strong; Certainty of evidence: Low)

Asses the severity of any Diabetes-related foot infection (DFI) using the International Working Group on the Diabetic Foot (IWGDF)/Infectious Diseases Society of America (IDSA) classification scheme. (Strong; Low).


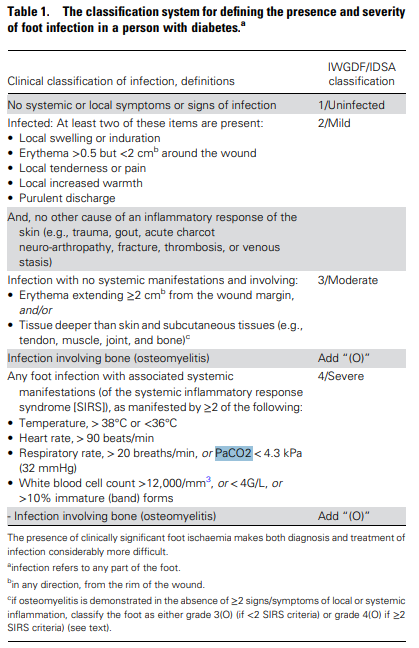


- ***Question in the text:*** *clinical case 1, question 1*
- ***Question in the text:*** *clinical case 2, question 2*
- ***Question in the text:*** *clinical case 4, question 1*
- ***Question in the text:*** *clinical case 4, question 2*

### Recommendation 3

Assess inflammatory serum biomarkers such as C-reactive protein (CRP), erythrocyte sedimentation rate (ESR), or procalcitonin (PCT) in a person with diabetes and a possible infected foot ulcer for whom the clinical examination is diagnostically equivocal or uninterpretable. (Best Practice Statement).


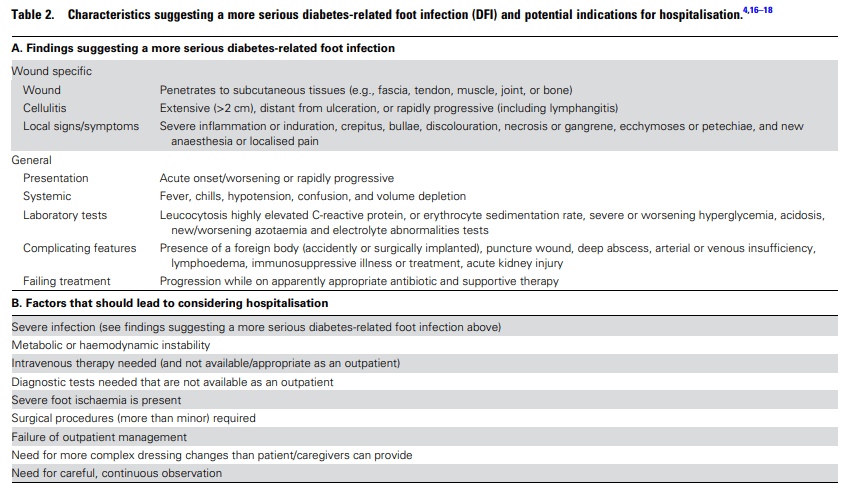


- ***Question in the text:*** *Clinical case 2, question 1*
- ***Question in the text:*** *Clinical case 2, question 3*
- ***Question in the text:*** *Clinical case 3, question 2*

### Recommendation 5

In a person with suspected soft tissue DFI, consider a sample for culture to determine the causative microorganisms, preferably by aseptically collecting a tissue specimen (by curettage or biopsy) from the wound. (Conditional; Moderate).

- ***Question in the text:*** *clinical case 4, question 3*

### Recommendation 6

Use conventional, rather than molecular, microbiology techniques for the first-line identification of pathogens from soft tissue or bone samples in a patient with a DFI. (Strong; Moderate).

- ***Question in the text:*** *Clinical case 1, question 3*

### Recommendation 8

Perform magnetic resonance imaging (MRI) when the diagnosis of diabetes-related osteomyelitis of the foot remains in doubt despite clinical, plain X-rays and laboratory findings. (Strong; Moderate).

- ***Question in the text:*** *Clinical case 1, question 5*

### Recommendation 10

In a person with diabetes for whom there is a suspicion of osteomyelitis of the foot (before or after treatment), bone (rather than soft tissue) samples should be obtained for culture, either intraoperatively or percutaneously. (Conditional; Moderate).

- ***Question in the text:*** *Clinical case 2, question 2*

### Recommendation 11

Do not treat clinically uninfected foot ulcers with systemic or local antibiotic therapy when the goal is to reduce the risk of new infection or to promote ulcer healing. Best Practice Statement.

### Recommendation 12

(a) Use any of the systemic antibiotic regimens that have been shown to be effective in published randomized controlled trials at standard (usual) dosing to treat a person with diabetes and a soft tissue infection of the foot. (Strong; High).

(b) Administer antibiotic therapy to a patient with a skin or soft tissue diabetic foot infection for a duration of 1–2 weeks. (Strong; High).

(c) If evidence of infection has not resolved after 4 weeks of apparently appropriate therapy, re-evaluate the patient, and reconsider the need for further diagnostic studies or alternative treatments. (Strong; Low).

- ***Question in the text:*** *Clinical case 2, question 4*
- ***Question in the text:*** *Clinical case 2, question 5*
- ***Question in the text:*** *Clinical case 4, question 4*

### Recommendation 13

Select an antibiotic agent for treating a DFI based on the likely or proven causative pathogen(s) and their antibiotic susceptibilities; the clinical severity of the infection; published evidence of the efficacy of the agent for infections of the diabetes-related foot; the risk of adverse events including collateral damage to the commensal flora; the likelihood of drug interactions; agent availability and costs. Best Practice Statement.

- ***Question in the text:*** *Clinical case 4, question 5*

### Recommendation 14

Target aerobic gram-positive pathogens only (beta- haemolytic streptococci and Staphylococcus aureus including methicillin-resistant strains if indicated) for people with a mild DFI, who have not recently received antibiotic therapy, and who reside in North America or Western Europe. Best Practice Statement.

### Recommendation 15

Do not empirically target antibiotic therapy against Pseudomonas aeruginosa in cases of DFI in temperate climates, but use empirical treatment of P. aeruginosa if it has been isolated from cultures of the affected site within the previous few weeks, in a person with moderate or severe infection who resides in Asia or North Africa. Best Practice Statement.

- ***Question in the text:*** *Clinical case 3, question 3*

### Recommendation 17

Use the outcome at a minimum follow-up duration of 6 months after the end of the antibiotic therapy to diagnose remission of diabetes-related osteomyelitis of the foot. Best Practice Statement.

- ***Question in the text:*** *Clinical case 3, question 5*

### Recommendation 18

The urgent surgical consultation should be obtained in cases of severe infection or moderate DFI complicated by extensive gangrene, necrotising infection, signs suggesting deep (below the fascia) abscess, compartment syndrome, or severe lower limb ischaemia. Best Practice Recommendation.

- ***Question in the text:*** *Clinical case 3, question 1*

### Recommendation 20

In people with diabetes, PAD and a foot ulcer or gangrene with infection involving any portion of the foot obtain an urgent consultation by a surgical specialist as well as a vascular specialist in order to determine the indications and timings of a drainage and/or revascularisation procedure. Best Practice Statement.

- ***Question in the text:*** *Clinical case 3, question 4*

**FRACTURE RELATED INFECTION (FRI)**

# **Guideline:** ICM 2018 Trauma

## 1.Prevention

### 1.1. PREVENTION: HOST FACTORS

#### *QUESTION 1:*

What is the relationship between smoking and infection in fracture procedures? Is smoking history or only current smoking important? Does nicotine cessation at time of fracture reduce complication rates?

RECOMMENDATION: Smoking seems to increase the risk of infection in fracture procedures. The importance of smoking history versus current smoking status is unknown. It is also unknown if nicotine cessation (smoking) at time of fracture treatment reduces complication rates.

LEVEL OF EVIDENCE: Limited

DELEGATE VOTE: Agree: 100%, Disagree: 0%, Abstain: 0% (Unanimous, Strongest Consensus)

- **Question in the text:** clinical case 3, question 1

#### *QUESTION 2:*

What is the role of nutritional supplementation (NS) in avoiding infection in acute fracture cases?

RECOMMENDATION: (1) Evidence does not support the role of NS for avoiding infections in well-nourished individuals. (2) However, the literature has stated that in patients with a nutritional deficiency or catabolic state restoring nutritional parameters might reduce the risk of infection.

LEVEL OF EVIDENCE: (1) Limited, (2) Moderate

DELEGATE VOTE: Agree: 100%, Disagree: 0%, Abstain: 0% (Unanimous, Strongest Consensus)

- **Question in the text:** clinical case 6, question 1

*QUESTION 3:*

Do preoperative pneumonia/urinary tract infections (UTIs)/trophic ulcers increase periprosthetic joint infection/surgical site infection (PJI/SSI) risk in femoral neck fracture patients treated by partial/total hip arthroplasty (THA)?

RECOMMENDATION: There is a paucity of literature examining whether pneumonia/UTI/trophic ulcers increase SSI/PJI risk for patients with femoral neck fracture treated by hemi- or THA.

LEVEL OF EVIDENCE: Limited

DELEGATE VOTE: Agree: 91%, Disagree: 0%, Abstain: 9% (Super Majority, Strong Consensus)

- **Question in the text:** clinical case 6, question 2

#### *QUESTION 4:*

Are there microorganism-specific risk factors for acute infection in trauma patients (i.e., does being a nasal carrier of methicillin-resistant Staphylococcus aureus (S. aureus), or MRSA, increase the risk for MRSA infection after trauma?

RECOMMENDATION: The current evidence of an increased risk of infection is based on several risk factors, including MRSA colonization, presence of external fixator, anatomical location of surgery and severe open fractures. In these situations, alterations in antibiotic prophylaxis could be considered.

LEVEL OF EVIDENCE: Moderate

DELEGATE VOTE: Agree: 100%, Disagree: 0%, Abstain: 0% (Unanimous, Strongest Consensus)

- **Question in the text:** clinical case 6, question 3

#### *QUESTION 5:*

Is periprosthetic fracture a risk for the development of a periprosthetic joint infection (PJI)?

RECOMMENDATION: Infection rates from level III and IV evidence studies suggest an increased surgical site infection in patients who undergo re-operation for treatment of periprosthetic fracture of the femur after total hip and knee arthroplasty. There is limited literature available on periprosthetic acetabular and tibial fractures. Further study investigating the outcomes for treatment of periprosthetic fracture is recommended.

LEVEL OF EVIDENCE: Limited

DELEGATE VOTE: Agree: 100%, Disagree: 0%, Abstain: 0% (Unanimous, Strongest Consensus)

#### *QUESTION 6:*

Are there predictors of the need for allogeneic blood transfusion (ABT) in patients undergoing arthroplasty for acute hip fractures?

RECOMMENDATION: Preoperative predictors for the need for ABT include (1) anemia and (2) dementia and hypoalbuminemia. (3) Anticoagulation or anti-platelet medications do not predict the need for ABT. There is conflicting data with regard to the need for ABT when comparing hemiarthroplasty (HA) to total hip arthroplasty (THA).

LEVEL OF EVIDENCE: (1) Strong, (2) Limited, (3) Moderate

DELEGATE VOTE: Agree: 100%, Disagree: 0%, Abstain: 0% (Unanimous, Strongest Consensus)

- **Question in the text:** clinical case 5, question 1

### 1.2. PREVENTION: RISK MITIGATION

#### *QUESTION 1:*

Is there a role for bacterial decolonization (i.e., of methicillin-resistant Staphylococcus aureus (S. aureus), or MRSA, in nares) in trauma cases?

RECOMMENDATION: It is unknown if bacterial decolonization in trauma patients reduces surgical site infection (SSI).

LEVEL OF EVIDENCE: Limited

DELEGATE VOTE: Agree: 100%, Disagree: 0%, Abstain: 0% (Unanimous, Strongest Consensus)

- Question in the text: clinical case 4, question 2

#### *QUESTION 2:*

What are the ideal strategies to prevent secondary and nosocomial contamination of open fracture wounds which are left open?

RECOMMENDATION: Data support local antibiotics and early wound closure to reduce contamination of open facture wounds.

NOTE: The recommendation above was changed from the original version so the rationale below does not completely align with this recommendation. Please see Section 3:2, Question 2 for rationale for early wound closure. The rationale below regarding negative pressure wound therapy (NPWT) applies to Section 3:2, Question 4.

LEVEL OF EVIDENCE: Moderate

DELEGATE VOTE: Agree: 100%, Disagree: 0%, Abstain: 0% (Unanimous, Strongest Consensus)

#### *QUESTION 3:*

Is there a difference in the risk of periprosthetic joint infection (PJI) with use of internal versus external fixation for treatment of periprosthetic fractures?

RECOMMENDATION: Unknown. There is limited evidence comparing the risk of PJI with use of internal versus external fixation to treat periprosthetic fracture. The potential for pin tract infection, particularly with inadvertently placed intra-articular pins, make internal fixation the preferable treatment option in most cases.

LEVEL OF EVIDENCE: Limited

DELEGATE VOTE: Agree: 90%, Disagree: 5%, Abstain: 5% (Super Majority, Strong Consensus)

- **Question in the text:** clinical case 6, question 4

#### *QUESTION 4:*

Should definitive fixation of fracture in a polytrauma patient and open abdomen be delayed until the abdomen is closed?

RECOMMENDATION: Definitive fracture fixation in the presence of an open abdomen should not be delayed and could be performed safely if the patient is suitable to undergo surgery.

LEVEL OF EVIDENCE: Limited

DELEGATE VOTE: Agree: 95%, Disagree: 5%, Abstain: 0% (Unanimous, Strongest Consensus)

- **Question in the text:** clinical case 4, question 1

## 2.Diagnosis

#### *QUESTION 1:*

Which open fracture classification system currently used (Gustilo-Anderson classification or the Orthopaedic Trauma Association’s open fracture classification (OTA-OFC)) is preferred, based on interobserver reproducibility and predictiveness of outcomes?

RECOMMENDATION: OTA-OFC is preferred. Based on currently-available data, the OTA-OFC provides a more robust description of the injury with interobserver agreement that is comparable or superior to the Gustilo-Anderson classification. Additionally, the OTA-OFC, according to its subcategories, may predict outcomes such as the likelihood of early amputation and need for adjuvant treatments.

LEVEL OF EVIDENCE: Limited

DELEGATE VOTE: Agree: 95%, Disagree: 0%, Abstain: 5% (Unanimous, Strongest Consensus)

- **Question in the text:** clinical case 5, question 4

#### *QUESTION 2:*

What diagnostic criteria must be fulfilled to diagnose surgical site infection (SSI) or fracture related infection (FRI) in orthopaedic trauma (including external fi xators)?

RECOMMENDATION: Diagnostic criteria proposed by the International Consensus Group on FRI (published in 2017) should be used to diagnose infection in fracture cases. In cases, more than four weeks from fracture, histological confi rmation of > 5 neutrophils per high power fi eld is confi rmatory of infection.

LEVEL OF EVIDENCE: Consensus

DELEGATE VOTE: Agree: 85%, Disagree: 5%, Abstain: 10% (Super Majority, Strong Consensus)

- **Question in the text:** Clinical case 1, question 3

#### *QUESTION 4:*

What differentiates acute from chronic osteomyelitis (OM)? Is it clinically important to distinguish one from the other?

RECOMMENDATION: Current literature is lacking consistent criteria for a distinct time point that differentiates the acute and chronic forms of infection. Differentiating between acute and chronic types may have practical implications on treatment plan and final prognosis.

LEVEL OF EVIDENCE: Limited

DELEGATE VOTE: Agree: 95%, Disagree: 5%, Abstain: 0% (Unanimous, Strongest Consensus)

- **Question in the text:** clinical case 3, question 4

#### *QUESTION 5:*

Is synovial fluid or fracture hematoma always aseptic? If not, could this play a role in acute infection or periprosthetic joint infection (PJI) after open reduction and internal fixation (ORIF)?

RECOMMENDATION: Fracture hematoma is not always aseptic. It is unknown if synovial fluid is always aseptic. In addition, it is unclear if this plays a role in acute infection or fracture-related infection (FRI) after ORIF.

LEVEL OF EVIDENCE: Moderate

DELEGATE VOTE: Agree: 95%, Disagree: 5%, Abstain: 0% (Unanimous, Strongest Consensus)

- **Question in the text:** clinical case 5, question 2

#### *QUESTION 6:*

What is the relationship between implanted metal and colonization under a vacuum-assisted closure (VAC) in open fractures?

RECOMMENDATION: The use of negative pressure wound therapy (NPWT or VAC) over exposed orthopaedic implants has been reported but its role remains unknown. Furthermore, no evidence exists regarding the effect of NPWT on the colonization of metal implants in open fractures. Further research is required to provide more insight into this question.

LEVEL OF EVIDENCE: Consensus

DELEGATE VOTE: Agree: 100%, Disagree: 0%, Abstain: 0% (Unanimous, Strongest Consensus)

- **Question in the text:** clinical case 5, question 3

## 3.Treatment

### 3.1. TREATMENT: ANTIBIOTICS AND NONOPERATIVE MANAGEMENT

#### *QUESTION 1:*

What is the most optimal prophylaxtic antibiotic coverage and treatment duration for open fractures of long bones?

RECOMMENDATION: The use of prophylactic antibiotics for open fractures of long bones has a protective effect against early infection. Antibiotics should be administered as soon as possible after the injury. The antibiotic of choice should target gram-positive organisms. Additional coverage for gram-negative organisms should be considered for patients with high-energy open fractures. Antibiotics should not be continued for more than 72 hours after wound closure.

LEVEL OF EVIDENCE:

• Efficacy of prophylactic antibiotics – Strong

• Timing of prophylactic antibiotics – Moderate

• Choice of antibiotics – Limited

• Treatment duration – Moderate

DELEGATE VOTE: Agree: 100%, Disagree: 0%, Abstain: 0% (Unanimous, Strongest Consensus)

- **Question in the text:** Clinical case 1, question 1
- **Question in the text:** Clinical case 1, question 2
- **Question in the text:** Clinical case 1, question 5 (main text)

#### *QUESTION 2:*

What antibiotic(s) should be used for low-energy open fractures? What antibiotic(s) should be used for high-energy open and grossly-contaminated fractures?

RECOMMENDATION:

1. Antibiotic treatment targeting gram-positive organisms is recommended as soon as possible for all open fractures; low- and high-energy.

LEVEL OF EVIDENCE: 1. Strong

DELEGATE VOTE: Agree: 95%, Disagree: 0%, Abstain: 5% (Unanimous, Strongest Consensus)

#### *QUESTION 5:*

What is the most optimal antibiotic treatment for chronic osteomyelitis?

RECOMMENDATION: Antibiotic selection should be culture-specific, if possible. No clear evidence exists to suggest that longer duration of therapy (12 to 16 weeks) is superior to shorter duration (4 to 6 weeks). In addition, there is no evidence to support the proposition that intravenous (IV) antibiotic treatment is superior to oral treatment.

LEVEL OF EVIDENCE: Limited

DELEGATE VOTE: Agree: 96%, Disagree: 4%, Abstain: 0% (Unanimous, Strongest Consensus)

- **Question in the text:** Clinical case 1, question 4

#### *QUESTION 6:*

What is the recommended suppressive antibiotic therapy for the treatment of chronic osteomyelitis after fracture fixation when the implant cannot be removed?

RECOMMENDATION: Suppressive therapy with culture-specific antibiotics is aimed at allowing fracture healing prior to implant removal and definitive infection management.

LEVEL OF EVIDENCE: Limited

DELEGATE VOTE: Agree: 100%, Disagree: 0%, Abstain: 0% (Unanimous, Strongest Consensus)

- **Question in the text:** clinical case 2, question 5

### 3.3. TREATMENT: RISK FACTORS

#### *QUESTION 1:*

What are predictors of the need for allogeneic blood transfusion (ABT) in periprosthetic fractures?

RECOMMENDATION: Predicting factors for allogeneic blood transfusion are: revision arthroplasty, preoperative anemia, increasing age, higher comorbidity index, lower Body Mass Index (BMI), female gender, longer surgical time and hip surgery.

LEVEL OF EVIDENCE: Limited

DELEGATE VOTE: Agree: 95%, Disagree: 5%, Abstain: 0% (Unanimous, Strongest Consensus)

- **Question in the text:** clinical case 5, question 5

#### *QUESTION 2:*

Is acute femoral neck fracture a risk factor for infection in patients undergoing hip arthroplasty?

RECOMMENDATION: There appears to be a higher incidence of infection in patients undergoing arthroplasty for acute femoral neck fracture compared to hip arthroplasty for primary osteoarthritis. The reported rate of infection has a wide range; prospective studies should be performed to determine the true rate of periprosthetic joint infection (PJI) in this subset of patients.

LEVEL OF EVIDENCE: Limited

DELEGATE VOTE: Agree: 96%, Disagree: 4%, Abstain: 0% (Unanimous, Strongest Consensus)

- **Question in the text:** clinical case 2, question 1

### 3.4. TREATMENT: PROCEDURE-RELATED

#### *QUESTION 1:*

What is the optimal timing of surgical debridement in open fractures?

RECOMMENDATION: It is not possible to establish a clear cut-off for optimal timing of open fracture surgical debridement after injury. Administration of antibiotic prophylaxis and adequacy of debridement is more important than time to debridement. However, we recommend debridement as soon as the patient and operative conditions are optimal.

LEVEL OF EVIDENCE: Limited

DELEGATE VOTE: Agree: 100%, Disagree: 0%, Abstain: 0% (Unanimous, Strongest Consensus)

- **Question in the text:** clinical case 2, question 2

#### *QUESTION 3:*

What is the recommended volume and composition of irrigating fluid in the operating room for open fractures and post-traumatic wounds?

RECOMMENDATION: Irrigation in open fractures should be performed with normal saline and gravity flow irrigation. 3-9L is a reasonable volume to use. Bactericidal washes with agents like chlorhexidine or povidone-iodine have not been adequately studied in orthopaedic trauma patients, but basic science studies raise concern that they may damage tissues.

LEVEL OF EVIDENCE: Moderate

DELEGATE VOTE: Agree: 100%, Disagree: 0%, Abstain: 0% (Unanimous, Strongest Consensus)

- **Question in the text:** clinical case 3, question 3

#### *QUESTION 4:*

What is the most appropriate management of early (prior to complete wound healing) infection after fracture fixation with stable fixation?

RECOMMENDATION: The most acceptable treatment strategy for trauma patients with early postoperative infection is to perform proper irrigation and debridement, administer intravenous (IV) followed by oral antibiotic therapy and retain stable hardware in place.

LEVEL OF EVIDENCE: Moderate

DELEGATE VOTE: Agree: 100%, Disagree: 0%, Abstain: 0% (Unanimous, Strongest Consensus)

- **Question in the text:** clinical case 3, question 2

#### *QUESTION 5:*

What is the most appropriate management of early (before complete wound healing) infection after fracture fixation with unstable fixation?

RECOMMENDATION: The most appropriate management of early (prior to complete healing) infection after fracture fixation with unstable fixation consists of surgical debridement with removal of fixation implants, fracture stabilization, antibiotic therapy and soft tissue coverage, if needed.

LEVEL OF EVIDENCE: Consensus

DELEGATE VOTE: Agree: 100%, Disagree: 0%, Abstain: 0% (Unanimous, Strongest Consensus)

### 3.5. TREATMENT: MANAGEMENT OF HARDWARE

QUESTION 1:

When should hardware be removed when treating surgical site infection (SSI) in orthopaedic trauma?

RECOMMENDATION: The decision to retain or remove hardware differs by clinical scenario and must take into account extent of the infection and stability of the hardware and fracture. A methodical approach that addresses the pathogen, host factors and bony and soft tissue deficiencies is required, and includes thorough debridement,dead-space management and soft tissue and bony reconstruction using the established principles of the reconstruction ladder.

LEVEL OF EVIDENCE: Moderate

DELEGATE VOTE: Agree: 95%, Disagree: 0%, Abstain: 5% (Unanimous, Strongest Consensus)

# **Guideline:** Recommendations for Systemic Antimicrobial Therapy in Fracture-Related Infection: A Consensus From an International Expert Group (Consensus group 2020)

## Key Recommendations on Antimicrobial Therapy

- For the antimicrobial treatment of FRI, the presence of a biofilm, fracture stability, and fracture consolidation are important determinants that should be key determinants in the decision-making process.
- If there are confirmatory or suggestive signs of FRI, empiric intravenous antimicrobial therapy should be started immediately after peroperative tissue sampling.
- Empiric therapy should be broad spectrum including a lipo/glycopeptide and an agent covering Gram-negative bacilli. Thereafter, it should be narrowed according to culture results as soon as possible.
- IV antibiotics can be switched to appropriate bioavailable oral agents—if applicable—as soon as definite culture, and sensitivity results are known. The earlier recommendation of fixed initial 2 weeks of IV antibiotic therapy can be abandoned.
- Targeted antibiotic therapy should be guided by the retrieved pathogens and surgical strategy.
- Expert microbiology/ID physician advice should always be sought especially when there is antimicrobial resistance, intolerance, or risk of drug interactions.
- Antibiotics should be used prudently and in line with the principles of good antimicrobial stewardship.
- **Question in the text:** Clinical case 2, question 3
- **Question in the text:** Clinical case 2, question 4
- **Question in the text:** clinical case 3, question 5
- **Question in the text:** clinical case 4, question 3
- **Question in the text:** clinical case 4, question 4
- **Question in the text:** clinical case 4, question5

**PROSTHETIC JOINT INFECTION (PJI)**

# **Guidelines:** ICM 2018 PJI

# Section 1: Prevention

## 1.1. PREVENTION: HOST RELATED

### QUESTION 1:

What are the absolute and relative contraindications to elective primary total joint arthroplasty (TJA), with respect to surgical site infection (SSI) and periprosthetic joint infection (PJI) risk?

**RECOMMENDATION:** Elective joint arthroplasty is contraindicated in patients with an infectious lesion in the ipsilateral extremity, until the infection is resolved. TJA needs to be deferred in patients with uncontrolled conditions such as diabetes, malnutrition, chronic kidney disease, as well as other diseases that are known to increase the risks of SSIs/PJIs.

Risk factors (Evidence Strength: Strong):

- Intra-articular Injections (Modifiable): strong evidence that surgery should be absolutely delayed for a minimum of three months following intra-articular steroid injections.
- Obesity (Modifiable): obesity is considered a relative contraindication while morbid-obesity (BMI >40) serves as an absolute contraindication.
- Bariatric Surgery (Non-modifiable): It is suspected that in patients undergoing bariatric surgery prior to TJA, the risks for PJIs are reduced due to decreasing BMIs, but is off set by the increased risk for malnutrition.
- Malnutrition (Modifiable): severe malnutrition (serum albumin < 3 g/dL), however, should be an absolute contraindication.
- Diabetes Mellitus (Modifiable): severely uncontrolled diabetes is an absolute contraindication for TJA (e.g., serum glucose ≥ 200 mg/dL).
- Chronic Kidney Disease (CKD) (Modifiable): With the reduced risks for postoperative SSIs/ PJIs, patients on hemodialysis should be evaluated for renal transplant prior to TJAs.
- Previous Infection of the Operative Joint (Non-modifi able): Patients reporting previous infections of the joint should be evaluated for active infections with erythrocyte sedimentation rate (ESR) and C-reactive protein (CRP). Surgery should be delayed for those with markers of active infections.
- Active Infection (Modifiable): to prevent the catastrophic sequelae of PJIs, active infections of the joint, bloodstream or local tissue are an absolute contraindication to surgery and should be managed prior to performing a TJA.
- Smoking (modifiable): Smoking represents an independent, modifiable risk factor that significantly compounds the risks of SSIs/PJIs when present alongside other comorbidities.
- Hip vs. Knee Arthroplasty (Non-modifiable): Compared to THAs, TKAs were consistently associated with increased risk of PJIs/SSIs
- Socioeconomic Status (Non-modifiable): Consistent evidence showed that a low income was associated with increased risks of PJIs/SSIs
- Depression (Modifiable):Evidence suggested histories of depression and psychosis to be associated with increased risks of PJIs following TJA

**LEVEL OF EVIDENCE:** Strong

**DELEGATE VOTE:** Agree: 90%, Disagree: 7%, Abstain: 3% (Super Majority, Strong Consensus)

- **Question in the text:** clinical case 2, question 1
- **Question in the text:** Clinical case 5, question 4

### QUESTION 2:

Is the diagnosis of post-traumatic arthritis associated with increased risks of subsequent surgical site infections/periprosthetic joint infections (SSIs/PJIs) after joint arthroplasty?

**RECOMMENDATION:** Yes. Total joint arthroplasty (TJA) for patients with post-traumatic arthritis of the hip or knee carries higher risks of developing SSIs/PJIs. The incidence is markedly higher in patients with previous surgeries and retained implants.

**LEVEL OF EVIDENCE:** Moderate

**DELEGATE VOTE:** Agree: 97%, Disagree: 1%, Abstain: 2% (Unanimous, Strongest Consensus)

### QUESTION 3:

What nutritional markers are the most sensitive and specific for surgical site infections and periprosthetic infections (SSIs/PJIs)? Does improvement in nutritional status reduce the risk of SSI/PJI?

**RECOMMENDATION:** Serum albumin < 3.5 g/dL has been demonstrated to be an independent risk factor for SSIs/PJIs following total joint arthroplasty in multiple, large-scale studies. However, other nutritional markers are poorly studied. Currently, there is insufficient evidence to prove that correction of preoperative nutritional markers reduces the risks of subsequent SSIs/PJIs. Despite the absence of such evidence, we recognize the importance of an optimized nutritional status before total joint arthroplasty (TJA) to reduce the risks of SSIs/PJIs.

**LEVEL OF EVIDENCE:** Moderate

**DELEGATE VOTE:** Agree: 98%, Disagree: 1%, Abstain: 1% (Unanimous, Strongest Consensus)

## 1.2. PREVENTION: RISK MITIGATION

### QUESTION 1:

What preoperative screening for infections should be performed in patients undergoing revision hip or knee arthroplasty because of presumed aseptic failure?

**RECOMMENDATION:** In addition to taking a thorough history, obtaining radiographic imaging and performing a physical examination, all patients with a failed hip or knee arthroplasty awaiting revision surgery should have their serum erythrocyte sedimentation rate (ESR) and C-reactive protein (CRP) measured. Patients with high index of suspicion for infection should be considered for further workup.

**LEVEL OF EVIDENCE:** Moderate

**DELEGATE VOTE:** Agree: 96%, Disagree: 4%, Abstain: 0% (Unanimous, Strongest Consensus)

## 1.3. PREVENTION: ANTIMICROBIALS (SYSTEMIC)

### QUESTION 1:

What is the most appropriate perioperative prophylactic antibiotic (agent, route and number of doses) for patients undergoing primary total joint arthroplasty (TJA) to reduce the risk of subsequent surgical site infections/periprosthetic joint infections (SSIs/PJIs)?

**RECOMMENDATION:** The most appropriate perioperative prophylactic antibiotic is a fi rst or second-generation cephalosporin (i.e., cefazolin or cefuroxime) administered intravenously within 30 to 60 minutes prior to incision as a single- and weight-adjusted dose.

**LEVEL OF EVIDENCE:** Strong

**DELEGATE VOTE:** Agree: 90%, Disagree: 8%, Abstain: 2% (Super Majority, Strong Consensus)

### QUESTION 2:

What are the appropriate weight-adjusted prophylactic antibiotic dosages?

**RECOMMENDATION:** The recommended weight-adjusted doses of antimicrobials for prophylaxis of hip and knee arthroplasty in adults are shown in Table 1.

**TABLE 1. Recommended weight-adjusted doses of antimicrobials for prophylaxis of hip and knee arthroplasty in adults**

*Actual body weight.

#No recommended adjustment for weight.

**LEVEL OF EVIDENCE:** Moderate

**DELEGATE VOTE:** Agree: 92%, Disagree: 4%, Abstain: 4% (Super Majority, Strong Consensus)

### QUESTION 3:

Is one dose of preoperative antibiotic adequate for patients undergoing total joint arthroplasty (TJA)?

**RECOMMENDATION:** Despite the current guidelines from the Centers for Disease Control and Prevention (CDC) advocating for a single dose of perioperative antibiotics, these studies are underpowered and primarily in specialties outside orthopedics. From the limited evidence available, it appears that a single perioperative dose of antibiotics, compared to multiple doses, does not increase the rate of subsequent surgical site infections/periprosthetic joint infections (SSIs/PJIs). A randomized prospective study in patients undergoing elective arthroplasty is underway that should answer this question definitively.

**LEVEL OF EVIDENCE:** Limited

**DELEGATE VOTE:** Agree: 92%, Disagree: 7%, Abstain: 3% (Super Majority, Strong Consensus)

### QUESTION 4:

Should patients undergoing outpatient total joint arthroplasty (TJA) receive additional postoperative prophylactic antibiotics?

**RECOMMENDATION:** Despite the current guidelines from the Centers for Disease Control and Prevention (CDC) advocating for a single dose of perioperative antibiotics, the studies utilized to form these guidelines are underpowered and primarily in specialties outside orthopaedics. The limited evidence suggests that a single perioperative dose of antibiotics, compared to multiple doses, does not increase the rates of subsequent surgical site infections/periprosthetic joint infections (SSIs/PJIs). A randomized prospective study in patients undergoing elective arthroplasty is underway, which should help answer this question definitively.

**LEVEL OF EVIDENCE:** Limited

**DELEGATE VOTE:** Agree: 94%, Disagree: 4%, Abstain: 2% (Super Majority, Strong Consensus)

### QUESTION 6:

Should duration and the type of antibiotic prophylaxis be altered in patients with a prior periprosthetic joint infection (PJI)?

**RECOMMENDATION:** Antibiotic prophylaxis should be tailored in patients with prior PJIs who are undergoing another subsequent elective primary or revision joint arthroplasty. Antibiotic prophylaxis should cover the initial causative organism(s) as well as the most common pathogens that can cause PJI with either single or dual antibiotics.

**LEVEL OF EVIDENCE:** Limited

**DELEGATE VOTE:** Agree: 93%, Disagree: 6%, Abstain: 1% (Super Majority, Strong Consensus)

### QUESTION 8:

Does the use of allografts alter the recommended duration of prophylactic antibiotics?

**RECOMMENDATION:** No. Allografts are avascular materials that are prone to contamination and may serve as a scaff old for bacterial colonization and biofilm production, similar to a prosthesis or osteosynthesis material. However, it is difficult to establish a causal relationship between the use of an allograft and subsequent infection. Thus, there is no evidence to support the use of extended antibiotic prophylaxis.

**LEVEL OF EVIDENCE:** Limited

**DELEGATE VOTE:** Agree: 91%, Disagree: 6%, Abstain: 3% (Super Majority, Strong Consensus)

- **Question in the text:** clinical case 1, question 1

## 1.4. PREVENTION: ANTIMICROBIALS (LOCAL)

### QUESTION 3:

What is the optimal antibiotic(s) dosage to be used in cement during reimplantation that does not significantly interfere with the mechanical strength of cement used for fixation?

**RECOMMENDATION:** The mechanical strength of most cement is maintained if ≤5% (w/w) of antibiotics is added (equating to 2 grams in a 40 gram packet).

**LEVEL OF EVIDENCE:** Moderate

**DELEGATE VOTE:** Agree: 92%, Disagree: 3%, Abstain: 5% (Super Majority, Strong consensus)

## 1.5. PREVENTION: OPERATING ROOM ENVIRONMENT

### QUESTION 1:

Does performing a primary total joint arthroplasty (TJA) after a dirty case (infection or open abdomen) in the same operating room increase the risk of surgical site infections/periprosthetic joint infections (SSIs/PJIs)?

**RECOMMENDATION:** The litt le data on this subject suggests that the risk of PJIs may be higher when an elective arthroplasty follows a contaminated case. The risk may be reduced if terminal cleaning of the operating room can be done after the dirty case. Further studies are necessary to elucidate this connection.

**LEVEL OF EVIDENCE:** Limited

**DELEGATE VOTE:** Agree: 93%, Disagree: 4%, Abstain: 3% (Super Majority, Strong Consensus)

### QUESTION 4:

Does changing the drapes during debridement, antibiotics and implant retention (DAIR) affect the rate of success?

**RECOMMENDATION:** The impact and eff ectiveness of changing the drapes during DAIR has not been investigated and therefore it can be performed at the surgeon’s discretion.

**LEVEL OF EVIDENCE:** Consensus

**DELEGATE VOTE:** Agree: 94%, Disagree: 5%, Abstain: 1% (Super Majority, Strong Consensus)

### QUESTION 6:

Does routine use of a new set of surgical instruments and equipment following debridement and before reimplantation reduce the risk of surgical site infections/periprosthetic joint infections (SSIs/PJIs) recurrences? Is it necessary to change all surgical fi elds before the fi nal reimplantation in septic revision surgery?

**RECOMMENDATION:** The change of the surgical fi eld following debridement of an infected joint leads to a reduction in the bioburden and stands to improve outcome of surgical intervention and should be considered.

**LEVEL OF EVIDENCE:** Limited

**DELEGATE VOTE:** Agree: 90%, Disagree: 7%, Abstain: 3% (Super Majority, Strong Consensus)

### QUESTION 7:

Is there a concern for contamination of the surgical fi eld by particles, such as cement, that may escape the wound intraoperatively by coming into contact with the ceiling light or facial masks and fall back into the wound?

**RECOMMENDATION:** There is logically a high risk that particles which fall into the wound after coming into contact with unsterile equipment (e.g., ceiling lights, facial masks) will contaminate the surgical field. However, no studies investigating this hypothesis directly exist in current literature. We recommend that surgeons must be conscious of, and take precautions, in order to prevent particles from falling into the surgical fi eld, and should such a scenario arise, to use copious antiseptic solutions, such as dilute betadine, in order to irrigate the wound.

**LEVEL OF EVIDENCE:** Limited

**DELEGATE VOTE:** Agree: 97%, Disagree: 2%, Abstain: 1% (Unanimous, Strongest Consensus)

## 1.6. PREVENTION: SURGICAL TECHNIQUE

### QUESTION 2:

Does the surgical approach (parapatellar vs. subvastus) during primary total knee arthroplasty (TKA) aff ect the incidence of subsequent surgical site nfections/periprosthetic joint infections (SSIs/PJIs)?

**RECOMMENDATION:** The incidence of SSIs/PJIs after primary TKA is not influenced by the surgical approach (parapatellar vs. subvastus).

**LEVEL OF EVIDENCE:** Moderate

**DELEGATE VOTE:** Agree: 97%, Disagree: 1%, Abstain: 2% (Unanimous, Strongest Consensus)

### QUESTION 4:

Does the use of periarticular injections (PAIs) affect the rate of surgical site infections/periprosthetic joint infections (SSIs/PJIs) recurrence in reimplantation?

**RECOMMENDATION:** Unknown. PAIs are an effective adjunct treatment for pain control following primary total joint arthroplasty (TJA), but their effectiveness and impact on the rates of SSIs/PJIs in the revision setting has not been investigated. The use of PAIs at the time of reimplantation can be performed at the surgeon’s discretion.

**LEVEL OF EVIDENCE:** Limited

**DELEGATE VOTE:** Agree: 91%, Disagree: 5%, Abstain: 4% (Super Majority, Strong Consensus)

## 1.7. PREVENTION: PROSTHESIS FACTORS

### QUESTION 2:

Does the type of fixation of an arthroplasty component influence the incidence of subsequent surgical site infections/periprosthetic joint infections (SSIs/PJIs)?

**RECOMMENDATION:** There is no difference in the rates of SSIs/PJIs after total hip arthroplasty (THA) or total knee arthroplasty (TKA) based on fixation of the prosthesis.

**LEVEL OF EVIDENCE:** Moderate

**DELEGATE VOTE:** Agree: 93%, Disagree: 5%, Abstain: 2% (Super Majority, Strong Consensus)

### QUESTION 6:

Can implant factors (i.e., type of bearing) influence the thresholds for serum and synovial markers in acute and chronic periprosthetic joint infections (PJIs)?

**RECOMMENDATION:** Yes. Different bearing surfaces such as metal-on-metal (MoM), metal-on-polyethylene and dual taper modular stems in the sett ing of taper corrosion can influence the serum and synovial markers. Metal debris may interfere with automated cell counts. Manual cell counts are preferred when evaluating patients for PJIs who have elevated synovial fluid metal levels. Optimal thresholds for serum and synovial

markers for diagnosing PJIs in these settings still need to be established.

**LEVEL OF EVIDENCE:** Moderate

**DELEGATE VOTE:** Agree: 97%, Disagree: 1%, Abstain: 2% (Unanimous, Strongest Consensus)

### QUESTION 7:

What can be done with a prosthesis that has been dropped on the floor or allowed to come into contact with a non-sterile portion of the operating room?

**RECOMMENDATION:** Cleaning, re-sterilization and reuse of dropped prostheses or implants is not permitted in most hospitals and should not be performed. Only in extremely rare circumstances, such as the use of a custom implant, a dropped prosthesis may be decontaminated and sterilized.

**LEVEL OF EVIDENCE:** Consensus

**DELEGATE VOTE:** Agree: 90%, Disagree: 8%, Abstain: 2% (Super Majority, Strong Consensus)

## 1.8. PREVENTION: POSTOPERATIVE ISSUES

### QUESTION 1:

Should patients with cellulitis following total joint arthroplasty be treated with antibiotic therapy?

**RECOMMENDATION:** Yes. When periprosthetic joint infection (PJI) has been ruled out, it is reasonable to treat patients presenting with cellulitis with empiric antibiotics.

**LEVEL OF EVIDENCE:** Moderate

**DELEGATE VOTE:** Agree: 94%, Disagree: 4%, Abstain: 2% (Super Majority, Strong Consensus)

# Section 2: Diagnosis

## 2.1. DIAGNOSIS: DEFINITIONS

### QUESTION 2:

What is the definition of septic arthritis in a native knee?

**RECOMMENDATION:** Native septic arthritis of the knee is a clinical diagnosis supplemented by relevant laboratory data. Signs of septic arthritis include painful effusion, limited range of motion and warmth. Elevated serum inflammatory markers, particularly C-reactive protein (CRP), synovial white blood cell (WBC) counts (50,000 cells/mm3), polymorphonuclear (PMN) cell count percentages (> 90%) and purulent appearance of the synovial fluid indicate a high likelihood of septic arthritis.

**LEVEL OF EVIDENCE:** Moderate

**DELEGATE VOTE:** Agree: 92%, Disagree: 7%, Abstain: 1% (Super Majority, Strong Consensus)

### QUESTION 3:

How can superficial surgical site infections (SSIs) be differentiated from deep SSIs (i.e., periprosthetic joint infections (PJIs))?

**RECOMMENDATION:** There is no single objective clinical test or imaging approach established for the differentiation between a superficial SSI, a deep SSI and a PJI. We recommend that clinical evaluation, workup for infection and early joint aspiration should guide the decision.

**LEVEL OF EVIDENCE:** Limited

**DELEGATE VOTE:** Agree: 96%, Disagree: 3%, Abstain: 1% (Unanimous, Strongest Consensus)

- **Question in the text:** clinical case 1, question 4
- **Question in the text:** clinical case 2, question 2

### QUESTION 5:

What clinical findings (e.g., fever, erythema, reduced range of motion) are most sensitive and specific for the diagnosis of periprosthetic joint infections (PJIs)?

**RECOMMENDATION:** A painful prosthetic joint is the most sensitive, but least specific clinical finding in PJIs. Signs of deep tissue involvement (i.e., sinus tract, purulence, abscess and extensive necrosis) are the most specific signs. It is important to note that clinical findings diff er notably based on the type of joint involved (hip or knee), as well as to the timing and presentation of PJIs (i.e., early postoperative, acute hematogenousand chronic).

**LEVEL OF EVIDENCE:** Moderate

**DELEGATE VOTE:** Agree: 97%, Disagree: 1%, Abstain: 2% (Unanimous, Strongest Consensus)

- **Question in the text:** clinical case 1, question 2
- **Question in the text:** clinical case 1, question 3

### QUESTION 7:

Is aseptic loosening (AL) associated with undiagnosed periprosthetic joint infections (PJIs)?

**RECOMMENDATION:** Some percentage of AL is due to culture-negative infection, since up to 10% of culture-negative cases contain bacteria when screened by molecular methods. Whether this correlates to an undiagnosed infection causing AL remains unclear. Understanding this issue is limited by the ability of bacterial culture to function as an effective gold standard for detecting infection. The role of molecular techniques such as next generation sequencing in this setting needs to be explored.

**LEVEL OF EVIDENCE:** Limited

**DELEGATE VOTE:** Agree: 90%, Disagree: 8%, Abstain: 2% (Super Majority, Strong Consensus)

## 2.2. DIAGNOSIS: ALGORITHM

### QUESTION 2:

Are there any contraindications to knee or hip aspiration prior to revision surgery?

**RECOMMENDATION:** There are no clearly identified contraindications to aspiration of the knee or hip joint performed as part of the patient workup for infection.

**LEVEL OF EVIDENCE:** Limited

**DELEGATE VOTE:** Agree: 90%, Disagree: 8%, Abstain: 2% (Super Majority, Strong Consensus)

- **Question in the text:** clinical case 3, question 1

### QUESTION 4:

In patients with multiple arthroplasties in place who have developed a periprosthetic infection (PJI) of one joint, should other joints be investigated for PJIs also?

**RECOMMENDATION:** We recommend that when a patient develops a PJI in one joint, the other total joint arthroplasties (TJAs) should be examined clinically and if suspicion for PJI remains, or the patient is immunocompromised, then other joints should be aspirated.

**LEVEL OF EVIDENCE:** Limited

**DELEGATE VOTE:** Agree: 92%, Disagree: 6%, Abstain: 2% (Super Majority, Strong Consensus)

- **Question in the text:** clinical case 3, question 4

### QUESTION 6:

What is the prevalence of culture-negative periprosthetic join infections (CN-PJIs) and what are the diagnostic protocols for further investigating these cases?

**RECOMMENDATION:** The reported prevalence of CN-PJIs in the hip or knee has ranged from 5-42%. Diagnostic protocols for further investigating these cases include repeat sampling, longer incubation of culture samples, sonication of implants, the use of dithiothreitol (DTT) technology, polymerase chain reaction (PCR) and next generation sequencing (NGS).

**LEVEL OF EVIDENCE:** Moderate

**DELEGATE VOTE:** Agree: 91%, Disagree: 8%, Abstain: 1% (Super Majority, Strong Consensus)

- **Question in the text:** clinical case 3, question 3

### QUESTION 7:

Do patients with adverse local tissue reactions (ALTRs) have a higher incidence of periprosthetic joint infections (PJIs)?

**RECOMMENDATION:** Yes. Patients with ALTRs appear to have a higher incidence of PJIs.

**LEVEL OF EVIDENCE:** Moderate

**DELEGATE VOTE:** Agree: 95%, Disagree: 2%, Abstain: 3% (Unanimous, Strongest Consensus)

### QUESTION 8:

Should we routinely assess for serum/blood metal ion levels (cobalt (Co) and chromium (Cr)) when working up a patient with a painful total joint arthroplasty?

**RECOMMENDATION:** There is no data to suggest routine assessment of serum/blood metal ion levels (CoCr) in all patients with painful joint arthroplasty. There may be a rationale for second-line assessment of metal levels in painful metal-on-metal (MoM) total hip arthroplasty (THA), hip resurfacing, modular neck femoral components and in certain metal-on-polyethylene (MoP) THA in which trunnion corrosion is suspected.

**LEVEL OF EVIDENCE:** Limited

**DELEGATE VOTE:** Agree: 92%, Disagree: 4%, Abstain: 4% (Super Majority, Strong Consensus)

## 2.3. DIAGNOSIS: LABORATORY TESTS

### QUESTION 2:

Does the presence of both an erythrocyte sedimentation rate (ESR) and C-reactive protein (CRP) below the periprosthetic joint infection (PJI) thresholds rule out the diagnosis of a PJI?

**RECOMMENDATION:** Serum ESR and CRP levels below the threshold (as determined by the MusculoSkeletal Infection Society (MSIS) and International Consensus Meeting (ICM)) does not exclude the diagnosis of a PJI. Serum levels of ESR and CRP can be normal in some cases of PJI caused by slow-growing organisms.

**LEVEL OF EVIDENCE:** Moderate

**DELEGATE VOTE:** Agree: 100%, Disagree: 0%, Abstain: 0% (Unanimous, Strongest Consensus)

- **Question in the text:** clinical case 3, question 2

### QUESTION 4:

How does the level of leukocyte count and neutrophil percentage in the synovial fluid change with time following total joint arthroplasty?

**RECOMMENDATION:** The levels of leukocyte count and neutrophil percentage in the synovial fluid drop as one moves further away from the index arthroplasty. The latter is the rationale behind using different thresholds for these parameters in the diagnosis of acute versus chronic periprosthetic joint infections (PJIs).

**LEVEL OF EVIDENCE:** Limited

**DELEGATE VOTE:** Agree: 90%, Disagree: 4%, Abstain: 6% (Super Majority, Strong Consensus)

## 2.4. DIAGNOSIS: PATHOGEN ISOLATION, CUTURE RELATED

### QUESTION 2:

Are there significant differences in the yield of culture between preoperative aspiration and intraoperative culture samples? If so, which result should be utilized?

**RECOMMENDATION:** There may be differences in the yield of culture between preoperative aspiration and intraoperative culture samples, particularly in the case of polymicrobial infections or low-virulence organisms. The collection of multiple intraoperative tissue samples is considered by many experts to provide the highest yield in isolating organisms from a joint.

**LEVEL OF EVIDENCE:** Limited

**DELEGATE VOTE:** Agree: 98%, Disagree: 1%, Abstain: 1% (Unanimous, Strongest Consensus)

### QUESTION 3:

Do bone cultures provide additional diagnostic accuracy in the diagnosis of periprosthetic joint infections (PJIs)?

**RECOMMENDATION:** Inconclusive. We cannot recommend for or against bone biopsy to provide additional diagnostic accuracy in the diagnosis of PJIs.

**LEVEL OF EVIDENCE:** Limited

**DELEGATE VOTE:** Agree: 90%, Disagree: 5%, Abstain: 5% (Super Majority, Strong Consensus)

### QUESTION 4:

Is there a role for obtaining cultures before, and at the time of, insertion of prosthesis during second stage (reimplantation) of a two-stage exchange arthroplasty?

**RECOMMENDATION:** Preoperative aspiration of a joint should be determined based on the index of suspicion for persistent infection. During reimplantation, however, multiple fluid and tissue samples should be sent for culture. There is a direct correlation between the outcome of two stage exchange arthroplasty and culture results during reimplantation.

**LEVEL OF EVIDENCE:** Moderate

**DELEGATE VOTE:** Agree: 95%, Disagree: 4%, Abstain: 1% (Unanimous, Strongest Consensus)

- **Question in the text:** clinical case 5, question 3

## 2.5. DIAGNOSIS: REIMPLANTATION

### QUESTION 2:

What metrics should be considered to determine the timing of reimplantation after two-stage exchange arthroplasty of the infected hip or knee?

**RECOMMENDATION:** There are no definitive metrics to allow determination of optimal timing of reimplantation. Thus, timing of reimplantation should consider resolution of clinical signs of infection, down-trend in the serological markers and results of synovial analysis, if aspiration is performed.

**LEVEL OF EVIDENCE:** Moderate

**DELEGATE VOTE:** Agree: 96%, Disagree: 3%, Abstain: 1% (Unanimous, Strongest Consensus)

### QUESTION 3:

Is normalization of serological markers necessary prior to reimplantation arthroplasty performed as part of a two-stage exchange?

**RECOMMENDATION:** No. A trend and decline in C-reactive protein (CRP) and erthyrocyte sedimentation rate (ESR) is expected, but we still recognize that there are certain cases in which reimplantation may be performed despite abnormal levels of ESR and CRP. Surgeons should not wait for complete normalization of the inflammatory markers as this may not occur in some patients and/or take a long period of time.

**LEVEL OF EVIDENCE:** Moderate

**DELEGATE VOTE:** Agree: 95%, Disagree: 4%, Abstain: 1% (Unanimous, Strongest Consensus)

- **Question in the text:** clinical case 1, question 6

### QUESTION 4:

What is the importance of two-week antibiotic holiday prior to reimplantation?

**RECOMMENDATION:** Unknown. There is no conclusive evidence to support the need or the ideal length of an antibiotic holiday prior to reimplantation.

**LEVEL OF EVIDENCE:** Limited

**DELEGATE VOTE:** Agree: 92%, Disagree: 7%, Abstain: 1% (Super Majority, Strong Consensus)

### QUESTION 5:

What is the diagnostic accuracy of joint aspiration of a cement spacer in conjunction with clinical evaluation, imaging, serologic tests, and biopsies? Should it routinely be performed prior to reimplantation?

**RECOMMENDATION:** The diagnostic accuracy of joint aspiration prior to reimplantation is not known. None of the parameters being used to diagnose periprosthetic joint infection (PJI), and their respective thresholds, have been determined for aspiration. The decision to perform aspiration should be made based on the index of suspicion for persistent infection and individualized.

**LEVEL OF EVIDENCE:** Moderate

**DELEGATE VOTE:** Agree: 95%, Disagree: 4%, Abstain: 1% (Unanimous, Strongest Consensus)

# Section 3: Pathogen factors

## QUESTION 2:

Is there a difference in the treatment outcome for periprosthetic joint infections (PJIs) caused by a single organism and a polymicrobial PJI?

**RECOMMENDATION:** Polymicrobial PJIs demonstrate inferior treatment outcomes when compared to monomicrobial PJIs. This finding is true for both patients treated with irrigation and debridement and two-stage exchange arthroplasty.

**LEVEL OF EVIDENCE:** Moderate

**DELEGATE VOTE:** Agree: 97%, Disagree: 3%, Abstain: 0% (Unanimous, Strongest Consensus)

- **Question in the text:** clinical case 4, question 2

## QUESTION 3:

Is there a difference in the type of pathogens that can cause surgical site infections/periprosthetic joint infections (SSIs/PJIs) between hip and knee arthroplasty?

**RECOMMENDATION:** There is limited evidence to support a difference in the organism profile causing SSIs and PJIs between hip and knee arthroplasty. Isolated studies have reported an increased prevalence of *Streptococcal* and culture-negative PJI around the knee, whereas *Staphylococcal, Enterococcal, Pseudomonal* PJIs may be more prevalent around the hip. Further work regarding the different flora in these respective body regions is needed, as it may determine antibiotic selection.

**LEVEL OF EVIDENCE:** Limited

**DELEGATE VOTE:** Agree: 92%, Disagree: 4%, Abstain: 4% (Super Majority, Strong Consensus)

## QUESTION 4:

Is there a difference in the organism profile that causes periprosthetic joint infections (PJIs) in different countries?

**RECOMMENDATION:** Yes, there is a difference in the organism profile causing PJIs in diff erent countries and regions of this world. There seems to be a higher incidence of PJI caused by methicillin-resistant *Staphylococcus aureus* (MRSA) in the United States and Australia compared to Europe.

**LEVEL OF EVIDENCE:** Moderate

**DELEGATE VOTE:** Agree: 97%, Disagree: 1%, Abstain: 2% (Unanimous, Strongest Consensus)

- **Question in the text:** clinical case 4, question 5

# Section 4: Fungal Periprosthetic joint infection

## 4.1. FUNGAL PERIPROSTHETIC JOINT INFECTION: DIAGNOSIS AND TREATMENT

### QUESTION **1:**

### What is the optimal method to diagnose fungal periprosthetic joint infection (PJI)?

**RECOMMENDATION:** Diagnosis of fungal PJIs is established by incubating joint aspirations or tissue samples collected intraoperatively on specialized culture media. Furthermore, isolation of fungal species may take up to four weeks. However, given the shortcomings associated with the use of culture, alternative techniques capable of detecting fungi, such as molecular techniques, may be used as an adjunct.

**LEVEL OF EVIDENCE:** Moderate

**DELEGATE VOTE:** Agree: 95%, Disagree: 3%, Abstain: 2% (Unanimous, Strongest Consensus)

### QUESTION 2:

Should patients with periprosthetic joint infections (PJIs) caused by a fungus undergo the typical two-week antimicrobial holiday prior to reimplantation?

**RECOMMENDATION:** There is no conclusive evidence to support the use of an antimicrobial holiday period prior to reimplantation in case of fungal PJI treated with staged revision.

**LEVEL OF EVIDENCE:** Limited

**DELEGATE VOTE:** Agree: 90%, Disagree: 5%, Abstain: 5% (Super Majority, Strong Consensus)

### QUESTION 3:

Can debridement, antibiotics and implant retention (DAIR) be used to treat acute fungal periprosthetic joint infections (PJIs)?

**RECOMMENDATION:** DAIR has a relatively high failure rate in fungal PJIs, especially for immunocompromised patients. DAIR should be reserved for patients with truly acute PJIs after an index arthroplasty and in healthy patients (Type A). If DAIR is performed for fungal PJIs, consideration should be given to anti-fungal suppression therapy.

**LEVEL OF EVIDENCE:** Moderate

**DELEGATE VOTE:** Agree: 91%, Disagree: 5%, Abstain: 4% (Super Majority, Strong Consensus)

### QUESTION 4:

Which antifungals, route of administration and duration of treatment should be utilized to treat fungal periprosthetic joint infections (PJIs)?

**RECOMMENDATION:** Fluconazole, by both oral and intravenous routes, is currently the treatment of choice for PJIs due to susceptible fungi, including the *Candida* species which are responsible for the majority of fungal PJI cases. Amphotericin B lipid formulations or echinocandins given intravenously are secondary considerations, but may be less well tolerated. Culture data including antifungal susceptibilities should be used to guide therapy. Two-stage revision is currently the standard of care. Antifungal treatment should be administered during the spacer interval with a minimum treatment duration of six weeks. Following revision, treatment with oral fluconazole (400mg daily) should be continued for three to six months, if tolerated.

**LEVEL OF EVIDENCE:** Limited

**DELEGATE VOTE:** Agree: 92%, Disagree: 3%, Abstain: 5% (Super Majority, Strong Consensus)

# Section 5: Treatment

## 5.1. TREATMENT: ALGORITHM

### QUESTION 1:

Should early postoperative infection and acute hematogenous infection be treated and managed differently?

**RECOMMENDATION:** There is no evidence to support the notion that early postoperative infection and acute hematogenous infection should be treated differently as long as the onset of symptoms is <4 weeks (favorable <_ 7 days), implants are well-fixed, no sinus tract exists, and the isolated infecting organism is sensitive to an antimicrobial agent.

**LEVEL OF EVIDENCE:** Moderate

**DELEGATE VOTE:** Agree: 94%, Disagree: 5%, Abstain: 1% (Super Majority, Strong Consensus)

## 5.2. TREATMENT: DEBRIDEMENT AND RETENTION OF IMPLANT

### QUESTION 2:

Is debridement, antibiotics and implant retention (DAIR) an emergency procedure for patients with acute periprosthetic joint infection(PJI) or should patient optimization be implemented prior to surgery to enhance the success of this procedure?

**RECOMMENDATION:** DAIR is not an emergency procedure but should be performed on an urgent basis when the patient with acute PJI is medically and surgically optimized.

**LEVEL OF EVIDENCE:** Limited

**DELEGATE VOTE:** Agree: 97%, Disagree: 3%, Abstain: 0% (Unanimous, Strongest Consensus)

### QUESTION 3:

Does identification of the pathogen prior to performing debridement, antibiotics and implant retention (DAIR) help guide the surgeon’s decision making? If so, should you wait,

in a clinically stable patient, until the pathogen has been identified?

**RECOMMENDATION:** The identification of the responsible microorganism before DAIR is desirable. However, it should not prevent timely surgical intervention if delay in surgery is believed to promote further establishment of biofilm formation and compromise the outcome of surgical intervention.

**LEVEL OF EVIDENCE:** Limited

**DELEGATE VOTE:** Agree: 94%, Disagree: 4%, Abstain: 2% (Super Majority, Strong Consensus)

### QUESTION 4:

Does exchange of all modular components during debridement, antibiotic and implant retention (DAIR) reduce the rate of surgical site infection (SSI)/periprosthetic joint infection (PJI) recurrence?

**RECOMMENDATION:** Yes. Exchange of all the modular components during DAIR reduces the risk of PJI recurrence.

**LEVEL OF EVIDENCE:** Moderate

**DELEGATE VOTE:** Agree: 94%, Disagree: 4%, Abstain: 2% (Super Majority, Strong Consensus)

### QUESTION 5:

What is the minimum necessary volume of irrigation solution to use in debridement, antibiotics and implant retention (DAIR) treatment of acute periprosthetic joint infection (PJI)?

**RECOMMENDATION:** We recommend that 6-9L of irrigation solution, including saline or antiseptic solution such as sterile dilute povidoneiodine, is used during DAIR treatment of acute PJI.

**LEVEL OF EVIDENCE:** Consensus

**DELEGATE VOTE:** Agree: 90%, Disagree: 7%, Abstain: 3% (Super Majority, Strong Consensus)

- **Question in the text:** clinical case 2, question 4

### QUESTION 6:

Is there a role for direct intra-articular antibiotic infusion following irrigation and debridement (I&D) for periprosthetic joint infection (PJI)?

**RECOMMENDATION:** The concept of achieving a minimum biofi lm eradication concentration (MBEC) of antibiotics at the site of the infection is compelling. Despite the presence of retrospective studies reporting favorable outcome, because of heterogeneity in terms of adjunctive antibiotics, absence of a control group and small cohort size, the routine administration of intra-articular antibiotics in treatment of PJI is not justified. Prospective, randomized controlled trials (RCTs) are needed to support the routine use of intra-articular antibiotics as a stand-alone or adjunct treatment of PJI.

**LEVEL OF EVIDENCE:** Consensus

**DELEGATE VOTE:** Agree: 92%, Disagree: 6%, Abstain: 2% (Super Majority, Strong Consensus)

### QUESTION 7:

Can debridement, antibiotics and implant retention (DAIR) be utilized in patients with an acute chronic infection of a unicompartmental knee arthroplasty (UKA)?

**RECOMMENDATION:** In the event of acute infection following UKA, early DAIR can be considered. However, if initial treatment eff ort results in failure or chronic infection is present, the implanted prosthesis should be removed and a one-stage or two-stage conversion to total knee arthroplasty (TKA) should be performed in combination with antibiotic therapy.

**LEVEL OF EVIDENCE:** Limited

**DELEGATE VOTE:** Agree: 96%, Disagree: 2%, Abstain: 2% (Unanimous, Strongest Consensus)

### QUESTION 8:

Can debridement, antibiotics and implant retention (DAIR) be utilized in the treatment of acute periprosthetic joint infection (PJI) with a megaprosthesis?

**RECOMMENDATION:** DAIR is a viable treatment option in acute PJI of a megaprosthesis. The effectiveness of DAIR is still unclear due to lack of comparative data among the treatment options and limited evidence to suggest superiority of any one treatment. The treatment decision must be made on a case-by-case basis and account for underlying medical conditions, infection history, organism characteristics and surgical history. DAIR is most appropriate for acute PJI without complicating factors, such as extensive and pervasive infection by a high virulence or resistant organism.

**LEVEL OF EVIDENCE:** Limited

**DELEGATE VOTE:** Agree: 96%, Disagree: 1%, Abstain: 3% (Unanimous, Strongest Consensus)

### QUESTION 9:

What factors are associated with the successful treatment of acute periprosthetic joint infection (PJI) using debridement, antibiotics and implant retention (DAIR)?

**RECOMMENDATION:** The following factors have been shown to be associated with treatment success in acute PJIs treated with DAIR:

• Exchanging the modular components during debridement

• Performing a debridement within at least seven days, but preferably as soon as possible, after the onset of symptoms

• Adding rifampin to the antibiotic regimen, particularly when combined with a fluoroquinolone, in cases of susceptible staphylococci

• Treatment with fluoroquinolones in cases of susceptible gram-negative bacilli

The following factors have been shown to be associated with treatment failure in acute PJIs treated with DAIR:

• Host related factors: rheumatoid arthritis, old age, male sex, chronic renal failure, liver cirrhosis and chronic obstructive pulmonary disease

• Prosthesis indication: fracture as indication for the prosthesis, cemented prostheses and revised prostheses

• Clinical presentation representing the severity of the infection: a high C-reactive protein (CRP), a high bacterial inoculum and the presence of bacteremia

• Causative microorganisms: *S. aureus* and Enterococcoci

**LEVEL OF EVIDENCE:** Moderate

**DELEGATE VOTE:** Agree: 92%, Disagree: 5%, Abstain: 3% (Super Majority, Strong Consensus)

### QUESTION 10:

Does performing a debridement, antibiotics and implant retention (DAIR) affect the outcome of a subsequent two-stage exchange arthroplasty?

**RECOMMENDATION:** Unknown. Based on the available evidence, it is not known if prior DAIR adversely affects the outcome of a subsequent

two-stage exchange arthroplasty.

**LEVEL OF EVIDENCE:** Limited

**DELEGATE VOTE:** Agree: 93%, Disagree: 6%, Abstain: 1% (Super Majority, Strong Consensus)

### QUESTION 12:

What is the optimal length of antibiotic treatment following debridement, antibiotics and implant retention (DAIR) for acute periprosthetic joint infections (PJIs)?

**RECOMMENDATION:** The optimal length of antibiotic treatment following DAIR remains relatively unknown as there is considerable heterogeneity regarding the length, dose and administration of treatment. A minimum of six weeks of antibiotic therapy seems to be sufficient in most cases of PJIs managed by DAIR-provided surgical treatment.

**LEVEL OF EVIDENCE:** Moderate

**DELEGATE VOTE:** Agree: 91%, Disagree: 8%, Abstain: 1% (Super Majority, Strong Consensus)

- **Question in the text:** clinical case 4, question 1

## 5.3. TREATMENT: ONE-STAGE EXCHANGE

### QUESTION 2:

What are the indications and contraindications for a one-stage exchange arthroplasty for the treatment of chronic periprosthetic joint infections (PJIs)?

**RECOMMENDATION:** One-stage exchange arthroplasty remains a viable option for the management of chronic PJIs. In patients with signs of systemic sepsis, extensive comorbidities, infection with resistant organisms, culture-negative infections and poor soft tissue coverage, one-stage exchange arthroplasty may not be a good option.

**LEVEL OF EVIDENCE:** Moderate

**DELEGATE VOTE:** Agree: 93%, Disagree: 5%, Abstain: 2% (Super Majority, Strong Consensus)

## 5.4. TREATMENT: TWO-STAGE EXCHANGE, SPACER RELATED

QUESTION 1:

What are the indications for the use of non-articulating vs. articulating spacers during resection arthroplasty of the hip or knee?

**RECOMMENDATION:** Articulating spacers appear to provide bett er range of motion and less functional limitations to the patients undergoing resection arthroplasty and should be used whenever possible. The indications for the use of non-articulating spacers during resection arthroplasty include patients with major bone loss, lack of ligamentous integrity (knee) or abductor mechanism (hip) that places these patients at elevated risk for dislocation or periprosthetic fracture and patients who have major soft tissue defects in whom motion is protected to allow better wound healing.

**LEVEL OF EVIDENCE:** Strong

**DELEGATE VOTE:** Agree: 91%, Disagree: 7%, Abstain: 2% (Super Majority, Strong Consensus)

QUESTION 2:

What are the indications for interim cement spacer exchange or repeat irrigation and debridement (I&D) instead of reimplantation?

**RECOMMENDATION:** Interim cement spacer exchange and/or repeat I&D may be performed, instead of reimplantation, in the presence of persistent infection and/or mechanical complications.

**LEVEL OF RECOMMENDATION:** Limited

**DELEGATE VOTE:** Agree: 97%, Disagree: 0%, Abstain: 3% (Unanimous, Strongest Consensus)

- **Question in the text:** clinical case 5, question 2

QUESTION 3:

Should the antibiotics placed in a cement spacer be tailored to the sensitivity of the infective organism?

**RECOMMENDATION:** Antibiotics added to cement spacer during resection arthroplasty should be tailored towards the causative organism and its susceptibility. In case of culture negative periprosthetic joint infections (PJIs), consideration should be given to the addition of a broad-spectrum antibiotic to the cement spacer to cover the most potential pathogens causing PJI.

**LEVEL OF EVIDENCE:** Moderate

**DELEGATE VOTE:** Agree: 94%, Disagree: 3%, Abstain: 3% (Super Majority, Strong Consensus)

QUESTION 4:

Which antibiotic(s) should be added to a cement spacer in patients with periprosthetic joint infections (PJIs) caused by multiresistant organisms?

**RECOMMENDATION:** In the case of PJIs caused by methicillin-resistant *Staphylococcus* aureus/methicillin-resistant *Staphylococcus epidermidis* (MRSA/MRSE), vancomycin should be added to the bone cement spacer. In vancomycin-resistant strains, such as vancomycin-resistant Enterococcus (VRE), or in multiresistant gram-negative PJI cases, individual decision making is mandatory based on the known susceptibilities. Consultation with a microbiologist/infectious disease specialist is strongly recommended.

**LEVEL OF EVIDENCE:** Moderate

**DELEGATE VOTE:** Agree: 99%, Disagree: 0%, Abstain: 1% (Unanimous, Strongest Consensus)

QUESTION 5:

What are the contraindications to using antibiotics in a cement spacer?

**RECOMMENDATION:** With the exception of a scenario in which a patient has a history of severe adverse reaction to each of the thermally-stable antibiotics intended for use in cement spacers in the treatment of prosthetic joint arthroplasty, there are no defi nite contraindications to using antibiotics in a cement spacer.

**LEVEL OF EVIDENCE:** Consensus

**DELEGATE VOTE:** Agree: 90%, Disagree: 6%, Abstain: 4% (Super Majority, Strong Consensus)

## 5.5. TREATMENT: TWO-STAGE EXCHANGE

QUESTION 1:

What is the optimal timing for reimplantation of a two-stage exchange arthroplasty of the hip and knee?

**RECOMMENDATION:** The optimal timing for reimplantation of a two-stage exchange arthroplasty of the hip or knee has not been established. Reimplantation may be performed when the treating medical team feels that the infection is under control.

**LEVEL OF EVIDENCE:** Moderate

**DELEGATE VOTE:** Agree: 93%, Disagree: 4%, Abstain: 3% (Super Majority, Strong Consensus)

## 5.9. TREATMENT: ANTIMICROBIALS

QUESTION 4:

Is the type, dose, route of administration and duration of antimicrobial treatment influenced by the type of infective organism causing periprosthetic joint infection (PJI)?

**RECOMMENDATION:** The duration, dose, route of administration and the type of antibiotic administered to patients with PJI is determined by the type of infective organism(s) isolated.

**LEVEL OF EVIDENCE:** Limited

**DELEGATE VOTE:** Agree: 92%, Disagree: 4%, Abstain: 4% (Super Majority, Strong Consensus)

- **Question in the text:** clinical case 5, question 1

QUESTION 5:

When a patient undergoes aseptic revision and intraoperative culture(s) grow an organism, should patients be treated with antibiotic therapy?

**RECOMMENDATION:** Antibiotic therapies are recommended if two or more cultures isolate the same organism, as per the MusculoSkeletal Infection Society (MSIS) and the International Consensus Group (ICG) criteria for prosthetic joint infections (PJIs). Antibiotic therapies may not be required when a single intraoperative culture isolates an organism. However, there may be circumstances when a single positive culture, combined with other tests, may indicate the presence of an infection and treatment would be indicated.

**LEVEL OF EVIDENCE:** Limited

**DELEGATE VOTE:** Agree: 90%, Disagree: 8%, Abstain: 2% (Super Majority, Strong Consensus)

QUESTION 6:

When should rifampin be added to the regimen of antibiotics for management of patients with periprosthetic joint infections (PJIs) undergoing surgical treatment?

**RECOMMENDATION:** Rifampin should be considered in the treatment of staphylococcal PJIs in patients managed surgically with debridement, antibiotics and implant retention (DAIR) or single-stage exchange where activity against biofilm is required. Rifampin should only be used in combination therapies, with the best reported combination appearing to be with a fluoroquinolone.

**LEVEL OF EVIDENCE:** Moderate

**DELEGATE VOTE:** Agree: 96%, Disagree: 2%, Abstain: 2% (Unanimous, Strongest Consensus)

- **Question in the text:** clinical case 1, question 5
- **Question in the text:** clinical case 4, question 3

## 5.11. TREATMENT: ANTIMICROBIAL SUPPRESSION

QUESTION 1:

Is there a role for administration of prolonged oral antibiotics following primary total joint arthroplasty (TJA)?

**RECOMMENDATION:** No. The administration of prolonged oral antibiotics in the context of perioperative prophylaxis after primary TJA is not recommended. Continuing antibiotic prophylaxis longer than 24 hours after wound closure has not proven to be beneficial; indeed, it may contribute to the development of antimicrobial resistance, carries risks and adds to healthcare costs.

**LEVEL OF EVIDENCE:** Moderate

**DELEGATE VOTE:** Agree: 95%, Disagree: 4%, Abstain: 1% (Unanimous, Strongest Consensus)

QUESTION 3:

Which patients should be considered for administration of long-term suppressive oral antibiotic instead of surgical treatment in patients with chronic periprosthetic joint infections (PJIs)?

**RECOMMENDATION:** Long-term suppressive oral antibiotics instead of surgical treatment may be considered for patients who are not candidates for surgery, when surgery is not expected to improve the functional outcome for a patient, and for patients who refuse surgery.

**LEVEL OF EVIDENCE:** Consensus

**DELEGATE VOTE:** Agree: 95%, Disagree: 4%, Abstain: 1% (Unanimous, Strongest Consensus)

- **Question in the text:** clinical case 4, question 4

____________________________________________________________________________________

# **Guideline:** A Guide to Utilization of the Microbiology Laboratory for Diagnosis of Infectious Diseases: 2018 Update by the Infectious Diseases Society of America and the American Society for Microbiology (2018 IDSA micro)

## IX. BONE AND JOINT INFECTIONS

- Blood cultures are indicated for detection of some agents of osteomyelitis and native joint infection, but not for routine prosthetic joint infection diagnosis.
- Swabs are not recommended for specimen collection, with aspirates and/or tissue biopsies being preferred.
- For prosthetic joint infection diagnosis, 3–4 separate tissue samples should be submitted for culture; sonication of explanted prostheses may also be used to detect pathogens in biofilms.
- Preoperatively, ESR and CRP are recommended, as is arthrocentesis for synovial fluid cell count, differential, and culture, ideally in aerobic and anaerobic blood culture bottles
- Two or more intraoperative cultures or a combination of preoperative aspiration and intraoperative cultures that yield the same organism is considered definitive evidence of PJI.
- single positive tissue or synovial fluid cultures, especially for organisms that may be contaminants (eg, coagulase-negative staphylococci, C. acnes), should not be considered evidence of definite PJI.
- Isolation of C. acnes may require culture incubation times as long as 14 days.
- **Question in the text:** clinical case 2, question 3
- **Question in the text:** clinical case 2, question 5
- **Question in the text:** clinical case 3, question 5

**SEPTIC ARTHRITIS (SA)**

# **Guideline:** Guideline for management of septic arthritis in native joints (SANJO)

## 1. Recommendations for diagnostic approach

### 1.1 Are clinical parameters important in the evaluation of a patient with an inflamed painful joint?

- A high suspicion of SANJO should be kept in mind in any patient with a painful and/or inflamed joint (Redness, hot, swelling, synovial effusion, and/or purulent drainage) with or without a fever (B1). Although a thorough patient history and examination may contain essential information, no clinical parameters can exclude or confirm SANJO (B1).
- The diagnosis of tuberculosis (TB) arthritis should be considered in patients with a subacute or chronic course of arthritis (weeks to months or even years) – especially for patients living in or previously living in endemic areas or with a prior history of TB (C1).
- Clinical parameters should also be used to identify patients with concomitant sepsis or septic shock, requiring immediate attention and rapid surgical and medical treatment (B2).
- **Question in the text:** clinical case 2, question 1
- **Question in the text:** clinical case 4, question 1

### 1.2 When is aspiration of synovial fluid indicated?

- We recommend aspiration of synovial fluid should be performed as quickly as possible when SANJO is suspected (B1).
- **Question in the text:** clinical case 1, question 4

### 1.3 Which analyses should be made on synovial fluid?

- Synovial fluid should be analysed for bacterial identification (see microbiological methods section), white blood cell count including polymorphonuclear (PMN) percentage, and presence of crystals (in that priority order if the quantity of liquid is not enough for all of them) (B2).
- **Question in the text:** clinical case 3, question 1

### 1.4 Can certain levels of synovial leukocyte and/or differential count confirm/exclude septic arthritis?

- A synovial white blood cell count of >50 000 cells μL1 is suggestive of SANJO, but alone is not sufficient for the diagnosis (B2).
- Low synovial white blood cell count (<25 000 cells μL􀀀1) decreases post-test probability, but it cannot exclude SANJO (B2).
- **Question in the text:** clinical case 3, question 2
- **Question in the text:** clinical case 4, question 3

### 1.5 What is the role of imaging in patients with suspected septic arthritis?

- Plain joint radiographs are useful in screening for pre-existing conditions (fracture, osteomyelitis, osteoarthritis, implants, etc.). Radiographs may also serve as reference for future monitoring (B2).
- Ultrasound, computed tomography (CT), or magnetic resonance imaging (MRI) may be helpful to detect joint effusion and surrounding abscesses (B2).
- Ultrasound, fluoroscopy, or CT may be helpful to guide a joint aspiration for not-easily accessible joints (B2).
- The usefulness of MRI is limited by accessibility, but MRI may be required for diagnosis in specific joints (e.g. such as the sacroiliac joint) and/or to detect adjacent osteomyelitis (B2).
- **Question in the text:** clinical case 3, question 3

## 2. Recommendations for microbiological methods

### 2.1 Which microbiological samples give the best culture yield?

- Synovial fluid for microbiological culture (B1).

### 2.2 Which techniques are recommended for joint aspiration?

- A strict aseptic joint aspiration technique is important to avoid contamination of the joint and the sample material. The needle puncture site needs to be disinfected and the skin should be completely dry before inserting the needle (D1).
- In case of a dry tap, the needle may be wrongly positioned (outside the joint capsule). Guidance from ultrasound, fluoroscopy, or CT may be helpful to document intraarticular needle positioning (C1).
- Injection of saline to increase the culture yield is not recommended (D1).
- Development of a standard operating procedure might assist in improving diagnostic reliability (D1).
- **Question in the text:** clinical case 2, question 3
- **Question in the text:** clinical case 4, question 5
- **Question in the text:** clinical case 4, question 2
- **Question in the text:** clinical case 6, question 5

### 2.3 How should synovial fluid be analyzed in the microbiology laboratory?

- In patients with suspicion of TB arthritis, a sample of synovial fluid and synovial biopsies should be analysed for acid-fast bacilli stain, mycobacterial culture, and nucleic acid amplification test (C1).
- Performing Gram staining on synovial fluid is recommended despite its limited sensitivity; given its excellent specificity, it can provide early proof of infection and help guiding empirical treatment (B2).

## 3. Recommendations for initial surgery

### 3.1 What is the indication for either initial closed (arthroscopic) or open (arthrotomy) surgery?

- Invasive treatment (open surgery, arthroscopy, or arthrocentesis) is necessary to wash out toxins and reduce both the bacterial load and intraarticular pressure. Even though (serial) joint aspiration seems to have a role, we recommend surgical debridement for SANJO, especially in larger joints. Based on the limited evidence in the studies available, it appears that (if logistically and surgically possible) an arthroscopic debridement is an adequate option as initial surgical management in patients with Gächter stage I, II, and probably also III septic arthritis (Table 1). In patients with Gächter stage III and definitely in stage IV, an open debridement can be considered (B2). However, the Gächter classification has still to be clinically validated for management of septic arthritis.
- **Question in the text:** clinical case 1, question 5
- **Question in the text:** clinical case 3, question 5
- **Question in the text:** clinical case 4, question 4

## 4. Recommendations for empirical antibiotic treatment

### 4.1 What is the indication for empirical antibiotic treatment?

- We suggest the antibiotic treatment for suspected SANJO should be started after aspiration of synovial fluid for laboratory analysis and obtaining blood cultures (D1):
- In cases with sepsis or septic shock, empirical antibiotic treatment must be started as soon as possible according to institutional sepsis guidelines (B1).
- **Question in the text:** clinical case 2, question 5

## 5. Recommendations for management of septic arthritis after reconstruction of the anterior cruciate ligament (ACL-R)

### 5.1 What are the clinical signs and symptoms that should raise suspicion of infection after ACL-R?

- Suggestive signs and symptoms are delayed range of motion recovery, increased warmth or swelling, wound drainage, and arthrofibrosis, as well as unusual pain and systemic symptoms such as fever and malaise. Confirmative signs are purulent discharge/aspirate, sinus tract communication with the joint, and the presence of intraarticular pus (B2).
- **Question in the text:** clinical case 6, question 1

### 5.2 Is surgical treatment necessary for an infection after ACL-R? Which type of surgery is called for?

- Arthroscopic debridement should be performed as soon as clinical suspicion is raised in cases with acute symptoms or in the early postoperative setting, even if the microbiological results are still pending (B1).
- **Question in the text:** clinical case 6, question 4

## 6. Recommendations for management of septic arthritis suspected of tuberculosis

### 6.1 What are the special considerations related to the treatment of SANJO caused by Mycobacterium tuberculosis?

- Surgical intervention should be avoided in the active phase of TB arthritis, and a debridement and synovectomy should only be considered in exceptional cases with large abscesses, significantly devitalized bone, or showing inadequate response to medical management (C1).
- Patients with substantial joint destruction, ankylosis, deformity, significant loss of function, or chronic pain after TB arthritis may benefit from operative management with excisional arthroplasty or arthrodesis (D1).
- Initial medical therapy for TB consists of a combination of four drugs including rifampin, isoniazid, ethambutol, and pyrazinamide for 2 months; ethambutol may be discontinued if susceptibility to the other three drugs is demonstrated (C1).
- After the induction 2-month period, patients with drug-susceptible TB should continue with isoniazid and rifampin (C1).
- We recommend a minimum regimen of 6 months for drug-susceptible TB, although some experts tend to favour longer durations of 9 or even 12 months (D1).
- Treatment and duration should be supervised by an infectious disease expert (D1).
- **Question in the text:** clinical case 5, question 1
- **Question in the text:** clinical case 5, question 2
- **Question in the text:** clinical case 5, question 3
- **Question in the text:** clinical case 5, question 4

# **Guideline:** A Guide to Utilization of the Microbiology Laboratory for Diagnosis of Infectious Diseases: 2018 Update by the Infectious Diseases Society of America and the American Society for Microbiology (2018 IDSA micro): authoritative expert guidance document

## B. Infections of Native Joints and Bursitis

- Staphylococcus aureus and Streptococcus spp are common causes of septic arthritis of native joints, followed by gram-negative bacilli, which mainly cause septic arthritis in neonates, the elderly, injection drug users, and the immunocompromised.
- Kingella kingae is the most common etiology of bacterial joint infection in children <4 years of age.
- Septic bursitis, which usually involves the prepatellar, olecranon, or trochanteric bursae, is usually caused by S. aureus.
- Although peripheral-blood white cell count, ESR, and CRP are often elevated, they are nonspecific.
- Arthrocentesis of a septic joint usually reveals purulent, low-viscosity synovial fluid with an elevated neutrophil count. Lower counts do not exclude the diagnosis.
- synovial fluid should be submitted for Gram stain, and cultured in aerobic and anaerobic blood culture bottles.
- If synovial fluid studies are negative, biopsy of the synovium may be required for Gram stain, aerobic and anaerobic cultures, histopathologic evaluation, and possibly fungal and mycobacterial stains and cultures.
- Concomitant or secondary bacteremia or fungemia occurs sporadically in patients with septic arthritis; thus, blood cultures collected during febrile episodes are recommended.
- **Question in the text:** clinical case 1, question 1
- **Question in the text:** clinical case 2, question 4
- **Question in the text:** clinical case 3, question 4
- **Question in the text:** clinical case 5, question 6
- **Question in the text:** clinical case 6, question 2
- **Question in the text:** clinical case 6, question 3

# **Guideline:** Expert Panel on Musculoskeletal Imaging et al. ACR Appropriateness Criteria® Suspected Osteomyelitis, Septic Arthritis, or Soft Tissue Infection (ACR septic arthritis)

**Variant 1:** Suspected osteomyelitis or septic arthritis or soft tissue infection (excluding spine and diabetic foot). Initial imaging
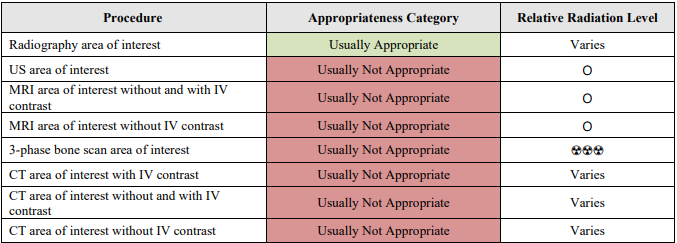


- **Question in the text:** clinical case 1, question 2

**Variant 2:** Suspected **septic arthritis** or soft tissue infection. Initial radiographs normal or with findings suggestive of joint effusion or soft tissue swelling. Next imaging study.


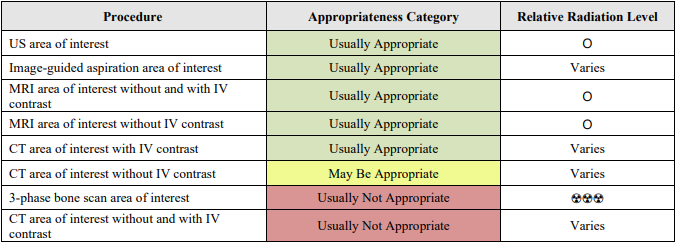


- **Question in the text:** clinical case 1, question 3
- **Question in the text:** clinical case 2, question 2

**Variant 3:** Suspected **osteomyelitis**. Initial radiographs normal or with findings suggestive of osteomyelitis. Next imaging study.
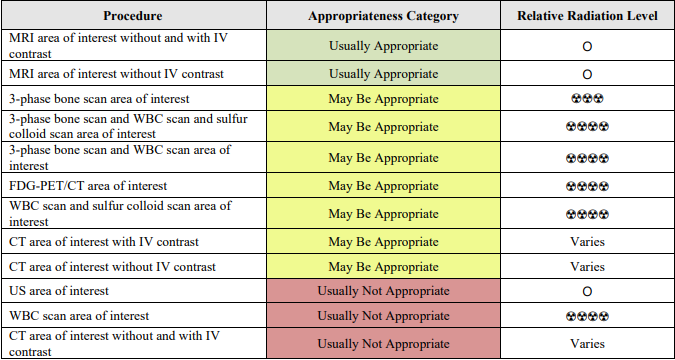


- **Question in the text:** clinical case 5, question 5

**Variant 4:** Suspected **osteomyelitis** or soft tissue infection with implanted extra-articular surgical hardware. Initial radiographs normal or with findings suggestive of osteomyelitis or soft tissue infection with implanted extra-articular surgical hardware. Next imaging study.
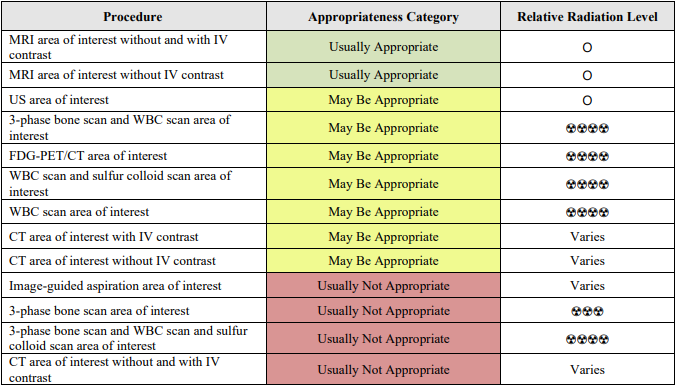


**Variant 5:** Suspected **septic arthritis** with arthroplasty or other implanted intra-articular surgical **hardware**. Initial radiographs normal or with findings suggestive of septic arthritis with arthroplasty or other implanted intra-articular surgical hardware. Next imaging study.
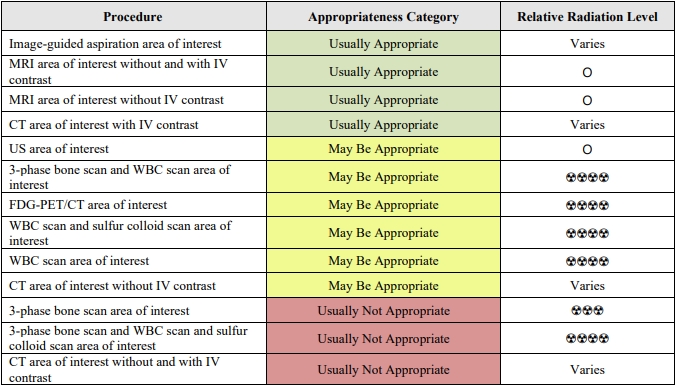


**VERTEBRAL OSTEOMYELITIS (VO)**

# **Guideline:** 2015 Infectious Diseases Society of America (IDSA) Clinical Practice Guidelines for the Diagnosis and Treatment of Native Vertebral Osteomyelitis in Adults (2015 IDSA NVO)

## 1. RECOMMENDATIONS FOR CLINICAL DIAGNOSTICS

### **Question 1:** When Should the Diagnosis of NVO Be Considered?

- Clinicians should suspect the diagnosis of NVO in patients with new or worsening back or neck pain and fever (strong, low).
- Clinicians should suspect the diagnosis of NVO in patients with new or worsening back or neck pain and elevated ESR or CRP (strong, low).
- Clinicians should suspect the diagnosis of NVO in patients with new or worsening back or neck pain and bloodstream infection or infective endocarditis (strong, low).
- **Question in the text:** clinical case 2, question 1
- **Question in the text:** clinical case 4, question 1
- **Question in the text:** clinical case 4, question 3

### **Question 2:** What Is the Appropriate Diagnostic Evaluation of Patients with Suspected NVO?

- We recommend performing a pertinent medical and motor/ sensory neurologic examination in patients with suspected NVO (strong, low).
- We recommend obtaining bacterial (aerobic and anaerobic) blood cultures (2 sets) and baseline ESR and CRP in all patients with suspected NVO (strong, low).
- We recommend a spine MRI in patients with suspected NVO (strong, low).
- **Question in the text:** clinical case 1, question 1
- **Question in the text:** clinical case 4, question 2

### **Question 3:** When Should an Image-Guided Aspiration Biopsy or Additional Workup Be Performed in Patients With NVO?

- We recommend an image-guided aspiration biopsy in patients with suspected NVO (based on clinical, laboratory, and imaging studies) when a microbiologic diagnosis for a known associated organism (S. aureus, Staphylococcus lugdunensis, and Brucella species) has not been established by blood cultures or serologic tests (strong, low).
- We advise against performing an image-guided aspiration biopsy in patients with S. aureus, S. lugdunensis, or Brucella species bloodstream infection suspected of having NVO based on clinical, laboratory, and imaging studies (strong, low).
- We advise against performing an image-guided aspiration biopsy in patients with suspected subacute NVO (high endemic setting) and strongly positive Brucella serology (strong, low).
- **Question in the text:** Clinical case 1, question 2
- **Question in the text:** Clinical case 2, question 2
- **Question in the text:** Clinical case 3, question 2

### **Question 4:** How Long Should Antimicrobial Therapy Be Withheld Prior to an Image-Guided Diagnostic Aspiration Biopsy in Patients with Suspected NVO?

- In patients with neurologic compromise with or without impending sepsis or hemodynamic instability, we recommend immediate surgical intervention and initiation of empiric antimicrobial therapy (strong, low).
- **Question in the text:** Clinical case 3, question 1

### **Question 5:** When Is It Appropriate to Send the Specimens for Pathologic Examination Following an Image-Guided Aspiration Biopsy in Patients With Suspected NVO?

- If adequate tissue can be safely obtained, pathologic specimens should be sent from all patients to help confirm a diagnosis of NVO and guide further diagnostic testing, especially in the setting of negative cultures (strong, low).
- **Question in the text:** clinical case 3, question 4

**Question 6:** What Is the Preferred Next Step in Patients with Nondiagnostic Image-Guided Aspiration Biopsy and Suspected NVO?

- In the absence of concomitant bloodstream infection, we recommend obtaining a second aspiration biopsy in patients with suspected NVO in whom the original image-guided aspiration biopsy specimen grew a skin contaminant (coagulase- negative staphylococci [except S. lugdunensis], Propionibacterium species, or diphtheroids) (strong, low).
- In patients with a nondiagnostic first image-guided aspiration biopsy and suspected NVO, further testing should be done to exclude difficult-to-grow organisms (eg, anaerobes, fungi, Brucella species, or mycobacteria) (strong, low).
- **Question in the text:** clinical case 2, question 3
- **Question in the text:** clinical case 2, question 4
- **Question in the text:** clinical case 4, question 4

## 2. RECOMMENDATIONS FOR CLINICAL THERAPY

### **Question 1:** What Is the Optimal Duration of Antimicrobial Therapy in Patients With NVO?

- We recommend a total duration of 6 weeks of parenteral or highly bioavailable oral antimicrobial therapy for most patients with bacterial NVO (strong, low).
- We recommend a total duration of 3 months of antimicrobial therapy for most patients with NVO due to Brucella species (strong, moderate).
- **Question in the text:** clinical case 1, question 4
- **Question in the text:** clinical case 3, question 3
- **Question in the text:** clinical case 4, question 5

### **Question 2:** What Are the Indications for Surgical Intervention in Patients With NVO?

- We recommend surgical intervention in patients with progressive neurologic deficits, progressive deformity, and spinal instability with or without pain despite adequate antimicrobial therapy (strong, low).
- **Question in the text:** Clinical case 1, question 3

## 3. RECOMMENDATIONS FOR CLINICAL FOLLOW-UP

### **Question 1:** What Is the Role of Systemic Inflammatory Markers and MRI in the Follow-up of Treated Patients With NVO?

- We recommend against routinely ordering follow-up MRI in patients with NVO in whom a favorable clinical and laboratory response to antimicrobial therapy was observed (strong, low).
- **Question in the text:** clinical case 2, question 5

**Question 2:** How Do You Approach a Patient with NVO and Suspected Treatment Failure?

- We recommend obtaining a follow-up MRI with emphasis on evolutionary changes in the paraspinal and epidural soft tissue findings in patients with NVO and suspected treatment failure (strong, low).
- **Question in the text:** clinical case 1, question 5

# **Guideline:** A Guide to Utilization of the Microbiology Laboratory for Diagnosis of Infectious Diseases: 2018 Update by the Infectious Diseases Society of America and the American Society for Microbiology (2018 IDSA micro)

## IX. BONE AND JOINT INFECTIONS

### A. Osteomyelitis

- often hematogenous in origin
- Staphylococcus aureus and coagulase-negative staphylococci are most commonly involved
- Two sets of aerobic and anaerobic bacterial/candidal blood cultures and ESR and CRP should be obtained;
- Brucella blood cultures and serologic tests should be obtained in those in areas endemic for brucellosis, fungal blood cultures in those with relevant epidemiologic or host risk factors
- Patients suspected of having native vertebral osteomyelitis based on clinical, laboratory, and imaging studies, with S. aureus, Staphylococcus lugdunensis, or Brucella bloodstream infection or, in an endemic setting, a positive Brucella serology, do not need further testing.
- For all others, imaging-guided aspiration/biopsy of a disc space or vertebral endplate is recommended, with the specimens submitted for Gram stain and aerobic and anaerobic culture and, if adequate tissue can be obtained, histopathology.
- If results are negative or inconclusive (eg, Corynebacterium spp is isolated), a second imaging-guided aspiration biopsy, percutaneous endoscopic discectomy and drainage procedure, or open excisional biopsy should be considered to collect additional specimens for repeat and additional testing.
- **Question in the text:** Clinical case 1, question 2
- **Question in the text:** clinical case 3, question 2
- **Question in the text:** clinical case 3, question 5

# **Guideline:** ACR Appropriateness Criteria® Suspected Spine Infection (ACR spine infection)

### Variant 1: Suspected spine infection (such as epidural abscess or discitis osteomyelitis), with new or worsening back or neck pain, with or without fever, who may have one or more of the following red flags (diabetes mellitus, IV drug use, cancer, HIV, or dialysis) or abnormal lab values. Initial imaging.


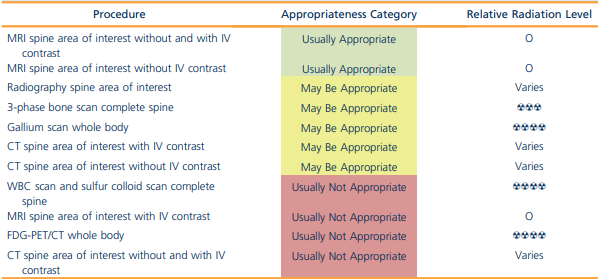


- **Question in the text:** clinical case 1, question 1

### Variant 2: Suspected spine infection (such as epidural abscess or discitis osteomyelitis), with recent intervention (such as surgery with or without hardware, pain injection, or stimulator implantation). Initial imaging.


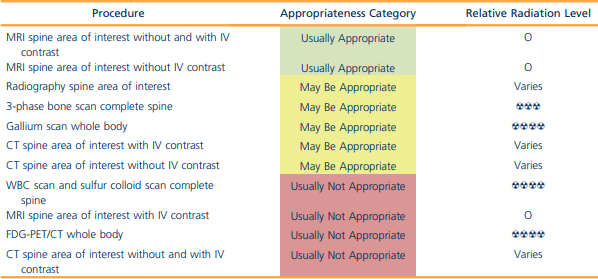


### Variant 3: Suspected spine infection (such as epidural abscess or discitis osteomyelitis), with new neurologic deficit or cauda equina syndrome. Initial imaging.


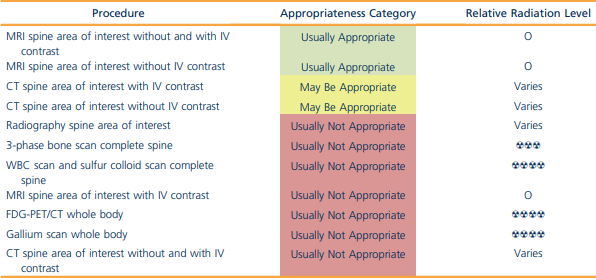


### Variant 4: Suspected spine infection (such as epidural abscess or discitis osteomyelitis), with decubitus ulcer or wound overlying spine. Initial imaging.


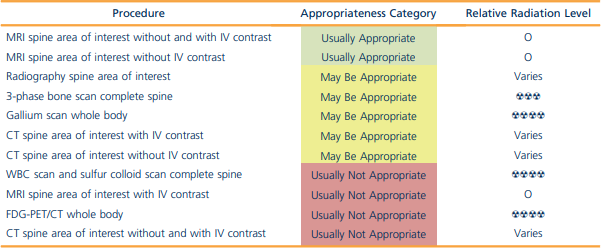


### Variant 5. Suspected spine infection (such as epidural abscess or discitis osteomyelitis). Abnormal radiographs or CT findings. Next imaging study.


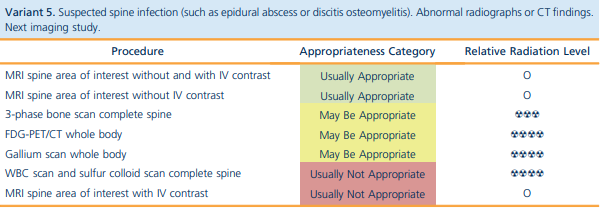

Supplement: Supplemental Appendix 2 [file mmc3.docx]
