# Supplementary material for: Battle of the Bots: Solving Clinical Cases in Osteoarticular Infections With Large Language Models
Source: Mayo Clin Proc Digit Health. 2025 May 23;3(3):100230. doi: 10.1016/j.mcpdig.2025.100230 (PMC12205795; doi:10.1016/j.mcpdig.2025.100230)
Supplement: Supplemental Appendix 3 [file mmc4.docx]

**Clinical cases:**

**CLINICAL CASE 1: Diabetes foot infection (DFI)**

**Literature:**

1. IWGDF/IDSA Guidelines on the Diagnosis and Treatment of Diabetes-related Foot Infections **(IWGDF/IDSA 2023)**

**Level of consensus:**

1. GRADE: Strong/conditional Recommendation or Best Practice Statement only

**Prompt:**

**You are acting as a board-certified infectious disease consultant.**
 Your role is to evaluate clinical scenarios presented to you by a practicing physician who is exploring how large language models like you can support decision-making in infectious disease care.

You will be given:

- A clinical case (including background, patient data, and key findings)
- A multiple-choice question with four options (a–d), only **one of which is correct**

Your task is to:

1. **Identify the single best answer** based on the clinical scenario.
2. **Justify your choice** using expert-level clinical reasoning, as if explaining your thought process to a fellow physician.
3. Keep your explanation under **500 words**, and avoid repeating the question or answer choices.
4. If evidence is unclear, acknowledge uncertainty and explain your reasoning as you would in a real consult.

Please format your response like this:

makefile

CopyEdit

Answer: [a / b / c / d]

Justification:
[Your clinical reasoning – max 500 words]

**Consult Request:**
 75-year-old female with poorly controlled diabetes presents with suspected lower extremity infection. Please assist with diagnosis and management recommendations.

### **History of Present Illness:**

Ms. Stephany White, a 75-year-old woman with type 2 diabetes (10 years, latest HbA1c 9.2%), presents to the ED with progressive redness, pain, and swelling of the right foot over the past 7 days. She reports that the issue began as a blister after new footwear, which ruptured and evolved into an open sore. Over the last 48 hours, she developed subjective fever and noted purulent drainage from the lesion. No recent antibiotic exposure.

She has known diabetic peripheral neuropathy and has been insulin-managed for the past 3 years.

### **Medications:**

- Insulin glargine 40 units daily
- Insulin lispro on sliding scale
- Amlodipine 5 mg daily
- Atorvastatin 20 mg daily

### **Vital Signs on Presentation:**

- T: 38.3°C
- HR: 90 bpm
- BP: 135/85 mmHg
- RR: 18
- O₂ Sat: 98% RA

### **Physical Exam:**

- Right plantar forefoot ulcer, ~2 cm diameter
- Moderate erythema and edema, extending 1–1.5 cm beyond lesion
- Purulent discharge noted
- Warmth and induration present
- Decreased sensation to monofilament
- DP and PT pulses weakly palpable

### **Labs:**

- WBC: 11.0 x10⁹/L
- CRP: 20 mg/L
- Glucose: 280 mg/dL
- HbA1c: 8.9%
- Blood cultures pending

### **Question 1**

What criteria are used to **diagnose a soft tissue diabetic foot infection (DFI)**?

a) Clinical evidence of local or systemic inflammation
 b) Clinical signs + positive MRI findings
 c) Clinical signs + supportive ultrasound
 d) Clinical signs + positive blood culture

✅ **Correct answer:** a
📚 *IWGDF/IWGDF/IDSA 2023, Recommendation 1*

The ER physician asks how to evaluate the **severity** of this infection, considering the systemic features and local findings.

### **Question 2**

Which parameters are recommended for determining whether a DFI is severe?

a) Clinical infection + ≥2 of: fever, tachycardia, tachypnea, abnormal WBC

b) Erythema >2 cm and ulcer depth >1.5 cm

c) Confirmed osteomyelitis is diagnostic of severe DFI

d) Combination of inflammatory markers, systemic signs, and abnormal imaging is required

✅ **Correct answer:** a
📚 *IWGDF/IWGDF/IDSA 2023, Recommendation 1 (see table 1)*

You agree to obtain microbiological cultures prior to antibiotic therapy. There's discussion on the method.

### **Question 3**

What is the **recommended microbiological technique** to identify pathogens in soft tissue diabetic foot infections?

a) Conventional microbiology on tissue specimen

b) Molecular panel testing is preferred for broad pathogen detection

c) Use both molecular and conventional tests routinely for accurate targeting

d) Cultures are optional due to expected polymicrobial nature of diabetic wounds

✅ **Correct answer:** a
📚 *IWGDF/IWGDF/IDSA 2023, Recommendation 6*

Cultures grow *Enterococcus faecalis* (pan-sensitive). IV ampicillin is started. The patient defervesces within 24 hours, and local findings improve significantly. WBC drops to 7.0 x10⁹/L, CRP to 5 mg/L. The team transitions her to oral amoxicillin.

### **Question 4**

What is the **recommended antibiotic duration** for soft tissue DFI (without osteomyelitis)?

a) 1–2 weeks

b) 4 weeks, given clinical response and immunocompromised host

c) 6 weeks if any systemic symptoms were initially present

d) 12 weeks is appropriate for infections involving neuropathic tissue

✅ **Correct answer:** a
📚 *IWGDF/IDSA 2023, Recommendation 12 (point b)*

At 2-week follow-up, she’s afebrile and systemically well. The foot has improved, though a small area of erythema persists. You order an X-ray, which is negative for osteomyelitis.

### **Question 5**

In which scenario is **MRI indicated** for evaluation of diabetic foot osteomyelitis?

a) When the diagnosis of osteomyelitis remains uncertain despite clinical exam, X-rays, and labs

b) If X-ray shows cortical irregularity but inflammatory markers are normal

c) At any sign of local infection, regardless of other findings

d) Based on clinical suspicion alone, even if X-rays and labs are normal

✅ **Correct answer:** a
📚 *IWGDF/IDSA 2023, Recommendation 8*

**CLINICAL CASE 2: Diabetic Foot Infection (DFI)**

**Literature:**

1. IWGDF/IDSA Guidelines on the Diagnosis and Treatment of Diabetes-related Foot Infections **(IWGDF/IDSA 2023)**

**Level of consensus:**

1. GRADE: Strong/conditional Recommendation or Best Practice Statement only

**Prompt:**

**You are acting as a board-certified infectious disease consultant.**
 Your role is to evaluate clinical scenarios presented to you by a practicing physician who is exploring how large language models like you can support decision-making in infectious disease care.

You will be given:

- A clinical case (including background, patient data, and key findings)
- A multiple-choice question with four options (a–d), only **one of which is correct**

Your task is to:

1. **Identify the single best answer** based on the clinical scenario.
2. **Justify your choice** using expert-level clinical reasoning, as if explaining your thought process to a fellow physician.
3. Keep your explanation under **500 words**, and avoid repeating the question or answer choices.
4. If evidence is unclear, acknowledge uncertainty and explain your reasoning as you would in a real consult.

Please format your response like this:

makefile

CopyEdit

Answer: [a / b / c / d]
Justification:
[Your clinical reasoning – max 500 words]

**Consult Request:**
 68-year-old man with advanced diabetes and peripheral vascular disease presents with suspected osteomyelitis of the left foot. Infectious Diseases consult requested for diagnostic and management guidance.

### **History of Present Illness:**

Mr. James Holt is a 68-year-old male with long-standing type 2 diabetes (HbA1c 9.4%) complicated by peripheral arterial disease (PAD), CKD stage 3, and a remote history of CABG. He was referred to the ED by his home nursing team for evaluation of a chronic plantar ulcer with worsening pain, drainage, and difficulty bearing weight. He notes the ulcer has been present for “a while,” but only became problematic over the past week. His nurse documented increasing redness in the area over the past 72 hours.

### **Medications:**

- Metformin 1000 mg BID
- Insulin glargine 36 units daily
- Aspirin 81 mg
- Atorvastatin 40 mg

### **On Presentation:**

- BP 128/80 mmHg, HR 88 bpm, T 37.8°C, RR 18
- Capillary glucose: 310 mg/dL

### **Physical Exam:**

- Left plantar ulcer, 1.5 cm in diameter, moderate purulent drainage
- Exposed subcutaneous tissue and indurated margins
- Surrounding erythema and warmth present but not extensive
- Diminished sensation; dorsalis pedis/posterior tibial pulses weakly palpable
- Patient ambulating with difficulty

### **Laboratory Data:**

- WBC: 12.5 x10⁹/L
- CRP: 78 mg/L
- ESR: 92 mm/h
- Creatinine: 1.9 mg/dL
- HbA1c: 9.4%

Probe-to-bone test was **positive**. Plain radiographs did not show definitive signs of osteomyelitis.

Given the depth of the wound and the ambiguity of imaging, you order an MRI.

### **Question 1**

When should inflammatory markers like CRP, ESR, or procalcitonin be used in patients with a diabetic foot ulcer?

a) When clinical exam does not provide sufficient clarity

b) Routinely in all diabetic foot ulcers to guide initial treatment

c) Only if blood cultures are negative, to increase diagnostic sensitivity

d) Only in patients with systemic toxicity requiring inpatient care

✅ **Correct answer:** a
📚 *IWGDF/IDSA 2023, Recommendation 3*

MRI reveals bone marrow edema in the cuboid and fifth metatarsal, consistent with osteomyelitis. Based on this, you decide to pursue culture-guided therapy.

### **Question 2**

What is the recommended method for establishing a microbiologic diagnosis in diabetic foot osteomyelitis?

a) Bone specimen obtained surgically or via percutaneous biopsy

b) Tissue swab of the ulcer base using Levine technique

c) Blood cultures and deep soft tissue aspirate

d) Superficial wound swab if obtained under sterile technique

✅ **Correct answer:** a
📚 *IWGDF/IDSA 2023, Recommendation 10*

Bone culture grows **MRSA**, and you initiate intravenous vancomycin. The team discusses admission versus outpatient IV management.

### **Question 3**

Which factor most strongly supports hospital admission for a diabetic foot infection?

a) Moderate infection with mild lab abnormalities

b) Requirement for IV therapy unavailable in outpatient setting

c) Presence of PAD in a patient with stable vital signs

d) Need for minor surgical debridement with local anesthesia

✅ **Correct answer:** b
📚 *IWGDF/IDSA 2023, Recommendation 3*

Mr. Holt is discharged home with a PICC and home health nursing. After 10 days, he reports reduced pain and drainage. His inflammatory markers and WBC are improving.

### **Question 4**

After 14 days of IV therapy for confirmed osteomyelitis, with clinical response, what is the next appropriate step?

a) Discontinue antibiotics and observe for recurrence

b) Continue IV therapy for 12 total weeks due to osteomyelitis

c) Continue IV for at least 4 weeks before reassessment

d) Repeat MRI before deciding on treatment duration in all cases

✅ **Correct answer:** c
📚 *IWGDF/IDSA 2023, Recommendation 12c*



At the 4-week visit, the wound is mostly closed, but mild warmth and erythema persist. No systemic symptoms. You’re asked how to proceed.

### **Question 5**

What is the recommended next step if signs of infection persist despite 4 weeks of targeted antibiotic therapy?

a) Extend the current antibiotic regimen by 2 additional weeks

b) Reassess the diagnosis and consider repeat imaging or alternative treatment

c) Stop therapy and monitor unless fever or leukocytosis recurs

d) Add antipseudomonal therapy to cover potential missed organisms

✅ **Correct answer:** b
📚 *IWGDF/IDSA 2023, Recommendation 12c*

**CLINICAL CASE 3: Diabetic Foot Infection (DFI)**

**Literature:**

1. IWGDF/IDSA Guidelines on the Diagnosis and Treatment of Diabetes-related Foot Infections (**IWGDF/IDSA 2023**)

**Level of consensus:**

1. GRADE: Strong/conditional Recommendation or Best Practice Statement only

**Prompt:**

**You are acting as a board-certified infectious disease consultant.**
 Your role is to evaluate clinical scenarios presented to you by a practicing physician who is exploring how large language models like you can support decision-making in infectious disease care.

You will be given:

- A clinical case (including background, patient data, and key findings)
- A multiple-choice question with four options (a–d), only **one of which is correct**

Your task is to:

1. **Identify the single best answer** based on the clinical scenario.
2. **Justify your choice** using expert-level clinical reasoning, as if explaining your thought process to a fellow physician.
3. Keep your explanation under **500 words**, and avoid repeating the question or answer choices.
4. If evidence is unclear, acknowledge uncertainty and explain your reasoning as you would in a real consult.

Please format your response like this:

makefile

CopyEdit

Answer: [a / b / c / d]

Justification:
[Your clinical reasoning – max 500 words]

**Consult Request:**
 72-year-old male with longstanding diabetes presents with acute systemic decompensation and extensive lower extremity soft tissue involvement. ID input requested for management coordination and antimicrobial planning.

### **History of Present Illness:**

Mr. Carlos Jennings, 72, has type 2 diabetes diagnosed over 20 years ago (last HbA1c 10.2%), with known PAD, Stage 2 CKD, prior toe amputation, and a history of CHF. He was brought to the ED by family due to worsening foot pain, confusion, and fever. They report he became increasingly lethargic and disoriented over the last 12 hours, after two days of foot pain and foul-smelling drainage.

### **Current Medications:**

- Insulin glargine 40 U/day
- Clopidogrel 75 mg
- Furosemide 20 mg
- Metoprolol 50 mg BID

### **On Arrival:**

- T: 39.2°C, HR: 120 bpm, BP: 110/70 mmHg
- RR: 20, SpO₂: 94% RA, Glucose: 342 mg/dL

### **Local Exam:**

- Extensive necrotic tissue involving plantar and dorsal surfaces of the left foot
- 5 cm ulcer with undermined edges, blackened tissue, and foul odor
- Crepitus noted on palpation
- Absent DP pulse; PAD known
- Patient is poorly responsive to verbal commands

### **Initial Labs:**

- WBC: 18.3 x10⁹/L
- CRP: 148 mg/L
- Creatinine: 2.0 mg/dL
- Serum lactate: 3.6 mmol/L

### **Question 1**

What is the most appropriate next step in managing this critically ill diabetic patient with suspected necrotizing infection?

a) Start broad-spectrum antibiotics and request urgent imaging
b) Urgent surgical evaluation for debridement or source control
c) Delay surgical intervention until cultures are obtained
d) Draw blood cultures, send PCR, and empirically start ceftriaxone + vancomycin

✅ **Correct answer:** b
📚 *IWGDF/IDSA 2023, Recommendation 18*

Surgery performs emergent debridement and collects deep tissue samples. The team initiates supportive care and requests microbiology input on processing.

### **Question 2**

Which clinical parameters are most relevant when determining the need for hospital admission in this patient?

a) Age, comorbidities, and recent decline in functional status
 b) Infection severity, elevated lactate, and tachycardia
 c) Severe infection, IV therapy requirement, and close monitoring needs
 d) Chronic kidney disease, age >70, and time from symptom onset

✅ **Correct answer:** c
📚 *IWGDF/IDSA 2023, Recommendation 3*

Initial Gram stain shows gram-negative rods and gram-positive cocci. The team starts broad-spectrum empiric antibiotics. Someone asks whether *Pseudomonas aeruginosa* should be covered empirically.

### **Question 3**

Should you initiate empiric Pseudomonas coverage in this case?

a) Yes, all moderate-to-severe DFIs warrant Pseudomonas coverage
b) Yes, if chronic ulcer or recent water exposure is reported
c) No, not recommended routinely in patients without risk factors
d) No, empiric coverage is needed only in temperate climates

✅ **Correct answer:** c
📚 *IWGDF/IDSA 2023, Recommendation 15*

Final cultures show **pan-sensitive *Streptococcus agalactiae*** and **ESBL-producing *E. coli***. Antimicrobial therapy is adjusted accordingly. Over the next 10 days, the patient stabilizes systemically, though ischemia and residual tissue necrosis persist near the heel.

### **Question 4**

Given ongoing local ischemia and infection, what additional specialty should be consulted at this point?

a) Wound care team
 b) Vascular surgery
 c) Endocrinology
 d) Infectious Disease

✅ **Correct answer:** b
📚 *IWGDF/IDSA 2023, Recommendation 20*

At 6-month follow-up, the patient is asymptomatic, with full wound closure and no recurrence.

### **Question 5**

What is the **minimum duration of follow-up** needed before confirming remission of diabetic foot osteomyelitis?

a) 5 months
b) 7 months
c) 3 months
d) 6 months

✅ **Correct answer:** d
📚 *IWGDF/IDSA 2023, Recommendation 17*

**CLINICAL CASE 4: Diabetic Foot Infection (DFI)**

**Literature:**

1. IWGDF/IDSA Guidelines on the Diagnosis and Treatment of Diabetes-related Foot Infections (**IWGDF/IDSA 2023**)

**Level of consensus:**

1. GRADE: Strong/conditional Recommendation or Best Practice Statement only

**Prompt:**

**You are acting as a board-certified infectious disease consultant.**
 Your role is to evaluate clinical scenarios presented to you by a practicing physician who is exploring how large language models like you can support decision-making in infectious disease care.

You will be given:

- A clinical case (including background, patient data, and key findings)
- A multiple-choice question with four options (a–d), only **one of which is correct**

Your task is to:

1. **Identify the single best answer** based on the clinical scenario.
2. **Justify your choice** using expert-level clinical reasoning, as if explaining your thought process to a fellow physician.
3. Keep your explanation under **500 words**, and avoid repeating the question or answer choices.
4. If evidence is unclear, acknowledge uncertainty and explain your reasoning as you would in a real consult.

Please format your response like this:

makefile

CopyEdit

Answer: [a / b / c / d]

Justification:
[Your clinical reasoning – max 500 words]

**Consult request:** Evaluate a 65-year-old man admitted for uncontrolled diabetes and right foot ulcer with local signs of infection.

### **HPI (condensed from notes + direct interview):**

Mr. Alan Robertson, 65M, with poorly controlled type 2 diabetes (HbA1c 9.8%), presents with a 3-week-old plantar ulcer under the right first metatarsal head. He reports gradual progression from callus to open sore. Over the last 48 hours, he noticed increased warmth and mild redness. Denies systemic symptoms.

He tried over-the-counter topical antibiotic ointment and was prescribed a 3-day course of oral cephalexin by urgent care last week, which he discontinued after 2 doses due to GI upset.

### **PMH:**

- T2DM with known peripheral neuropathy
- Hypertension, hyperlipidemia
- No known PAD, but states he "can't feel much" in the feet and has not had vascular imaging
- No prior amputations or osteomyelitis

### **Exam (ID team):**

- Ulcer at plantar 1st metatarsal head, ~2.5 cm diameter, clean base
- Erythema: approx. 0.5–0.8 cm beyond ulcer edge
- Mild local warmth and tenderness
- No fluctuation, drainage, or necrosis
- DP/PT pulses palpable but slightly diminished compared to contralateral foot
- No systemic inflammatory response (afebrile, WBC 7.8 x10⁹/L)

### **Plain foot films:**

No soft tissue gas, cortical erosion, or periosteal reaction.

### **Question 1**

Given this presentation, how would you classify the severity of infection?

a) Mild — local signs of infection are present but not extensive, and no systemic signs
b) Mild — ulcer size <3 cm and no deep involvement
c) Moderate — based on ulcer size >2 cm and PAD suspicion
d) Cannot classify without vascular studies and MRI

✅ **Correct answer: a**
 📚 *IWGDF/IDSA 2023, Recommendation 1*

The resident asks how to document this using a formal system for severity assessment to guide treatment and monitor progress.

### **Question 2**

Which classification system is currently recommended in international guidelines for diabetic foot infection severity?

a) University of Texas staging system
b) IWGDF/IDSA infection classification
c) Wagner ulcer classification
d) PEDIS infection score

✅ **Correct answer: b**
📚 *IWGDF/IDSA 2023, Recommendation 1*

You plan to initiate empiric antibiotics and consider collecting cultures. There's no drainage from the wound.

### **Question 3**

What is the most appropriate method for microbiologic sampling in this context?

a) Superficial swab of the ulcer after cleaning
b) Deep tissue specimen via curettage or biopsy from ulcer base
c) Aspirate from peri-ulcer edema
d) Delay culture until purulence develops

✅ Correct answer: b

📚 IWGDF/IDSA 2023, Recommendation 5

A deep sample is obtained, and culture grows MSSA. The patient is started on oral dicloxacillin.

### **Question 4**

What is the recommended duration of antibiotic therapy for this patient?

a) 3 days, given the mild severity
b) 7–14 days, depending on clinical response
c) 4 weeks to ensure adequate treatment of deep infection
d) Until complete wound closure is achieved

✅ Correct answer: b

📚 IWGDF/IDSA 2023, Recommendation 12b

At 10-day reassessment, the ulcer shows granulation tissue and no further erythema. No signs of worsening. Glycemic management is ongoing.

### **Question 5**

What is the recommended approach to empiric antibiotic selection in mild DFI when culture data are not yet available?

a) Use the broadest-spectrum available agent to cover resistant organisms
b) Prioritize agents with superior tissue penetration even in early infection
c) Use likely pathogens and local antibiogram data to guide empiric choice
d) Initiate empiric therapy only if systemic signs develop

✅ Correct answer: c

📚 IWGDF/IDSA 2023, Recommendation 13

**CLINICAL CASE 1: Fracture related infection (FRI)**

**Guideline:**

1. ICM 2018 TRAUMA
2. Recommendations for Systemic Antimicrobial Therapy in Fracture-Related Infection: A Consensus From an International Expert Group (Consensus group 2020)

**Consensus Level:**

1. >90% agreement (Strongest Consensus or Unanimous) except for question number 3 where the consensus level is 85%.
2. Key Recommendations on Antimicrobial Therapy (Therapeutic Level V)

**Prompt:**

**You are acting as a board-certified infectious disease consultant.**
 Your role is to evaluate clinical scenarios presented to you by a practicing physician who is exploring how large language models like you can support decision-making in infectious disease care.

You will be given:

- A clinical case (including background, patient data, and key findings)
- A multiple-choice question with four options (a–d), only **one of which is correct**

Your task is to:

1. **Identify the single best answer** based on the clinical scenario.
2. **Justify your choice** using expert-level clinical reasoning, as if explaining your thought process to a fellow physician.
3. Keep your explanation under **500 words**, and avoid repeating the question or answer choices.
4. If evidence is unclear, acknowledge uncertainty and explain your reasoning as you would in a real consult.

Please format your response like this:

makefile

CopyEdit

Answer: [a / b / c / d]

Justification:
[Your clinical reasoning – max 500 words]

**Consult Request:**
 22-year-old male with open tibial fracture post high-impact trauma, now presenting with signs concerning for fracture-related infection. ID consult requested for diagnostic approach and antimicrobial management.

### **History of Present Illness:**

Mr. Steve Carter, a 22-year-old previously healthy male and active smoker (>20 pack-years), was brought in after a high-speed motorcycle collision. On arrival to the ED, he was alert (GCS 15), afebrile, hemodynamically stable, and saturating well on room air.

Initial trauma evaluation revealed an open fracture of the right lower leg with visible bone fragments. Radiographs confirmed a comminuted open tibia-fibula fracture. Orthopedic surgery classified it as a **Gustilo-Anderson Type III** injury and took the patient emergently to the OR for **irrigation, debridement, and internal fixation**.

### **Question 1**

Which perioperative prophylactic antibiotic regimen is most appropriate for this type of open fracture?

a) Cefazolin

b) Vancomycin

c) Vancomycin + Gentamicin

d) Ceftriaxone + Metronidazole

✅ **Correct answer:** d
📚 *ICM 2018 TRAUMA – Section 3.1, Question 1*

### **Postoperative Course:**

Prophylactic antibiotics were discontinued after intraoperative cultures returned negative. The patient remained clinically stable and was discharged with no signs of infection.

At his 4-week follow-up, he reports **new-onset leg pain, swelling, and localized erythema**. Exam shows warmth, tenderness over the surgical site, and fluctuance over the anterior tibia. No fever. Wound appears intact but mildly swollen.

### **Question 2**

How long should prophylactic antibiotics be administered following internal fixation of a Gustilo III open fracture?

a) 24 hours

b) 72 hours

c) 2 weeks

d) 4 weeks

✅ **Correct answer:** b
📚 *ICM 2018 TRAUMA – Section 3.1, Question 1*

The team is concerned about **possible fracture-related infection (FRI)**. They ask what should be done next.

### **Question 3**

Which of the following is **not** a recommended initial step in evaluating suspected FRI?

a) Order CRP, ESR, and WBC count

b) Obtain plain radiographs

c) Proceed directly to surgical exploration

d) Delay antibiotics and reassess in 1 week with labs

✅ **Correct answer:** c
📚 *ICM 2018 TRAUMA – Section 2, Question 2*

Based on clinical exam, inflammatory markers, and radiographic changes, the diagnosis of **fracture-related infection** is confirmed. The patient undergoes **hardware removal and debridement**. Cultures grow **methicillin-sensitive *Staphylococcus aureus* (MSSA)**. Cefazolin is initiated.

### **Question 4**

What is the appropriate systemic antibiotic duration for a confirmed FRI following surgical debridement?

a) 2 weeks

b) 6 weeks

c) 12 weeks

d) Begin indefinite suppressive therapy

✅ **Correct answer:** b
📚 *ICM 2018 TRAUMA – Section 3.1, Question 5*

The resident reviews literature on FRI management and asks which of the following statements is incorrect.

### **Question 5**

Which statement about fracture-related infection is **not** supported by current high-level evidence?

a) Up to 30% of FRI cases may be polymicrobial

b) Highly bioavailable agents are preferred when hardware cannot be removed

c) In Candida FRI, all foreign material should be removed

d) Multiple RCTs exist comparing surgical strategies and antibiotic durations in FRI

✅ **Correct answer:** d
📚 *ICM 2018 TRAUMA – Section 3.1, Question 1*

**CLINICAL CASE 2: Fracture related infection (FRI)**

**Guideline:**

1. ICM 2018 TRAUMA
2. Recommendations for Systemic Antimicrobial Therapy in Fracture-Related Infection: A Consensus From an International Expert Group (Consensus group 2020)

**Consensus Level:**

1. >90% agreement (Strongest Consensus or Unanimous)
2. Key Recommendations on Antimicrobial Therapy (Therapeutic Level V)

**Prompt:**

**You are acting as a board-certified infectious disease consultant.**
 Your role is to evaluate clinical scenarios presented to you by a practicing physician who is exploring how large language models like you can support decision-making in infectious disease care.

You will be given:

- A clinical case (including background, patient data, and key findings)
- A multiple-choice question with four options (a–d), only **one of which is correct**

Your task is to:

1. **Identify the single best answer** based on the clinical scenario.
2. **Justify your choice** using expert-level clinical reasoning, as if explaining your thought process to a fellow physician.
3. Keep your explanation under **500 words**, and avoid repeating the question or answer choices.
4. If evidence is unclear, acknowledge uncertainty and explain your reasoning as you would in a real consult.

Please format your response like this:

makefile

CopyEdit

Answer: [a / b / c / d]

Justification:
[Your clinical reasoning – max 500 words]

**Consult Request:**
 81-year-old woman with early signs of wound infection following hemiarthroplasty for femoral neck fracture. ID consult requested to guide surgical timing, empiric therapy, and decisions on oral step-down and suppressive treatment.

### **History of Present Illness:**

Mrs. Margaret O’Donnell, 81, was brought to the ED after a ground-level fall at home. Imaging showed a **displaced femoral neck fracture**, and she was taken to the OR for **urgent cemented hemiarthroplasty** within 12 hours of arrival.

Her medical history includes:

- **Type 2 diabetes** (HbA1c 7.6%)
- **Hypertension**
- **Chronic venous insufficiency**
- **Osteopenia**

Medications: metformin, amlodipine, aspirin 81 mg daily

Pre-op labs were unremarkable (WBC normal, CRP 12 mg/L, glucose 148 mg/dL).

### **Question 1**

How does the risk of surgical site infection (SSI) in this setting compare to **elective hip arthroplasty**?

a) Similar risk, as both receive prophylactic antibiotics and early surgery
 b) Lower risk, especially with urgent OR timing and short pre-op delay
 c) Significantly higher risk due to patient factors and trauma context
 d) No increased risk if appropriate antibiotic prophylaxis was administered

✅ **Correct answer:** c
📚 *ICM 2019 TRAUMA – Section 3.3, Question 2*

### **Postoperative Course:**

Discharged on post-op day 4. At 2-week follow-up, she reports **increased hip pain, swelling, and light drainage**. On exam, the wound appears erythematous, and labs show WBC 11.8 x10⁹/L and CRP 82 mg/L. She is afebrile.

Ortho plans to return to the OR and asks about surgical urgency.

### **Question 2**

When is **surgical debridement** indicated in this scenario?

a) Only after intraoperative cultures confirm deep infection
 b) Within 24 hours of when systemic signs develop

c) As soon as possible
d) Within 24 hours of hospital readmission if clinical status remains stable

✅ **Correct answer:** c
📚 *ICM 2019 TRAUMA – Section 3.4, Question 1*

### **Operative Management:**

She undergoes **surgical debridement with retention** of the prosthesis. Intraoperative samples are collected. The team asks for empiric antibiotic guidance pending final cultures.

### **Question 3**

What is the recommended empiric approach before cultures return?

a) Vancomycin + rifampin
b) Dalbavancin + gram-negative agent
c) Vancomycin + gram-negative agent
d) Daptomycin + gram-negative agent

✅ **Correct answer:** c
📚 *Consensus group 2020*



### **Microbiology:**

Cultures grow **ESBL-producing *Klebsiella pneumoniae***. Therapy is transitioned to **IV ertapenem**. After 5 days, the wound appears dry, and inflammatory markers are improving. The patient is afebrile and clinically stable.

You are asked whether she can be discharged on oral antibiotics.

### **Question 4**

When is it appropriate to **switch to oral therapy** in FRI?

a) After completing 14 days of IV therapy and symptom improvement
b) When the wound is dry and patient is afebrile
c) Once susceptibility results confirm a bioavailable oral agent
d) Only after surgical wound closure is complete

✅ **Correct answer:** c
📚 *Consensus group 2020*

### **Long-Term Planning:**

During rounds, a resident proposes lifelong suppressive therapy, citing the patient’s age and retained hardware.

### **Question 5**

How should **suppressive antibiotic therapy (SAT)** be approached in FRI?

a) Appropriate in elderly patients with hardware, regardless of infection control
 b) Indicated if more than one organism was isolated from deep tissue
 c) Considered individually based on surgical outcome, pathogen, and host status
 d) Contraindicated in the setting of prosthetic implants if surgery has been performed

✅ **Correct answer:** c
📚 *ICM 2019 TRAUMA – Section 3.1, Question 6*

**CLINICAL CASE 3: Fracture related infection (FRI)**

**Guideline:**

1. ICM 2018 TRAUMA
2. Recommendations for Systemic Antimicrobial Therapy in Fracture-Related Infection: A Consensus From an International Expert Group (Consensus group 2020)

**Consensus Level:**

1. >90% agreement (Strongest Consensus or Unanimous)
2. Key Recommendations on Antimicrobial Therapy (Therapeutic Level V)

**Prompt:**

**You are acting as a board-certified infectious disease consultant.**
 Your role is to evaluate clinical scenarios presented to you by a practicing physician who is exploring how large language models like you can support decision-making in infectious disease care.

You will be given:

- A clinical case (including background, patient data, and key findings)
- A multiple-choice question with four options (a–d), only **one of which is correct**

Your task is to:

1. **Identify the single best answer** based on the clinical scenario.
2. **Justify your choice** using expert-level clinical reasoning, as if explaining your thought process to a fellow physician.
3. Keep your explanation under **500 words**, and avoid repeating the question or answer choices.
4. If evidence is unclear, acknowledge uncertainty and explain your reasoning as you would in a real consult.

Please format your response like this:

makefile

CopyEdit

Answer: [a / b / c / d]

Justification:
[Your clinical reasoning – max 500 words]

**Consult Request:**
 A 38-year-old man with early signs of infection following open tibial fracture fixation. Orthopedic surgery requests infectious disease input regarding management strategy and long-term planning.

### **History of Present Illness:**

Mr. Jamal Robertson is a 38-year-old man who presented after a high-speed motorcycle accident. He sustained an **open midshaft tibial fracture**, which orthopedic surgery classified as **Gustilo IIIA**. There was no neurovascular compromise.

His past medical history includes:

- **Chronic smoking** (>30 pack-years)
- **Type 2 diabetes** (poorly controlled, last HbA1c unknown)
- **Hypertension**
- **Chronic kidney disease** (baseline Cr 1.6 mg/dL)

### **Initial Management:**

He underwent **urgent irrigation and debridement**, **external fixation**, and placement of **gentamicin-impregnated beads**. Empiric antibiotics with **cefazolin and metronidazole** were started in the ED.

### **Day 4 Post-op:**

Nursing reports **increased erythema and light seropurulent drainage** from the medial aspect of the wound. No foul odor, no necrosis. Patient is afebrile (T 36.8°C), HR 88 bpm, BP 124/78. The fixator is stable on inspection.

Laboratory results:

- WBC: 10.7 x10⁹/L
- CRP: 70 mg/L
- Creatinine: 1.8 mg/dL

The surgical resident asks if this could be an early FRI and whether antibiotics should be broadened or surgery planned.

### **Question 1**

Which of the following is a **recognized host-related risk factor** for developing fracture-related infection?

a) Diabetes and CKD
b) Hypertension and CKD
c) Smoking
d) Diabetes and hypertension

✅ **Correct answer:** c
📚 *ICM 2019 TRAUMA – Section 1.1, Question 1*

There is no significant pain. The patient is mobile on crutches and without systemic symptoms. Ortho feels the fixation is mechanically intact and well-positioned. No radiographic signs of loosening.

You are asked for your recommendation on whether to observe, escalate antimicrobials, or plan reoperation.

### **Question 2**

What is the most appropriate **next step** in management?

a) Await culture results before initiating surgical management

b) Perform debridement and retain external fixation if stable

c) Begin oral antibiotics and reassess in 48 hours

d) Continue IV cefazolin and monitor the drainage pattern

✅ **Correct answer:** b
📚 *ICM 2019 TRAUMA – Section 3.4, Question 4*

The patient undergoes irrigation and debridement, with hardware retained. Negative pressure wound therapy is initiated. Intraoperative cultures grow **MRSA** and **Enterobacter cloacae**. You start **vancomycin and cefepime**.

During rounds, a resident asks whether povidone-iodine irrigation could be added to improve wound sterilization.

### **Question 3**

What is the recommended irrigation approach during debridement of infected surgical wounds?

a) Povidone-iodine irrigation is preferred

b) Chlorhexidine irrigation is superior to saline

c) Normal saline irrigation; avoid cytotoxic agents

d) Saline is preferred, but cytotoxic agents may be added if resistant organisms are suspected

✅ **Correct answer:** c
📚 *ICM 2019 TRAUMA – Section 3.4, Question 3*

At the multidisciplinary meeting, ortho wants to classify this as “chronic osteomyelitis” based on the presence of a draining wound. Radiographs show no bone involvement. ID is asked to weigh in on classification and implications for treatment.

### **Question 4**

What is the best-supported interpretation of this case in terms of **acute vs chronic osteomyelitis**?

a) Chronic OM requires histologic confirmation of necrosis

b) Presence of sinus tract confirms chronic OM

c) Current literature lacks standardized definitions

d) Infection of <2 weeks is always acute OM

✅ **Correct answer:** c
📚 *ICM 2019 TRAUMA – Section 2.1, Question 4*

The patient improves clinically. You begin planning oral step-down therapy. The fellow suggests continuing cefepime “to be safe.” You opt to de-escalate.

### **Question 5**

What principle best reflects expert guidance on **antibiotic stewardship** in FRI management?

a) Broad-spectrum IV therapy should be continued through the full course

b) Narrow-spectrum agents are insufficient for mixed infections

c) Agent selection should be based solely on hospital antibiogram

d) Stewardship principles should guide antibiotic selection and duration

✅ **Correct answer:** d
📚 *Expert Consensus: Key recommendations on antimicrobial therapy*

**CLINICAL CASE 4: Fracture related infection (FRI)**

**Guideline:**

1. ICM 2018 TRAUMA
2. Recommendations for Systemic Antimicrobial Therapy in Fracture-Related Infection: A Consensus From an International Expert Group (Consensus group 2020)

**Consensus Level:**

1. >90% agreement (Strongest Consensus or Unanimous)
2. Key Recommendations on Antimicrobial Therapy (Therapeutic Level V)

**Prompt:**

**You are acting as a board-certified infectious disease consultant.**
 Your role is to evaluate clinical scenarios presented to you by a practicing physician who is exploring how large language models like you can support decision-making in infectious disease care.

You will be given:

- A clinical case (including background, patient data, and key findings)
- A multiple-choice question with four options (a–d), only **one of which is correct**

Your task is to:

1. **Identify the single best answer** based on the clinical scenario.
2. **Justify your choice** using expert-level clinical reasoning, as if explaining your thought process to a fellow physician.
3. Keep your explanation under **500 words**, and avoid repeating the question or answer choices.
4. If evidence is unclear, acknowledge uncertainty and explain your reasoning as you would in a real consult.

Please format your response like this:

makefile

CopyEdit

Answer: [a / b / c / d]

Justification:
[Your clinical reasoning – max 500 words]

**Consult Request:**
 46-year-old trauma patient with open abdominal injury and pelvic ring fracture. Infectious Diseases consult requested regarding fixation timing, MRSA decolonization, and antimicrobial strategy during staged orthopedic management.

### **History of Present Illness:**

Mr. Lukas Brandt, 46, was brought in by EMS after a **multi-vehicle highway collision**. He was hemodynamically unstable on arrival (SBP 80 mmHg, HR 118 bpm) with GCS 13. Primary survey revealed a **pelvic ring fracture** with associated **open abdominal trauma**. Exploratory **laparotomy was performed**, followed by **emergent external pelvic fixation** and **temporary open abdomen management using negative pressure dressings**.

### **Background:**

- Smoker
- Hypertension
- Known **MRSA carrier** (positive nares screening during prior admission)
- Medications: Lisinopril 10 mg daily, OTC NSAIDs

### **Clinical Discussion:**

Orthopedic surgery plans to proceed with **staged ORIF** for pelvic stabilization. They inquire whether the presence of a temporary open abdomen should delay internal fixation and what the infection risk implications are.

### **Question 1**

How should internal fixation be timed in the setting of a temporary open abdomen?

a) Delay due to bleeding risk
 b) Delay due to infection risk
 c) Internal fixation should not be delayed
 d) Cephalosporin should be initiated before fixation

✅ **Correct answer:** c
📚 *ICM 2019 TRAUMA – Section 1.2, Question 4*

### **Pre-op Planning:**

Given the patient’s **positive MRSA colonization history**, the surgical team raises concern about whether decolonization is necessary prior to definitive fixation.

### **Question 2**

What is the appropriate approach to **MRSA decolonization** in this setting?

a) Not recommended due to lack of benefit in trauma patients

b) Only required for elective procedures in low-risk hosts

c) May be considered, but should not delay surgery

d) Always required prior to orthopedic surgery involving hardware placement

✅ **Correct answer:** c
📚 *ICM 2019 TRAUMA – Section 1.2, Question 1*



**Post-op Day 3:**

Mr. Brandt undergoes **staged ORIF**. On day 3 post-op, he develops a **low-grade fever**, **CRP 122 mg/L**, and some **oozing at the incision site**. No hemodynamic instability. Wound is not purulent.

Ortho asks if empiric IV antibiotics should be started now or if further workup is needed first.

### **Question 3**

When should empiric **IV antibiotics** be initiated for suspected FRI?

a) After deep tissue samples are collected intraoperatively

b) After imaging confirms involvement of cortical bone

c) Based on CRP >100 mg/L and wound drainage

d) At first clinical suspicion, even if surgical sampling is not feasible

✅ **Correct answer:** a
📚 *Expert Consensus: Key recommendations on antimicrobial therapy*



**Cultures:**

Intraoperative samples grow **Enterococcus faecalis** and **Klebsiella aerogenes**. The patient had initially been started on **vancomycin + cefepime**, but susceptibilities show both organisms are sensitive to narrower options.

The ID fellow recommends stopping vancomycin. Surgery expresses concern about early de-escalation in a high-risk patient.

### **Question 4**

What is the correct principle guiding **antibiotic de-escalation** in FRI?

a) Continue empiric therapy for at least 14 days in high-risk patients

b) Switch to targeted therapy once susceptibility data are available

c) Maintain vancomycin due to Enterococcus and Gram-negative co-infection

d) Discontinue all IV therapy if patient is afebrile and cultures are pending

✅ **Correct answer:** b
📚 *Expert Consensus: Key recommendations on antimicrobial therapy*



**Long-term Plan:**

The team begins planning for **oral step-down** and wants guidance on how to tailor the antimicrobial regimen. They ask whether surgical details or pathogen features should carry more weight.

### **Question 5**

Which factors should guide **targeted antimicrobial therapy** in FRI?

a) Institutional antibiogram and IV-to-oral bioavailability

b) Pathogen and surgical approach (e.g., retention vs. removal)

c) Use the broadest available oral agents to reduce relapse

d) Antifungal prophylaxis is recommended in open pelvic fractures

✅ **Correct answer:** b
📚 *Expert Consensus: Key recommendations on antimicrobial therapy*

**CLINICAL CASE 5: Fracture related infection (FRI)**

**Guideline:**

1. ICM 2018 TRAUMA
2. Recommendations for Systemic Antimicrobial Therapy in Fracture-Related Infection: A Consensus From an International Expert Group (Consensus group 2020)

**Consensus Level:**

1. >90% agreement (Strongest Consensus or Unanimous)
2. Key Recommendations on Antimicrobial Therapy (Therapeutic Level V)

**Patient Information**

- **Name:** Ms. Rachel Kim
- **Age:** 84 years
- **Sex:** Female
- **Medical History:** Anemia of chronic disease, Mild Cognitive Impairment (MCI), COPD, prior CABG, on aspirin
- **Medications:** Aspirin 100 mg, inhaled corticosteroids

**Prompt:**

**You are acting as a board-certified infectious disease consultant.**
 Your role is to evaluate clinical scenarios presented to you by a practicing physician who is exploring how large language models like you can support decision-making in infectious disease care.

You will be given:

- A clinical case (including background, patient data, and key findings)
- A multiple-choice question with four options (a–d), only **one of which is correct**

Your task is to:

1. **Identify the single best answer** based on the clinical scenario.
2. **Justify your choice** using expert-level clinical reasoning, as if explaining your thought process to a fellow physician.
3. Keep your explanation under **500 words**, and avoid repeating the question or answer choices.
4. If evidence is unclear, acknowledge uncertainty and explain your reasoning as you would in a real consult.

Please format your response like this:

makefile

CopyEdit

Answer: [a / b / c / d]

Justification:
[Your clinical reasoning – max 500 words]



**Consult Request:**
 84-year-old woman with open bimalleolar ankle fracture, recent ORIF, and delayed wound healing. ID consult requested to support evaluation of possible early FRI, wound management strategy, and transfusion-related risks.

### **History of Present Illness:**

Ms. Rachel Kim, an 84-year-old woman with **moderate dementia**, **COPD**, **anemia of chronic disease**, and a history of **CABG**, was admitted after falling off a step-ladder while attempting to close a kitchen cabinet. On arrival, she was found to have a **Gustilo II open bimalleolar fracture** of the right ankle.

### **Initial Evaluation:**

- BP: 105/60 mmHg
- HR: 92 bpm
- Hb: 8.1 g/dL

She was taken to the OR for **urgent irrigation, debridement, and ORIF**. She received **2 units of PRBCs** intraoperatively. Medications included **aspirin 100 mg daily** and inhaled corticosteroids.

### **Question 1**

What are the key predictors for requiring **allogeneic blood transfusion (ABT)** in this patient?

a) Gustilo II open fracture and anemia

b) Antiaggregant use and anemia

c) Anemia, Gustilo II open fracture, and antiaggregant use

d) MCI, antiaggregant use, and anemia

✅ **Correct answer:** b
📚 *ICM 2019 TRAUMA – Section 1.1, Question 6*

### **Post-Op Day 8:**

The patient remains afebrile but reports **increased ankle pain** and **mild drainage** from the wound site. WBC: 9.3 x10⁹/L, CRP: 56 mg/L.

The surgical team aspirates the joint space. The fluid is **bloody but not overtly purulent**. The junior resident asks if hematoma fluid has diagnostic value.

### **Question 2**

How should the aspirated hematoma be interpreted?

a) It is sterile unless grossly purulent

b) It should be processed as potentially infectious material

c) It has no diagnostic utility

d) It should be discarded prior to wound exploration

✅ **Correct answer:** b
📚 *ICM 2019 TRAUMA – Section 2, Question 5*

### **Wound Management:**

Cultures return **Staphylococcus epidermidis** and **Acinetobacter baumannii**. The wound is managed temporarily with **VAC therapy**. The fellow asks whether VAC increases the risk of **hardware colonization**.

### **Question 3**

What does current evidence say about VAC and **metal colonization**?

a) VAC systems increase colonization risk

b) There is no conclusive evidence VAC increases colonization

c) VAC is contraindicated in any implant-related wound

d) VAC reduces the need for systemic antibiotics

✅ **Correct answer:** b
📚 *ICM 2019 TRAUMA – Section 2, Question 6*

### **Classification and Planning:**

With infection signs stabilizing and oral antibiotics planned, the team begins **discussing definitive fixation**. They ask whether reclassification is needed before the next surgical step.

### **Question 4**

What is the benefit of using the **OTA-OFC** classification compared to **Gustilo** in this case?

a) Gustilo is superior for infection risk prediction

b) OTA-OFC provides broader injury characterization

c) They are equivalent; reclassification is unnecessary

d) OTA-OFC is outdated for open fractures

✅ **Correct answer:** b
📚 *ICM 2019 TRAUMA – Section 2, Question 1*

### **Risk Assessment:**

Before reoperation, the team reviews Ms. Kim’s **initial transfusion** and asks whether she’s at **increased risk for needing ABT again**.

### **Question 5**

Which of the following is **not** a recognized predictor for ABT in patients undergoing periprosthetic fracture surgery?

a) Female sex and low BMI

b) Hip arthroplasty with prolonged surgical time

c) Anemia and high comorbidity index

d) Use of tranexamic acid and hypotensive anesthesia

✅ **Correct answer:** d

📚 ICM 2019 TRAUMA – Section 3.3, Question 1

**CLINICAL CASE 6: Fracture related infection (FRI)**

**Guideline:**

1. ICM 2018 TRAUMA
2. Recommendations for Systemic Antimicrobial Therapy in Fracture-Related Infection: A Consensus From an International Expert Group (Consensus group 2020)

**Consensus Level:**

1. >90% agreement (Strongest Consensus or Unanimous)
2. Key Recommendations on Antimicrobial Therapy (Therapeutic Level V)

**Prompt:**

**You are acting as a board-certified infectious disease consultant.**
 Your role is to evaluate clinical scenarios presented to you by a practicing physician who is exploring how large language models like you can support decision-making in infectious disease care.

You will be given:

- A clinical case (including background, patient data, and key findings)
- A multiple-choice question with four options (a–d), only **one of which is correct**

Your task is to:

1. **Identify the single best answer** based on the clinical scenario.
2. **Justify your choice** using expert-level clinical reasoning, as if explaining your thought process to a fellow physician.
3. Keep your explanation under **500 words**, and avoid repeating the question or answer choices.
4. If evidence is unclear, acknowledge uncertainty and explain your reasoning as you would in a real consult.

Please format your response like this:

makefile

CopyEdit

Answer: [a / b / c / d]

Justification:
[Your clinical reasoning – max 500 words]

**Consult Request:**
 73-year-old man with poor baseline function and multiple comorbidities is admitted for traumatic femoral shaft fracture. ID consult requested to assist with surgical risk assessment, infection prevention, and postoperative management after signs of infection appear.

### **Background:**

Mr. Alfonso Graziano, 73, was admitted following a **low-energy fall** during a bed transfer in his skilled nursing facility. He has a history of:

- **Stroke** with residual left hemiparesis
- **Advanced dementia**
- **Type 2 diabetes mellitus**

He is **bedbound**, has a **BMI of 18.2**, and on admission had **multiple stage 2 pressure ulcers** (sacral and bilateral heels). His **serum albumin is 2.4 g/dL**.

Medications: metformin, atorvastatin, donepezil

Labs:

- WBC: 8.9 x10⁹/L
- CRP: 32 mg/L
- Hb: 10.4 g/dL

### **Course:**

Orthopedic surgery proceeds with **intramedullary nailing**. On **post-op day 3**, the patient develops **purulent drainage** from the surgical site.

Pre-operative notes reveal a **diagnosed UTI** at admission, but **no targeted therapy** was administered.

### **Question 1**

How does **nutritional status** impact the risk and outcome of fracture-related infection (FRI)?

a) Nutritional support helps prevent infection even in well-nourished individuals
b) Nutritional supplementation should be started if deficiencies are identified
c) Nutritional screening is optional in trauma patients
d) Caloric intake is more critical than protein levels



✅ **Correct answer:** b
📚 *ICM 2019 TRAUMA – Section 1.1, Question 2*



The fellow notes the **untreated UTI** and asks whether this could have contributed to the surgical site infection.

### **Question 2**

What does current evidence suggest about **untreated infections** (e.g., UTI, pneumonia) prior to orthopedic surgery?

a) No impact if the wound is clean
 b) Risk is low unless bacteremia is present
 c) Data are limited, but an association with SSI/PJI is possible
 d) There is no supporting evidence of any risk

✅ **Correct answer:** c
📚 *ICM 2019 TRAUMA – Section 1.1, Question 3*



Intraoperative cultures return **MRSA**, and vancomycin is started. The team wonders whether the patient's profile explains his pathogen risk.

### **Question 3**

What best summarizes **organism-specific risk factors** for infection in trauma surgery?

a) MRSA colonization does not affect risk or prophylaxis
 b) Surgical site location may influence infection risk and should guide coverage
 c) External fixators don’t change infection risk or antibiotic selection
 d) Open fracture severity is the only variable affecting prophylaxis choice

✅ **Correct answer: b**
 📚 *ICM 2019 TRAUMA – Section 1.1, Question 4*

After debridement and lavage, the orthopedic team plans for future hardware revision and asks about fixation method selection to minimize reinfection risk.

### **Question 4**

Which of the following best reflects current guidance on **internal vs. external fixation** in infection-prone trauma patients?

a) Internal fixation is always safer
 b) Internal fixation is generally preferred
 c) Infection risk is equivalent between the two
 d) External fixation is contraindicated in open fractures

✅ **Correct answer:** b
📚 *ICM 2019 TRAUMA – Section 1.2, Question 3*



**CLINICAL CASE 1: Prosthetic joint infection (PJI)**

**Guideline:**

1. ICM 2018 PJI
2. A Guide to Utilization of the Microbiology Laboratory for Diagnosis of Infectious Diseases: 2018 Update by the Infectious Diseases Society of America and the American Society for Microbiology (2018 IDSA micro)

**Consensus Level:**

1. >90% agreement (Strongest Consensus or Unanimous)
2. The guideline does not provide level of evidence

**Prompt:**

**You are acting as a board-certified infectious disease consultant.**
 Your role is to evaluate clinical scenarios presented to you by a practicing physician who is exploring how large language models like you can support decision-making in infectious disease care.

You will be given:

- A clinical case (including background, patient data, and key findings)
- A multiple-choice question with four options (a–d), only **one of which is correct**

Your task is to:

1. **Identify the single best answer** based on the clinical scenario.
2. **Justify your choice** using expert-level clinical reasoning, as if explaining your thought process to a fellow physician.
3. Keep your explanation under **500 words**, and avoid repeating the question or answer choices.
4. If evidence is unclear, acknowledge uncertainty and explain your reasoning as you would in a real consult.

Please format your response like this:

makefile

CopyEdit

Answer: [a / b / c / d]

Justification:
[Your clinical reasoning – max 500 words]

**Patient Information**

Mr. John Doe, a 67-year-old man, comes to your clinic with progressive left hip pain. He’s been struggling for months, with increasing stiffness, difficulty walking, and discomfort that now disturbs his sleep. Conservative management, including NSAIDs and physiotherapy, has provided no relief.

He has a history of **type 2 diabetes mellitus** (HbA1c 9.0), **hypertension**, **aortic valve replacement** (5 years ago), and a **previous episode of MSSA cellulitis** (2 years ago). Current medications include **metformin 500 mg BID** and **amlodipine 5 mg BID**.

After evaluating the limited response to nonoperative care and the significant functional decline, orthopedic surgery schedules him for **elective total hip arthroplasty (THA)**.

Prior to surgery, the team reaches out for ID input regarding perioperative antibiotic prophylaxis. You recommend a standard **single 2g IV dose of cefazolin** preoperatively. The surgeon agrees but wonders if the patient’s diabetes and cardiac history might justify **a 5-day course of oral antibiotics postoperatively** to reduce infection risk.

**Question 1:**

Is there any justification for the extended use of oral antibiotics after primary total joint arthroplasty (TJA)?

a) No, there is no indication especially for routine surgery
 b) Yes, because this patient had a history of MSSA infection
 c) Yes, because current diabetes is not under good control (HbA1c 9.0)
 d) Yes, because this patient had a history of cardiac valve replacement

✅ **Correct Answer:** a**.**
📖 *ICM 2018 PJI - Section 1.3, question 8*

The surgery proceeds uneventfully, and Mr. Doe is discharged on postoperative day three with standard instructions and outpatient physical therapy. At his **2-month follow-up**, however, he reports **new pain localized to the proximal thigh** and increasing difficulty walking over the past 5 days.

He is afebrile (36.0°C), normotensive, and his physical exam reveals **localized warmth, swelling, and erythema**, with **purulent drainage at the surgical site**.

**Question 2:**

In the suspicion of a periprosthetic joint infection (PJI), which clinical finding is the most sensitive for diagnosis?

a) Pain
 b) Reduced range of motion
 c) Erythema
 d) Purulence

✅ **Correct Answer:** a**.**

📖 *ICM 2018 PJI - section 2.1, question 5*

**Question 3:**

In the suspicion of a periprosthetic joint infection (PJI), which clinical finding is the most specific for diagnosis?

a) Pain
 b) Reduced range of motion
 c) Erythema
 d) Purulence

✅ **Correct Answer:** d**.**

📖 *ICM 2018 PJI - section 2.1, question 5*

To distinguish between superficial and deep surgical site infection, the orthopedic team performs a clinical evaluation and consults you about the most appropriate diagnostic step.

**Question 4:**

What test is the recommended approach to differentiate, in this case, superficial from deep site infection?

a) Clinical evaluation and joint aspiration

b) Clinical evaluation and ultrasound – may detect fluid but lacks specificity

c) Clinical evaluation and CT scan – less useful for soft tissue/joint distinction

d) Clinical evaluation and Surgical Wound Aspect Score – not validated for PJI

✅ **Correct Answer:** a**.**

📖 *ICM 2018 PJI - section 2.1, question 3*

A **joint aspiration** confirms:

- Synovial WBC > 30,000 cells/μL
- PMN count > 90%
- Culture: *Methicillin-sensitive Staphylococcus aureus* (MSSA)

A diagnosis of **periprosthetic joint infection** is made. Surgical evaluation leads to a decision for a **two-stage exchange** with placement of a cement spacer.

You initiate **IV cefazolin 2g q8h**. The surgeon suggests adding **rifampin** to improve biofilm coverage.

**Question 5:**

What is the appropriate recommendation for this surgeon?

a) Yes, because this patient has MSSA PJI

b) Yes, because patient has history of MSSA infection with history of cardiac valve replacement

c) No, because it was placed a cement spacer

d) No, because rifampin is indicated only for DAIR and one-stage exchange

✅ **Correct Answer:** d**.**
📖 *ICM 2018 PJI - section 5.5, question 6*

After 6 weeks of antibiotics, the patient shows gradual improvement. At the 2-week follow-up, there’s some **residual erythema** and **mild tenderness**, with **CRP 80 mg/L**, **WBC 9.5 x10⁹/L**, and **ESR 45 mm/hr**.

At week 4, **CRP is 40 mg/L**, and **pain has improved**, though **range of motion remains limited**. By the 6-week mark, the patient completes antibiotics.

Two weeks later, just prior to planned reimplantation, labs show: **CRP 25 mg/L**, **ESR 30 mm/hr**, and **WBC 8.0 x10⁹/L**. The surgeon expresses concern about residual inflammation and seeks your input.

**Question 6:**

Which of the following recommendations is most appropriate?

a) Proceed with the reimplantation
 b) Proceed with reimplantation after 2–3 days of antibiotic therapy
 c) Recommend delaying surgery until inflammatory markers normalize
 d) Recommend restarting antibiotic therapy and reassessing after two weeks

✅ **Correct Answer:** a**.**

📖 *ICM 2018 PJI - section 2.5, question 3*

**CLINICAL CASE 2: Prosthetic joint infection (PJI)**

**Guideline:**

1. ICM 2018 PJI
2. A Guide to Utilization of the Microbiology Laboratory for Diagnosis of Infectious Diseases: 2018 Update by the Infectious Diseases Society of America and the American Society for Microbiology (2018 IDSA micro)

**Consensus Level:**

1. >90% agreement (Strongest Consensus or Unanimous) except for question number 3 where the consensus level is 85%.
2. The guideline does not provide level of evidence

**Prompt:**

**You are acting as a board-certified infectious disease consultant.**
 Your role is to evaluate clinical scenarios presented to you by a practicing physician who is exploring how large language models like you can support decision-making in infectious disease care.

You will be given:

- A clinical case (including background, patient data, and key findings)
- A multiple-choice question with four options (a–d), only **one of which is correct**

Your task is to:

1. **Identify the single best answer** based on the clinical scenario.
2. **Justify your choice** using expert-level clinical reasoning, as if explaining your thought process to a fellow physician.
3. Keep your explanation under **500 words**, and avoid repeating the question or answer choices.
4. If evidence is unclear, acknowledge uncertainty and explain your reasoning as you would in a real consult.

Please format your response like this:

makefile

CopyEdit

Answer: [a / b / c / d]

Justification:
[Your clinical reasoning – max 500 words]

**Patient Information**
Ms. Carter, a 72-year-old woman, is referred to orthopedic surgery due to worsening **right knee pain**. She reports progressive functional decline, particularly with walking and stair use, and poor sleep due to nocturnal discomfort. Conservative management, including physical therapy, weight loss counseling, and corticosteroid injections (the last one over six months ago), has failed to improve her symptoms. Radiographs confirm **advanced tricompartmental osteoarthritis**.

Her medical history includes **morbid obesity (BMI 42.4)**, **hypertension**, **recurrent urinary tract infections**, and **GERD**. She underwent **remote cholecystectomy** and denies drug allergies. Medications include **lisinopril**, **omeprazole**, **vitamin D**, and **loratadine as needed**.

Routine preoperative labs return within normal range, with **albumin 4.1 g/dL** and **HbA1c 6.7%**. Her urinalysis is unremarkable. Given her overall good control of comorbidities, the surgical team is optimistic about proceeding with total knee arthroplasty and asks for your input from an infectious diseases perspective.



**Question 1:**

What is the most appropriate recommendation regarding surgery?

a) Proceed with surgery after administering preoperative intravenous cefazolin
 b) Proceed with surgery but add vancomycin due to elevated BMI
 c) Delay surgery because morbid obesity is an absolute contraindication to TJA
 d) Proceed with surgery because other comorbidities are controlled

✅ **Correct Answer:** c**.**
📖 *ICM 2018 PJI— Section 1.1, Question 1*



Following a **five-month prehabilitation program**, Ms. Carter reduces her BMI to **38.5**, and the surgical team proceeds with **elective right total knee arthroplasty (TKA)** under spinal anesthesia. She receives **2g of IV cefazolin 45 minutes prior to incision**. The operation is uneventful.

Postoperative labs are within expected limits: **WBC 8.4 ×10⁹/L**, **creatinine 0.9 mg/dL**, and **hemoglobin 11.2 g/dL**. She is discharged on day 2 with rivaroxaban for DVT prophylaxis and instructions for home-based physical therapy.

At her **6-week follow-up**, she reports mild stiffness and difficulty completing PT. More concerningly, she mentions **two days of clear fluid draining** from the inferior edge of the surgical scar. She denies fever, chills, or malaise.

Vitals are stable (T 36.8°C, HR 78 bpm, BP 120/70). On exam, there’s **mild erythema** and **a small area of serous drainage** along the incision line. Labs show **CRP 36 mg/L**, **ESR 52 mm/hr**, and **WBC 9.2 ×10⁹/L**.

**Question 2:**

What is the most appropriate next step?

a) Begin empiric oral antibiotics for presumed superficial cellulitis
 b) Perform joint aspiration with fluid sent for cell count and culture
 c) Order ultrasound to assess depth of involvement
 d) Reassure and recheck inflammatory markers in one week

✅ **Correct Answer:** b**.**
📖 *ICM 2018 PJI* — Section 2.1, Question 3



Joint aspiration yields **turbid synovial fluid** with a **WBC count of 35,000 cells/μL** and **PMN 92%**. Gram stain is pending. The orthopedic team schedules her for **surgical irrigation and debridement** the next morning. During pre-op planning, a resident asks you about best practices for microbiological sampling in this setting.



**Question 3:**

What is the recommended intraoperative microbiological sampling method?

a) One deep swab and synovial aspirate
 b) Two tissue samples plus synovial fluid culture
 c) At least three to four separate tissue samples for culture
 d) Synovial fluid into two anaerobic bottles

✅ **Correct Answer:** c**.**
📖 2018 IDSA micro



During the procedure, **all modular components are exchanged**, and the joint is irrigated with **3 liters of saline**. The attending surgeon pauses and asks whether that volume is adequate or if further irrigation is recommended.



**Question 4:**

What is the minimum recommended volume of irrigation for this procedure?

a) 1–2 liters
 b) 3–4 liters
 c) 6–9 liters
 d) >10 liters only if purulence is visible

✅ **Correct Answer:** c**.**
📖 *ICM 2018 PJI* — Section 5.2, Question 5

Three days post-op, Ms. Carter is clinically stable, afebrile, ambulating with assistance, and tolerating oral intake. Of the **four intraoperative tissue cultures collected**, **only one** grows *Cutibacterium acnes*. Synovial cultures remain negative, and Gram stain is unremarkable. The orthopedic attending asks for guidance on how to interpret this isolated culture.



**Question 5:**

What is the most appropriate interpretation of this single positive culture?

a) Evidence of persistent infection requiring prolonged IV antibiotics
 b) Confirmatory of PJI and warrants antifungal therapy
 c) Suggestive of contamination unless supported by additional findings
 d) Diagnostic for deep fungal PJI

✅ **Correct Answer:** c**.**

📖 2018 IDSA micro

**CLINICAL CASE 3: Prosthetic joint infection (PJI)**

**Guideline:**

1. ICM 2018 PJI
2. A Guide to Utilization of the Microbiology Laboratory for Diagnosis of Infectious Diseases: 2018 Update by the Infectious Diseases Society of America and the American Society for Microbiology (2018 IDSA micro)

**Consensus Level:**

1. >90% agreement (Strongest Consensus or Unanimous) except for question number 3 where the consensus level is 85%.
2. The guideline does not provide level of evidence

**Prompt:**

**You are acting as a board-certified infectious disease consultant.**
 Your role is to evaluate clinical scenarios presented to you by a practicing physician who is exploring how large language models like you can support decision-making in infectious disease care.

You will be given:

- A clinical case (including background, patient data, and key findings)
- A multiple-choice question with four options (a–d), only **one of which is correct**

Your task is to:

1. **Identify the single best answer** based on the clinical scenario.
2. **Justify your choice** using expert-level clinical reasoning, as if explaining your thought process to a fellow physician.
3. Keep your explanation under **500 words**, and avoid repeating the question or answer choices.
4. If evidence is unclear, acknowledge uncertainty and explain your reasoning as you would in a real consult.

Please format your response like this:

makefile

CopyEdit

Answer: [a / b / c / d]

Justification:
[Your clinical reasoning – max 500 words]

**Patient Information**
Mr. Thompson, a 75-year-old man, presents to the emergency department complaining of **acute right hip pain** for the past three days. He notes progressive groin stiffness and difficulty walking. He denies trauma, recent fever, or systemic symptoms, but mentions undergoing a **dental extraction 10 days ago** for an infected molar — without antibiotic prophylaxis.

His past medical history includes a **right total hip arthroplasty (14 months ago)**, **left total knee arthroplasty (3 years ago)**, **type 2 diabetes (HbA1c 7.4%)**, **hypertension**, **atrial fibrillation** (on **apixaban**), and **chronic prostatitis**. He has no known drug allergies.

Vitals are unremarkable: afebrile at 36.9°C, HR 84, BP 130/78. The right hip is tender on passive range of motion, with limited mobility. No overlying erythema or drainage is observed. The left knee is non-tender with full motion.

Initial labs:

- **WBC**: 9.8 ×10⁹/L
- **CRP**: 22 mg/L
- **ESR**: 36 mm/hr
- **Creatinine**: 0.9 mg/dL
- **LFTs and urinalysis**: unremarkable
- **Blood cultures**: pending



**Question 1:**

Given the current presentation, which of the following is the most appropriate next step?

a) Defer hip aspiration until inflammatory markers exceed diagnostic thresholds to avoid procedure-related infection
 b) Order CT of the hip and repeat blood tests in 48 hours
 c) Perform hip aspiration as part of the initial workup for suspected PJI
 d) Start empirically Vancomycin + Ceftriaxone due to dental extraction history

✅ **Correct Answer:** c**.**
📖 *ICM 2018 PJI*— Section 2.2, Question 2



A diagnostic **hip aspiration** is performed, yielding **cloudy synovial fluid**.

- **Synovial WBC**: 18,000 cells/μL
- **PMN**: 89%
- **Gram stain**: no organisms seen
- **Cultures**: pending

The orthopedic team calls for ID input, wondering whether this is **aseptic failure** or **possible late hematogenous infection**.



**Question 2:**

The orthopedic surgeon asks whether low inflammatory markers exclude PJI in this case. What do you reply?

a) Yes, CRP and ESR are below thresholds and rule out PJI
 b) No, inflammatory markers may be falsely low in late infections
 c) Yes, because synovial WBC count is also not definitive
 d) No, unless WBC is elevated, PJI cannot be ruled out

✅ **Correct Answer:** b**.**
📖 *ICM 2018 PJI*— Section 2.3, Question 2

You explain that **low-grade or hematogenous infections**, particularly those caused by indolent organisms, may not result in a robust inflammatory response. Given the dental work and new joint symptoms, clinical suspicion remains high.

After 72 hours, **blood cultures return negative**, and **synovial fluid cultures** are still pending. The team asks how to proceed in the event of **culture-negative PJI**.



**Question 3:**

What is the most appropriate diagnostic strategy for possible culture-negative PJI?

a) Repeat aspiration and begin antifungal therapy
b) Assume aseptic failure if cultures remain negative after 5 days
c) Extend culture incubation and consider molecular diagnostics
d) Repeat blood cultures and treat based on inflammatory markers

✅ **Correct Answer:** c**.**
📖 *ICM 2018 PJI*— Section 2.2, Question 6



During reassessment, you note the patient also has a **left TKA** placed three years ago. He denies any symptoms in that joint. A junior resident wonders if workup of the contralateral prosthesis is indicated due to the concern for hematogenous spread.



**Question 4:**

What is the appropriate recommendation regarding the asymptomatic prosthetic knee?

a) Perform aspiration regardless of symptoms due to recent bacteremia
 b) No further testing unless new symptoms emerge
 c) Examine clinically and consider aspiration only if suspicion remains
 d) Start empirical antibiotics to prevent bilateral seeding

✅ **Correct Answer:** c**.**

📖 *ICM 2018 PJI*— Section 2.2, Question 4



Despite negative cultures, the clinical team decides to proceed with **resection arthroplasty** and placement of a **spacer**, based on ongoing concern for infection. In anticipation of **second-stage reimplantation**, the surgeon inquires about **advanced diagnostic tools** to improve microbiological yield, particularly if standard cultures remain negative.



**Question 5:**

Which of the following best describes the role of sonication in this setting?

a) Sonication can be used only if 3–4 separate tissue samples shouldn’t be taken
b) Sonication is discouraged due to its high contamination risk and limited diagnostic value
c) Sonication of explanted prostheses can enhance detection of biofilm-associated pathogens and should be considered alongside standard tissue cultures
d) Sonication is only recommended if preoperative synovial fluid cultures are positive

✅ **Correct Answer:** c**.**
📖 *2018 IDSA micro*



**CLINICAL CASE 4: Prosthetic joint infection (PJI)**

**Guideline:**

1. ICM 2018 PJI
2. A Guide to Utilization of the Microbiology Laboratory for Diagnosis of Infectious Diseases: 2018 Update by the Infectious Diseases Society of America and the American Society for Microbiology (2018 IDSA micro)

**Consensus Level:**

1. >90% agreement (Strongest Consensus or Unanimous) except for question number 3 where the consensus level is 85%.
2. The guideline does not provide level of evidence

**Prompt:**

**You are acting as a board-certified infectious disease consultant.**
 Your role is to evaluate clinical scenarios presented to you by a practicing physician who is exploring how large language models like you can support decision-making in infectious disease care.

You will be given:

- A clinical case (including background, patient data, and key findings)
- A multiple-choice question with four options (a–d), only **one of which is correct**

Your task is to:

1. **Identify the single best answer** based on the clinical scenario.
2. **Justify your choice** using expert-level clinical reasoning, as if explaining your thought process to a fellow physician.
3. Keep your explanation under **500 words**, and avoid repeating the question or answer choices.
4. If evidence is unclear, acknowledge uncertainty and explain your reasoning as you would in a real consult.

Please format your response like this:

makefile

CopyEdit

Answer: [a / b / c / d]

Justification:
[Your clinical reasoning – max 500 words]

**Patient Information**
Ms. Hanley, a 79-year-old woman, is admitted with **progressive pain, swelling, and stiffness in her left knee**, which began roughly two weeks ago. She also reports **night sweats** and **fatigue**, though she hasn’t had fever or recent trauma. Her past medical history includes **left total knee arthroplasty (9 months prior)**, **coronary artery disease** (PCI 5 years ago), **CKD stage 3 (baseline Cr 1.4 mg/dL)**, **osteoporosis**, and **depression**.

On examination, she is afebrile but has a mild tachycardia. The **left knee is warm, tender, and demonstrates limited range of motion**, though there is **no sinus tract or drainage**.

Initial labs:

- **WBC**: 10.4 ×10⁹/L
- **CRP**: 65 mg/L
- **ESR**: 84 mm/hr
- **Creatinine**: 1.5 mg/dL
- **Hemoglobin**: 10.8 g/dL

Arthrocentesis yields frankly purulent synovial fluid, and Gram stain shows both gram-positive cocci in clusters and gram-negative rods. The orthopedic team proceeds with an urgent DAIR (debridement, antibiotics, and implant retention) procedure, including modular component exchange.

Intraoperative cultures grow methicillin-resistant Staphylococcus aureus (MRSA) and Escherichia coli. Blood cultures are negative.

**Question 1:**

What is the most appropriate antimicrobial plan regarding duration of therapy after this DAIR?

a) 14 days of IV therapy followed by oral suppressive antibiotics
b) At least 6 weeks of antimicrobial therapy targeting both organisms
c) 3 months of IV therapy due to polymicrobial infection
d) At least 6 weeks of IV therapy followed by 2 weeks of oral antibiotics

✅ **Correct Answer:** b**.**
📖 ICM 2018 PJI— Section 5.2, Question 12

You start the patient on **IV vancomycin and cefepime**, pending susceptibility testing. Over the next few days, she remains clinically stable, afebrile, with decreasing CRP and WBC levels. Cultures confirm **MRSA** and **fluoroquinolone-susceptible E. coli**. The team wants to understand the clinical relevance of **polymicrobial infection** in this setting.

**Question 2:**

What is the implication of this polymicrobial infection on treatment outcome?

a) No impact on outcome compared to monomicrobial infection
b) Increased risk of prosthesis loosening only
c) Improved outcome due to broader empiric coverage
d) Inferior outcome compared to monomicrobial PJI

✅ **Correct Answer:** d**. Inferior outcome compared to monomicrobial PJI**
📖 ICM 2018 PJI —Section 3, Question 2

On hospital day 5, the orthopedic attending raises the possibility of **adding rifampin** to enhance **biofilm penetration**, particularly for MRSA. The patient is tolerating the current regimen (vancomycin and cefepime) without issue.

**Question 3:**

What is the most appropriate approach to rifampin use in this context?

a) Add rifampin as monotherapy to reduce resistance risk
b) Add rifampin immediately after culture positivity regardless of other agents
c) Add rifampin only in combination and if fluoroquinolone is part of regimen
d) Avoid rifampin due to E. coli co-infection

✅ **Correct Answer:** c**.**
📖 ICM 2018 PJI — Section 5.9, Question 6

After two weeks of therapy, her renal function remains stable (**Cr 1.4 mg/dL**), and she continues to improve functionally. Given her age, comorbidities, and high surgical risk, the orthopedic team wonders whether **chronic suppressive therapy** could be considered should the infection recur or not fully resolve.

**Question 4:**

In which situation would long-term suppressive antimicrobial therapy be appropriate?

a) After 2 weeks of IV therapy as a substitute for full treatment in fungal infection
b) When the patient is clinically well and cultures are negative
c) In patients not eligible for surgery or who decline further intervention
d) In cases of culture-negative infections

✅ **Correct Answer:** c**.**
📖 ICM 2018 PJI — Section 5.11, Question 3

During discharge planning, a fellow asks whether this MRSA infection might have been anticipated based on **regional resistance patterns**. You recall recent epidemiologic data comparing global variation in **MRSA prevalence** in PJIs.

**Question 5:**

Which of the following statements about MRSA epidemiology is most accurate?

a) MRSA is rare in PJIs worldwide and associated with hospital outbreaks only
b) Europe has a higher incidence of MRSA PJI than the US or Australia
c) MRSA is more frequently seen in PJIs in the US and Australia than Europe
d) MRSA is common in PJIs only among immunocompromised patients

✅ **Correct Answer:** c**.**
📖 *ICM 2018 PJI*— Section 3, Question 4

**CLINICAL CASE 5: Prosthetic joint infection (PJI)**

**Guideline:**

1. ICM 2018 PJI
2. A Guide to Utilization of the Microbiology Laboratory for Diagnosis of Infectious Diseases: 2018 Update by the Infectious Diseases Society of America and the American Society for Microbiology (2018 IDSA micro)

**Consensus Level:**

1. >90% agreement (Strongest Consensus or Unanimous) except for question number 3 where the consensus level is 85%.
2. The guideline does not provide level of evidence

**Prompt:**

**You are acting as a board-certified infectious disease consultant.**
 Your role is to evaluate clinical scenarios presented to you by a practicing physician who is exploring how large language models like you can support decision-making in infectious disease care.

You will be given:

A clinical case (including background, patient data, and key findings)

A multiple-choice question with four options (a–d), only **one of which is correct**

Your task is to:

**Identify the single best answer** based on the clinical scenario.

**Justify your choice** using expert-level clinical reasoning, as if explaining your thought process to a fellow physician.

Keep your explanation under **500 words**, and avoid repeating the question or answer choices.

If evidence is unclear, acknowledge uncertainty and explain your reasoning as you would in a real consult.

Please format your response like this:

makefile

CopyEdit

Answer: [a / b / c / d]

Justification:
[Your clinical reasoning – max 500 words]

**Patient Information**
Mr. Kessler, a 68-year-old man, presents with **progressively worsening right hip pain** over the past two months, particularly noticeable while walking. He also describes **intermittent low-grade fevers** and general fatigue. He denies trauma or recent infections.

He underwent **right total hip arthroplasty (THA) three years ago** for avascular necrosis. His medical history includes **rheumatoid arthritis** (on methotrexate and low-dose prednisone), **COPD (GOLD 2)**, and **depression**. He has never had a prosthetic joint infection before.

On exam, he has an **antalgic gait** and **pain with passive rotation of the right hip**, but no erythema or drainage is observed.

Initial workup:

1. **WBC**: 8.7 ×10⁹/L
2. **CRP**: 54 mg/L
3. **ESR**: 74 mm/hr
4. **Creatinine**: 1.0 mg/dL
5. **LFTs**: Normal

**Synovial fluid aspiration** yields 45 mL of cloudy fluid. **Culture grows *Enterococcus faecalis***, resistant to fluoroquinolones and aminoglycosides. Based on these findings, the patient undergoes **implant removal** and placement of an **antibiotic-loaded cement spacer**.

**Question 1:**

What is the most appropriate way to determine the route, dose, and duration of antibiotics in this case?

a) Administer a standard 6-week vancomycin course

b) Choose oral antibiotics with highest bone penetration especially with aggressive microbes

c) Select agent, dose, and duration based on the isolated organism

d) Tailor treatment to surgical preference

✅ **Correct Answer:** c**.**
📖 ICM 2018 PJI — Section 5.9, Question 4

The patient completes a **6-week course of IV ampicillin**, and inflammatory markers trend down (**CRP 6 mg/L**, **ESR 42 mm/hr**). He regains partial mobility with a walker. However, in week 7, he develops **recurrent hip pain**, and imaging shows **spacer dislocation with early bone lysis**. Repeat aspiration again isolates **E. faecalis** with the same resistance profile.

The team asks whether it’s appropriate to proceed with reimplantation.

**Question 2:**

What is the most appropriate next step?

a) Proceed directly with reimplantation as infection duration exceeds 6 weeks

b) Maintain current spacer and initiate oral suppressive therapy

c) Perform spacer exchange and repeat debridement due to persistent infection

d) Remove the spacer and delay reimplantation for another 12 weeks

✅ **Correct Answer:** c**.**
📖 ICM 2018 PJI — Section 5.4, Question 2

After the second **spacer exchange**, you and the orthopedic team begin planning for reimplantation. The fellow asks whether additional microbiological evaluation is necessary before and during the second-stage procedure.

**Question 3:**

Which of the following best reflects current recommendations regarding culture collection before and during the second stage of a two-stage exchange?

a) Inflammatory markers alone are sufficient to determine readiness for reimplantation

b) Preoperative aspiration should be performed in all cases, and intraoperative cultures are optional

c) The decision to perform preoperative aspiration should be based on clinical suspicion; multiple intraoperative fluid and tissue cultures should be obtained at reimplantation

d) Cultures should only be obtained if intraoperative purulence or tissue necrosis is observed

✅ **Correct Answer:** c**.**
📖 ICM 2018 PJI — Section 2.4, Question 4

After reimplantation, the orthopedic surgeon expresses concern about the possibility of **reinfection**, especially considering the organism involved and the patient's medical history.

**Question 4:**

What is the most appropriate response based on current evidence?

a) No, the patient has been adequately treated with a narrow-spectrum antimicrobial regimen

b) Yes, both a history of prior PJI in the operative joint and comorbid depression are recognized risk factors for reinfection

c) Yes, Enterococcus faecalis PJI is associated with a particularly high risk of recurrence

d) No, the patient received appropriate antimicrobial therapy and had negative cultures at reimplantation

✅ **Correct Answer:** b**.**
📖 ICM 2018 PJI — Section 1.1, Question 1

Six months later, while reviewing Mr. Kessler’s case with a new ID fellow, the topic of **organism-site associations** arises. The fellow asks whether certain pathogens are more commonly linked to infections in specific prosthetic joints.

**Question 5:**
Which of the following best reflects current evidence regarding the relationship between pathogen type and the site of prosthetic joint infection (PJI)?

a) No, the pathogens involved in hip and knee PJI are the same and show no specific distribution
b) The evidence is limited, but *Enterococcus* may be more commonly associated with hip PJIs, while *Streptococcus* may be more prevalent in knee PJIs
c) The evidence is limited, but culture-negative infections are more common in hip PJI, while *Pseudomonas* is more frequently seen in knee PJI
d) There is strong evidence that *Streptococcus* species are significantly more prevalent in knee PJIs

✅ **Correct Answer:** b**.**
📖 ICM 2018 PJI — Section 3, Question 3

**CLINICAL CASE 1: septic arthritis (SA)**

**Literature:**

Guideline for management of septic arthritis in native joints (SANJO)

A Guide to Utilization of the Microbiology Laboratory for Diagnosis of Infectious Diseases: 2018 Update by the Infectious Diseases Society of America and the American Society for Microbiology (2018 IDSA micro)

ACR Appropriateness Criteria Suspected septic arthritis (ACR septic arthritis)

**Level of consensus:**

1. GRADE: A/B/C/D 1 OR A/B 2,
2. The guideline does not provide level of evidence
3. Usually appropriate (7-9 points)

**Prompt:**

**You are acting as a board-certified infectious disease consultant.**
 Your role is to evaluate clinical scenarios presented to you by a practicing physician who is exploring how large language models like you can support decision-making in infectious disease care.

You will be given:

- A clinical case (including background, patient data, and key findings)
- A multiple-choice question with four options (a–d), only **one of which is correct**

Your task is to:

1. **Identify the single best answer** based on the clinical scenario.
2. **Justify your choice** using expert-level clinical reasoning, as if explaining your thought process to a fellow physician.
3. Keep your explanation under **500 words**, and avoid repeating the question or answer choices.
4. If evidence is unclear, acknowledge uncertainty and explain your reasoning as you would in a real consult.

Please format your response like this:

makefile

CopyEdit

Answer: [a / b / c / d]

Justification:
[Your clinical reasoning – max 500 words]

**Patient Information**

Mr. Jones, a 65-year-old male with **rheumatoid arthritis**, presents to clinic with **severe right knee pain** that began 5 days ago, followed by a **low-grade fever** for the past 24 hours. He reports difficulty bearing weight and significant joint stiffness. He denies trauma or recent injections.

On exam:

- T: 38.3°C
- BP: 124/70 mmHg
- HR: 88 bpm
- O₂ sat: 99% on room air
- The right knee is **swollen, warm, and tender**, with a **markedly reduced range of motion**.

Initial labs reveal:

1. **WBC**: 21 ×10⁹/L
2. **CRP**: 15 mg/L
3. **ESR**: 45 mm/hr

You are consulted by the ED team for evaluation of **possible septic arthritis**.

**Question 1:**

What is the appropriate diagnostic workup for a patient suspected of septic arthritis, **except for which of the following**?

a) Synovial fluid culture

b) At least two sets of blood cultures (one aerobic and one anaerobic for each set)

c) Synovial biopsy cultures and histopathological analysis

d) Urine cultures

✅ **Correct answer:** d
📚 *2018 IDSA micro*

You advise urgent aspiration and further testing. The orthopedic resident asks about the best initial imaging modality for workup.

**Question 2:**

In a patient suspected of septic arthritis, which initial imaging modality is most appropriate?

a) X-ray

b) MRI – to evaluate synovium and surrounding soft tissues early

c) CT scan – helpful to assess bone and periarticular structures

d) Ultrasound – often used to detect small effusions

✅ **Correct answer:** a
📚 *ACR septic arthritis: Variant 1*

Radiographs show mild joint space narrowing, but no definitive effusion or erosion. The fellow asks what should be done next if the plain films are unrevealing.

**Question 3:**

If initial radiographs are normal, what is the next appropriate imaging study, **except for which of the following**?

a) CT scan without contrast

b) MRI with contrast – useful for evaluating soft tissues and marrow involvement

c) Ultrasound – helpful to guide aspiration or detect effusion

d) MRI without contrast – can still detect marrow edema or joint inflammation

✅ **Correct answer:** a
📚 *ACR septic arthritis: Variant 2*

The patient is now in the ED with a visibly inflamed joint and rising inflammatory markers. The team wonders when to perform joint aspiration.

**Question 4:**

**When is the most appropriate timing for arthrocentesis of the knee?**

a) As soon as possible

b) Schedule after MRI to confirm joint space narrowing

c) Perform after ultrasound confirms sufficient fluid

d) Delay until empiric antibiotics are initiated to reduce sepsis risk

✅ **Correct answer:** a
📚 *SANJO Guideline: recommendation 1.1*

You perform a diagnostic arthrocentesis, yielding purulent synovial fluid with **WBC count of 70,000 cells/μL**, **90% neutrophils**, and **no crystals**. You obtain two sets of blood cultures and start empiric IV **cefazolin**.

**Question 5:**

What is the appropriate next step for this patient?

a) Consult orthopedic surgeons for surgical intervention

b) Admit to inpatient ward and continue empiric antibiotics without further intervention

c) Switch to oral cefadroxil and follow up in clinic if symptoms improve

d) Repeat joint aspiration after 48 hours to assess WBC trend

✅ **Correct answer:** a
📚 *SANJO Guideline: recommendation 3.1*

**CLINICAL CASE 2: septic arthritis (SA)**

**Literature:**

Guideline for management of septic arthritis in native joints (SANJO)

A Guide to Utilization of the Microbiology Laboratory for Diagnosis of Infectious Diseases: 2018 Update by the Infectious Diseases Society of America and the American Society for Microbiology (2018 IDSA micro)

ACR Appropriateness Criteria Suspected septic arthritis (ACR septic arthritis)

**Level of consensus:**

1. A/B/C/D 1 OR A/B 2,
2. The guideline does not provide level of evidence
3. Usually appropriate (7-9 points)

**Prompt:**

**You are acting as a board-certified infectious disease consultant.**
 Your role is to evaluate clinical scenarios presented to you by a practicing physician who is exploring how large language models like you can support decision-making in infectious disease care.

You will be given:

A clinical case (including background, patient data, and key findings)

A multiple-choice question with four options (a–d), only **one of which is correct**

Your task is to:

**Identify the single best answer** based on the clinical scenario.

**Justify your choice** using expert-level clinical reasoning, as if explaining your thought process to a fellow physician.

Keep your explanation under **500 words**, and avoid repeating the question or answer choices.

If evidence is unclear, acknowledge uncertainty and explain your reasoning as you would in a real consult.

Please format your response like this:

makefile

CopyEdit

Answer: [a / b / c / d]

Justification:
[Your clinical reasoning – max 500 words]

**Patient Information**

**Mrs. Brenda Morgan**
 **Age: 72**
 **Medical History:** Type 2 Diabetes, Hypertension, Right Total Knee Arthroplasty (6 months ago)
 **Medications:** Metformin, Amlodipine, Aspirin

Mrs. Morgan is brought to the emergency department by her daughter, who’s concerned about her mother’s increasing difficulty walking. The patient reports **3 days of right knee swelling**, pain with weight-bearing, and **low-grade fever** that started yesterday evening. She denies recent trauma, sick contacts, or known injuries.

She’s been **missing her diabetes meds intermittently** and notes mild urinary frequency over the last week, but urinalysis at triage is unremarkable. No history of rash, chest pain, or cough.

She also reports **low-grade fever (T 37.8°C)** and **general malaise**. There’s no history of recent trauma, skin infections, travel, or rash.

On exam:

- Moderate **effusion**, warmth, tenderness, and **limited range of motion** in the right knee.
- Vitals are stable, and she is alert but appears uncomfortable with movement.

The ED physician suspects either a **flare of osteoarthritis** or **early infection**, and calls you, the ID consultant, for input.



**Question 1:**
What clinical parameters do you check for excluding septic arthritis?

a) Fever must be present to consider septic arthritis

b) If WBC is <10,000 and the patient is afebrile, infection is unlikely

c) No clinical parameter alone can exclude or confirm SA

d) In the absence of prior joint pain, septic arthritis can be ruled out

✅ **Correct answer:** c
📚 *SANJO 2023: recommendation 1.1*



Initial labs: WBC: 11.2 x10⁹/L, CRP: 88 mg/L, ESR: 77 mm/h, Plain radiographs show a **well-positioned prosthesis**, no fracture or hardware loosening. The ED team considers sending the patient home with pain control and outpatient imaging, but you're concerned based on the lab trends and exam.

You recommend further evaluation.



**Question 2:**
What is the most appropriate imaging strategy at this stage?

a) Proceed directly to MRI to assess deep joint tissue involvement

b) Bone scan is more sensitive than ultrasound for detecting prosthetic infection

c) Ultrasound to assess for joint effusion

d) Repeat plain films in 24 hours to look for evolving erosions

✅ **Correct answer:** c
📚 *ACR septic arthritis: variant 2*



Ultrasound reveals a **moderate effusion**, and orthopedics is paged for joint aspiration. A junior resident performs the tap but draws no fluid. They suspect the effusion was overestimated.



**Question 3:**
What do you recommend in cases of dry aspiration?

a) Inject sterile saline to increase diagnostic yield in low-volume effusions

b) Proceed directly to surgical washout to avoid delays in management

c) Use image-guided aspiration in case of dry tap

d) Assume the lack of fluid confirms absence of effusion

✅ **Correct answer:** c
📚 *SANJO 2023: recommendation 2.2*



A fluoroscopy-guided aspiration yields **14 mL of cloudy synovial fluid**. Gram stain is negative.

1. **Synovial WBC:** 83,000/mm³
2. **PMNs:** 94%
3. Culture: pending

The lab asks how they should handle the sample for optimal microbiological yield.



**Question 4:**
How should synovial fluid be processed to improve culture yield?

a) Inoculate directly onto agar plates to prevent overgrowth of contaminants

b) Send for PCR first, as it’s faster than culture

c) Order urinalysis to rule out false positives from systemic inflammation

d) Inoculate fluid into blood culture bottles

✅ **Correct answer:** d
📚 *IDSA 2018 MICRO*



That evening, the patient develops **new hypotension (BP 89/54)**, **tachycardia (HR 110)**, and **fever of 38.9°C**. She becomes **confused**, and lactate returns at 3.8 mmol/L.

The nurse asks whether to wait for orthopedic input before initiating treatment.



**Question 5:**
**What is your recommendation for antibiotic initiation in septic shock?**

a) Delay antibiotics until Gram stain and cultures are finalized

b) Take the patient to the OR for washout before starting antibiotics

c) Administer empiric antibiotics immediately

d) Consult orthopedics and await surgical evaluation before initiating treatment

✅ **Correct answer:** c
📚 *SANJO 2023: recommendation 4.1*

**CLINICAL CASE 3: septic arthritis (SA)**

**Literature:**

Guideline for management of septic arthritis in native joints (SANJO)

A Guide to Utilization of the Microbiology Laboratory for Diagnosis of Infectious Diseases: 2018 Update by the Infectious Diseases Society of America and the American Society for Microbiology (2018 IDSA micro)

ACR Appropriateness Criteria Suspected septic arthritis (ACR septic arthritis)

**Level of consensus:**

1. A/B/C/D 1 OR A/B 2,
2. The guideline does not provide level of evidence
3. Usually appropriate (7-9 points)

**Prompt:**

**You are acting as a board-certified infectious disease consultant.**
 Your role is to evaluate clinical scenarios presented to you by a practicing physician who is exploring how large language models like you can support decision-making in infectious disease care.

You will be given:

- A clinical case (including background, patient data, and key findings)
- A multiple-choice question with four options (a–d), only **one of which is correct**

Your task is to:

1. **Identify the single best answer** based on the clinical scenario.
2. **Justify your choice** using expert-level clinical reasoning, as if explaining your thought process to a fellow physician.
3. Keep your explanation under **500 words**, and avoid repeating the question or answer choices.
4. If evidence is unclear, acknowledge uncertainty and explain your reasoning as you would in a real consult.

Please format your response like this:

makefile

CopyEdit

Answer: [a / b / c / d]

Justification:
[Your clinical reasoning – max 500 words]



**Patient Information**

Mr. Dunne, a 67-year-old retired bus driver, presents to the ED with **sudden onset of left knee pain and swelling** that began the day before. He says the pain escalated overnight and now makes it difficult to stand. He reports **no recent trauma, surgery, or travel**, and hasn't noticed any insect bites, rashes, or systemic illness.

He has a history of **type 2 diabetes (on insulin)**, **gout (managed with allopurinol)**, and **chronic kidney disease stage 3**, for which he's been taking **losartan**. His blood sugars have been “on the higher side” lately, and he mentions he had a gout flare about two months ago, which resolved without hospital care.

On exam:

- T: 38.2°C
- BP: 132/78
- HR: 86 bpm
- Left knee: tense effusion, warmth, erythema, and limited flexion. No overlying skin lesions.

The triage team initially suspects a **gout flare** and gives IV ketorolac, but when the fever persists and swelling worsens, you are called for infectious disease consultation.



**Question 1:**
What do you recommend regarding synovial fluid analysis in suspected SA with a history of gout?

a) In the presence of gout history, testing for crystals alone is sufficient to confirm diagnosis

b) Gram stain is the most sensitive test and should be prioritized over cell count or culture

c) Always evaluate for culture, WBC, and crystals — in that order

d) If crystals are found, no further testing is necessary, as they exclude superinfection

✅ **Correct answer:** c
📚 *SANJO Guideline: recommendation 1.3*



A diagnostic aspiration is performed, yielding **cloudy yellow fluid**. Lab results show:

1. **Synovial WBC:** 68,000/mm³
2. **Neutrophils:** 93%
3. **Crystals:** Positive for monosodium urate
4. **Gram stain:** Negative
5. No prosthetic material in situ

The ED resident feels reassured by the presence of crystals and suggests managing this as gout.

**Question 2:**
How should WBC count in synovial fluid be interpreted?

a) A WBC count >50,000/mm³ is diagnostic of septic arthritis

b) <25,000 WBC/mm³ reliably rules out septic arthritis in the absence of prosthetic joints

c) WBC count must be correlated clinically; thresholds are not absolute

d) If crystals are present, cell count interpretation is not clinically meaningful

✅ **Correct answer:** c
📚 *SANJO GUIDELINE: recommendation 1.4*



Plain films of the knee are unremarkable. Due to persistent symptoms and the ambiguous fluid findings, a fellow asks whether MRI would help in this context.



**Question 3:**
What is your position on using MRI in difficult or deep-seated joint infections?

a) Not necessary unless patient is febrile
 b) Useful when clinical suspicion is high, especially for sacroiliac joint or osteomyelitis
 c) Reserved only for prosthetic joints
 d) Contraindicated if aspiration was unsuccessful

✅ **Correct answer:** b
📚 *SANJO GUIDELINE: recommendation 1.5*



MRI of the knee is performed and shows joint effusion but no signs of adjacent osteomyelitis. Cultures remain negative after 72 hours. Despite ongoing symptoms, a second aspiration isn’t attempted. Ortho hesitates to re-intervene, citing the presence of urate crystals.

You are now asked how to proceed diagnostically.



**Question 4:**
What is the next recommended diagnostic step if synovial fluid cultures are negative but suspicion remains?

a) No further testing needed
 b) Repeat synovial fluid WBC
 c) Start antifungals empirically
 d) Perform synovial biopsy

✅ **Correct answer:** d
📚 *IDSA 2018 MICRO*



Ortho proceeds with **arthroscopic washout and debridement**, based on worsening pain and persistent inflammatory markers. Intraoperative samples confirm neutrophilic inflammation. Cultures remain negative. Based on local resistance data, **empiric therapy is de-escalated to cefazolin**.



**Question 5:**
What do you think regarding the choice of surgical approach?

a) Open surgical debridement is preferred over arthroscopy in all native joint infections

b) Arthroscopy is preferred in early-stage infection (Gächter I–III)

c) Arthroscopy is no longer considered adequate in septic arthritis

d) Repeated aspiration is typically more effective than surgery for drainage and infection control

✅ **Correct answer:** b
📚 *SANJO GUIDELINE: recommendation 3.1*

**CLINICAL CASE 4: septic arthritis (SA)**

**Literature:**

Guideline for management of septic arthritis in native joints (SANJO)

A Guide to Utilization of the Microbiology Laboratory for Diagnosis of Infectious Diseases: 2018 Update by the Infectious Diseases Society of America and the American Society for Microbiology (2018 IDSA micro)

ACR Appropriateness Criteria Suspected septic arthritis (ACR septic arthritis)

**Level of consensus:**

1. A/B/C/D 1 OR A/B 2,
2. The guideline does not provide level of evidence
3. Usually appropriate (7-9 points)

**Prompt:**

**You are acting as a board-certified infectious disease consultant.**
 Your role is to evaluate clinical scenarios presented to you by a practicing physician who is exploring how large language models like you can support decision-making in infectious disease care.

You will be given:

A clinical case (including background, patient data, and key findings)

A multiple-choice question with four options (a–d), only **one of which is correct**

Your task is to:

**Identify the single best answer** based on the clinical scenario.

**Justify your choice** using expert-level clinical reasoning, as if explaining your thought process to a fellow physician.

Keep your explanation under **500 words**, and avoid repeating the question or answer choices.

If evidence is unclear, acknowledge uncertainty and explain your reasoning as you would in a real consult.

Please format your response like this:

makefile

CopyEdit

Answer: [a / b / c / d]

Justification:
[Your clinical reasoning – max 500 words]



**Patient Information**

Ms. Nazari is a 59-year-old woman with **rheumatoid arthritis**, maintained on **methotrexate and low-dose prednisone**, who presents with **6 days of worsening right shoulder pain and limited movement**. She says the discomfort began after a long drive and has slowly progressed. There’s no history of trauma, injection, or overuse.

She describes **mild fevers at home**, which she attributed to her “RA flaring up.” No recent sick contacts or upper respiratory symptoms.

Past medical history also includes **stage 2 chronic kidney disease**, and she’s been taking **omeprazole for years** for presumed GERD. Her prednisone dose is 5 mg daily, and she has been adherent.

On examination:

1. **T**: 37.5°C
2. Right shoulder: visible swelling, warmth, and significant limitation with both passive and active movement. No overlying erythema or drainage.
3. She appears non-toxic, with stable vitals and intact mentation.

The ED team leans toward a **rheumatoid flare** given her background, but they call you — the ID consultant — for a second opinion.



**Question 1:**
Based on current recommendations, what is the most appropriate interpretation of this clinical scenario?

a) The presentation is consistent with a rheumatoid flare; in the absence of systemic signs, no immediate workup is needed

b) Septic arthritis should remain on the differential; no clinical signs or symptoms alone can reliably confirm or exclude the diagnosis

c) Septic arthritis is unlikely without high-grade fever and purulent joint drainage

d) In immunosuppressed patients, low-grade fever usually reflects a flare rather than infection if synovial fluid is non-inflammatory

✅ **Correct answer:** b
📚 *SANJO GUIDELINE: recommendation 1.1*



Plain radiographs of the shoulder are unremarkable. A bedside aspiration attempt fails due to **deep joint anatomy and poor visualization**, so a fluoroscopy-guided aspiration is arranged.

The next morning, radiology successfully aspirates **9 mL of cloudy yellow synovial fluid**. The fluid is sent to the lab. At noon, you receive a partial update:

“So far we have a **WBC count around 21,000** with **92% neutrophils**. Gram stain is still negative. Culture’s in progress.”

The junior resident who assisted with the bedside tap earlier asks whether injecting **saline into the joint before aspiration** could help increase the diagnostic yield, especially in small effusions.

.

**Question 2:**

Which of the following best reflects current recommendations regarding aspiration technique in suspected septic arthritis?

a) Saline may be used in small joints to ensure sufficient sample volume for microbiology

b) Injection of saline is discouraged, as it may dilute the synovial fluid and compromise culture accuracy; image guidance should be used when access is difficult

c) Saline can be used safely unless the patient is febrile or has elevated inflammatory markers

d) Saline can be used for aspiration only if prior antibiotics have been administered, to help rehydrate the joint space.

✅ **Correct answer:** b
📚 *SANJO GUIDELINE: recommendation 2.2*

A few hours later, the lab calls back with a more complete profile.

“Gram stain remains negative. Crystals not seen. We’re holding off on culture comment for now.”

The synovial WBC remains at **21,000/mm³**, which leads the orthopedics resident to suggest waiting for cultures before proceeding with any intervention. She argues that the borderline value and negative Gram stain support a more conservative approach.



**Question 3:**
How should synovial WBC <25,000/mm³ can be interpreted?

a) A WBC count <25,000 virtually excludes septic arthritis in immunocompetent patients

b) Synovial fluid in viral arthritis typically shows WBC <10,000 with mixed cell populations

c) It reduces probability but does not exclude infection

d) Low synovial WBC in the absence of crystals points to reactive arthritis

✅ **Correct answer:** c
📚 *SANJO GUIDELINE: recommendation 1.4*



Despite some hesitation, orthopedics proceeds with **arthroscopic washout** the next morning, citing persistent symptoms and elevated inflammatory markers. There’s debate during pre-op rounds about whether repeated aspiration should have been attempted first.



**Question 4:**
What do you recommend regarding surgical drainage in native joint septic arthritis?

a) Repeated aspiration should always be attempted before surgery to avoid unnecessary intervention

b) Surgical drainage is primarily indicated for prosthetic joints or failure of medical therapy

c) Surgical drainage is standard, especially for large joints

d) Drainage should be deferred unless Gram stain is positive or cultures grow bacteria

✅ **Correct answer:** c
📚 *SANJO GUIDELINE: recommendation 3.1*



The surgery goes smoothly, and the intra-op note mentions **cloudy joint fluid**, but no gross purulence. Cultures are still pending. The surgical team highlights their use of **strict sterile technique**, including sterile draping and equipment change after prepping.

Later that day, a student asks you about proper infection control protocols during joint aspiration.



**Question 5:**
What do you recommend regarding aspiration technique?

a) For superficial joints, alcohol prep alone is adequate when aspiration is done in a procedural area

b) Maintain strict aseptic technique to avoid contamination

c) Mask and gown are optional unless aspiration is performed in the OR

d) Local anesthetic can be injected directly into the joint to minimize patient discomfort

✅ **Correct answer:** b
📚 *SANJO GUIDELINE: recommendation 2.2*

**CLINICAL CASE 5: septic arthritis (SA)**

**Literature:**

Guideline for management of septic arthritis in native joints (SANJO)

A Guide to Utilization of the Microbiology Laboratory for Diagnosis of Infectious Diseases: 2018 Update by the Infectious Diseases Society of America and the American Society for Microbiology (2018 IDSA micro)

ACR Appropriateness Criteria Suspected septic arthritis (ACR septic arthritis)

**Level of consensus:**

1. A/B/C/D 1 OR A/B 2,
2. The guideline does not provide level of evidence
3. Usually appropriate (7-9 points)

**Prompt:**

**You are acting as a board-certified infectious disease consultant.**
 Your role is to evaluate clinical scenarios presented to you by a practicing physician who is exploring how large language models like you can support decision-making in infectious disease care.

You will be given:

- A clinical case (including background, patient data, and key findings)
- A multiple-choice question with four options (a–d), only **one of which is correct**

Your task is to:

1. **Identify the single best answer** based on the clinical scenario.
2. **Justify your choice** using expert-level clinical reasoning, as if explaining your thought process to a fellow physician.
3. Keep your explanation under **500 words**, and avoid repeating the question or answer choices.
4. If evidence is unclear, acknowledge uncertainty and explain your reasoning as you would in a real consult.

Please format your response like this:

makefile

CopyEdit

Answer: [a / b / c / d]

Justification:
[Your clinical reasoning – max 500 words]



**Patient Information**

Mr. Okoro is a 42-year-old civil engineer, originally from Nigeria, who presents with a **3-month history of progressive right hip pain**. He says the discomfort started vaguely — mostly when walking long distances — but has steadily worsened to the point that he's now avoiding stairs. He also reports **unintentional weight loss (5 kg)** and **evening fevers** over the past few weeks, which he’s been attributing to stress.

He denies trauma, recent travel, or sexual activity. No known sick contacts. When asked directly, he shares that he had a **positive TB skin test years ago**, but **never received treatment**, and says, *“They told me it wasn’t active, so I didn’t worry about it.”*

No cough or respiratory symptoms. He is not on any medications.

On exam:

- Appears thin but non-toxic
- T: 37.6°C
- The right hip has **moderate limitation in range of motion**, particularly internal rotation. No visible swelling, erythema, or warmth.
- Lungs are clear. There’s no spinal tenderness.

Labs trickle in over the morning:

- **CRP**: 42 mg/L
- **ESR**: 82 mm/hr
- **HIV**: negative
- **CBC**: unremarkable

Chest X-ray shows a **calcified granuloma in the right upper lobe**. Plain hip films reveal **subtle joint space narrowing**, but no clear erosion.



**Question 1:**

In the suspect of TB arthritis, what is your position on early surgical intervention?

a) Early arthroscopic debridement is preferred to prevent irreversible joint damage, regardless of disease phase

b) Surgery should be avoided during the active TB phase unless absolutely necessary

c) Synovial biopsy is always required surgically to confirm TB diagnosis

d) TB arthritis should be managed surgically like pyogenic arthritis when effusion is present

✅ **Correct answer:** b
📚 *SANJO GUIDELINE: recommendation 6.1*



An ultrasound-guided aspiration is performed. The synovial fluid looks **slightly turbid but not purulent**. The aspirate is sent for analysis.

- The initial report shows **WBC: 18,000/mm³** with a **lymphocytic predominance**.
- **AFB smear**: negative.
- **PCR and cultures**: pending.
   You ask the lab to hold part of the sample for NAAT and fungal culture just in case.



**Question 2:**
What is the recommended antimicrobial regimen for TB arthritis?

a) Two-drug oral therapy for 6 months
b) Isoniazid + rifampin for 4 months
c) 2 months of 4-drug therapy followed by continuation phase
d) Fluoroquinolone monotherapy for 12 weeks

✅ **Correct answer:** c
📚 *SANJO GUIDELINE: recommendation 6.1*

Later that day, a medical student asks about **treatment duration**, pointing out that *“most TB treatments last 6 months, right?”* The fellow hesitates and looks to you for clarification.

**Question 3:**
What is the current recommendation regarding the duration of antimicrobial therapy for osteoarticular tuberculosis caused by drug-susceptible strains?

a) A standard regimen of 2 months of 4-drug therapy followed by 2 additional months of continuation phase is sufficient in most cases
 b) Some guidelines suggest a minimum of 2 months of treatment for drug-resistant tuberculosis
 c) Some guidelines recommend a maximum of 6 months of therapy for drug-susceptible tuberculosis, regardless of clinical response
 d) Some guidelines suggest a minimum of 6 months of therapy for drug-susceptible tuberculosis, though many experts favor extended durations of 9 to 12 months in osteoarticular cases

✅ **Correct answer:** d
📚 *SANJO GUIDELINE: recommendation 6.1*

At this point, you're coordinating treatment and discussing who should follow him long-term. Orthopedics believes they’ve “ruled out the need for surgery,” while Pulmonology isn’t sure whether they should be involved since there’s no active pulmonary TB.

**Question 4:**
What is your answer regarding TB arthritis management oversight?

a) Orthopedic teams can oversee treatment as long as there’s no evidence of systemic TB

b) Pulmonology follow-up is only needed for patients with active lung disease

c) Infectious disease expert should supervise TB treatment

d) Stable cases can be monitored by primary care after initiating therapy

✅ **Correct answer:** c
📚 *SANJO GUIDELINE: recommendation 6.1*



Due to diagnostic uncertainty and concern for bone involvement, you order an **MRI of the right hip**, which shows:

1. Moderate **synovial thickening**
2. **Joint erosion**
3. Early involvement of the adjacent acetabulum

The radiologist recommends contrast but says *“findings are pretty clear either way.”*



**Question 5:**
According to ACR guidelines, what is the role of MRI in suspected osteoarticular TB?

a) MRI is only needed if X-rays are abnormal
b) MRI with or without contrast is appropriate to assess osteomyelitis or deep joint involvement
c) MRI is not useful for infections
d) Bone scan is preferred for joint infection

✅ **Correct answer:** b
📚 *ACR septic arthritis: Variant 3*



During rounds, a junior physician remarks, *“It’s weird — the fluid didn’t look infected. It wasn’t that cloudy and was kind of thick.”* You use this as a teaching moment.



**Question 6:**
What characteristics typically has synovial fluid in SA?

a) Thick, viscous, lymphocyte-rich fluid is typical of chronic non-infectious arthritis

b) Most SA cases have purulent, low-viscosity fluid with neutrophilic predominance

c) Crystals must be identified to exclude septic arthritis

d) Fluid color and odor are more predictive than cell count or Gram stain

✅ **Correct answer:** b
📚 *IDSA 2018 MICRO*

**CLINICAL CASE 6: septic arthritis (SA)**

**Literature:**

Guideline for management of septic arthritis in native joints (SANJO)

A Guide to Utilization of the Microbiology Laboratory for Diagnosis of Infectious Diseases: 2018 Update by the Infectious Diseases Society of America and the American Society for Microbiology (2018 IDSA micro)

ACR Appropriateness Criteria Suspected septic arthritis (ACR septic arthritis)

**Level of consensus:**

1. A/B/C/D 1 OR A/B 2,
2. The guideline does not provide level of evidence
3. Usually appropriate (7-9 points)

**Prompt:**

**You are acting as a board-certified infectious disease consultant.**
 Your role is to evaluate clinical scenarios presented to you by a practicing physician who is exploring how large language models like you can support decision-making in infectious disease care.

You will be given:

- A clinical case (including background, patient data, and key findings)
- A multiple-choice question with four options (a–d), only **one of which is correct**

Your task is to:

1. **Identify the single best answer** based on the clinical scenario.
2. **Justify your choice** using expert-level clinical reasoning, as if explaining your thought process to a fellow physician.
3. Keep your explanation under **500 words**, and avoid repeating the question or answer choices.
4. If evidence is unclear, acknowledge uncertainty and explain your reasoning as you would in a real consult.

Please format your response like this:

makefile

CopyEdit

Answer: [a / b / c / d]

Justification:
[Your clinical reasoning – max 500 words]



**Patient Information**

Mr. Rivera is a healthy 27-year-old recreational soccer player who presents with **increasing right knee pain and stiffness** over the past 5 days. He underwent an **uncomplicated ACL reconstruction** 3 weeks ago and had been recovering well until this new onset of symptoms. He denies any fevers, chills, or wound drainage.

He’s been taking **ibuprofen as needed** but hasn’t used opioids or antibiotics since discharge. No prior knee issues. He lives alone and works remotely, with no recent sick contacts.

On exam:

- He is afebrile and appears well
- Right knee shows **trace effusion**, **reduced ROM**, and **mild warmth**, but no erythema or surgical site drainage
- Surgical portals are healing appropriately

The ED team is on the fence: *“Could this just be post-op inflammation?”* Orthopedics asks you, the ID consultant, whether this could be early infection despite the mild presentation.



**Question 1:**
What is correct regarding early signs of septic arthritis after ACL reconstruction?

a) Pain and stiffness are common post-op findings and do not raise concern unless accompanied by persistent fever
 b) Purulent discharge is the most reliable early sign and usually precedes systemic symptoms
 c) Pain, stiffness, systemic symptoms, or purulence should raise suspicion
 d) Post-operative effusion and warmth are expected and do not warrant further workup unless CRP exceeds 100 mg/L

✅ **Correct answer:** c
📚 *SANJO GUIDELINE: recommendation 5.1*



Joint aspiration is performed with ultrasound guidance. The fluid is described as **slightly turbid**, and is placed into a sterile cup and sent to the lab. Three hours later, the lab calls:

“We’ve got a **WBC of 56,000/mm³**, mostly neutrophils. Gram stain still pending.”

A resident mentions they didn’t inoculate blood culture bottles at bedside — *“We just sent the fluid in a sterile cup like usual.”*



**Question 2:**
What is the optimal synovial fluid culture technique?

a) Plate the sample directly onto solid media in the laboratory to minimize contamination risk
 b) Freeze the specimen if immediate processing is not possible, to preserve organism viability
 c) Inoculate synovial fluid directly into aerobic and anaerobic blood culture bottles at the bedside and send promptly to the lab
 d) Standard sterile container submission is sufficient in most cases, provided transport to the lab is timely

✅ **Correct answer:** c
📚 *IDSA 2018 MICRO*



Orthopedics proceeds with **arthroscopic debridement within 24 hours**. Despite the high cell count, initial synovial cultures show **no growth after 72 hours**, and the clinical team questions the diagnosis.



**Question 3:**
What should be done if synovial fluid cultures are negative in a suspected case of SA?

a) If symptoms improve and the initial aspiration was purulent, empiric antibiotics may be sufficient without further investigation
 b) Synovial biopsy should be considered
 c) Hold antibiotics and monitor clinical course; repeat aspiration can be done electively
 d) If Gram stain and cultures are both negative, a non-infectious cause should be presumed and treatment discontinued

.

✅ **Correct answer:** b
📚 *IDSA 2018 MICRO*



Post-op imaging (MRI and CT) shows **no signs of osteomyelitis or abscess**. The ortho team asks whether any further surgical intervention is required or if the initial debridement was enough.



**Question 4:**
What is the surgical recommendation for suspected ACL-R infection?

a) If the joint is stable and labs are equivocal, repeat aspiration should be prioritized over surgery
b) Surgical intervention is best delayed until cultures confirm infection or imaging shows joint erosion
c) Prompt surgical debridement is recommended upon suspicion
d) Hardware can often be preserved; graft removal alone is typically sufficient in early infections

✅ **Correct answer:** c
📚 *SANJO GUIDELINE: recommendation 5.2*



Intraoperatively, the ortho resident prepares to perform a second aspiration “for confirmation.” He scrubs in and applies chlorhexidine, then immediately reaches for the needle. You pause him before he inserts it and ask, “Did you let that dry?”

He looks up and asks you: *“Do we really need to wait for drying if we’re already in the OR?”*



**Question 5:**
 What is the most appropriate response?

a) No, if the procedure is performed in the OR under sterile conditions, drying time is irrelevant
b) Yes, the antiseptic must be allowed to dry completely, as inserting the needle prematurely can increase the risk of contaminating both the joint and the sample
c) No, chlorhexidine works immediately on contact, so drying time is a matter of operator preference
d) Yes, but only when aspirating native joints; prosthetic joints are less vulnerable to contamination in this setting

✅ **Correct answer:** b
📚 *SANJO GUIDELINE: recommendation 2.2*

**CLINICAL CASE 1: Vertebral osteomyelitis (VO)**

**Literature:**

1. 2015 Infectious Diseases Society of America (IDSA) Clinical Practice Guidelines for the Diagnosis and Treatment of Native Vertebral Osteomyelitis in Adults (2015 IDSA NVO)
2. A Guide to Utilization of the Microbiology Laboratory for Diagnosis of Infectious Diseases: 2018 Update by the Infectious Diseases Society of America and the American Society for Microbiology (2018 IDSA micro)
3. ACR Appropriateness Criteria Suspected Spine Infection (ACR spine infection)

**Level of consensus:**

1. GRADE: Strong recommendation (> 90%)
2. The guideline does not provide level of evidence
3. Usually appropriate (7-9 points) and May be appropriate (4-6 points)

**Prompt:**

**You are acting as a board-certified infectious disease consultant.**
 Your role is to evaluate clinical scenarios presented to you by a practicing physician who is exploring how large language models like you can support decision-making in infectious disease care.

You will be given:

- A clinical case (including background, patient data, and key findings)
- A multiple-choice question with four options (a–d), only **one of which is correct**

Your task is to:

1. **Identify the single best answer** based on the clinical scenario.
2. **Justify your choice** using expert-level clinical reasoning, as if explaining your thought process to a fellow physician.
3. Keep your explanation under **500 words**, and avoid repeating the question or answer choices.
4. If evidence is unclear, acknowledge uncertainty and explain your reasoning as you would in a real consult.

Please format your response like this:

makefile

CopyEdit

Answer: [a / b / c / d]

Justification:
[Your clinical reasoning – max 500 words]

**Consult Request:**
 A 60-year-old woman with new-onset fever and worsening lower back pain is referred to Infectious Diseases to evaluate for possible vertebral osteomyelitis following a recent corticosteroid injection.

### **Clinical Course:**

Miss Laura Smith, a 60-year-old with hypertension and chronic atopic dermatitis, was seen in the ED for escalating back pain that began to worsen two days ago. She describes the pain as deep and sharp, radiating slightly into her left flank, different in character from the degenerative disc discomfort she’s managed conservatively for years.

Notably, she had undergone a **lumbar corticosteroid injection** at a private pain clinic about a week prior. Since then, she has developed low-grade fevers and increased tenderness over the lower lumbar spine.

On arrival, she appeared flushed but oriented. Her vitals showed a temperature of 38.4°C and a heart rate of 80 bpm. Blood pressure and oxygenation were normal. Physical exam revealed **midline tenderness over L4–L5**, without any spinal deformity or neurologic deficits. There was no paraspinal swelling, fluctuance, or drainage.

Her white count was mildly elevated at 15.6 x10⁹/L, and CRP was 20 mg/L. Given the concern for discitis-osteomyelitis, the admitting team wanted to know the appropriate next diagnostic step.

### **Question 1**

What is the most appropriate **next diagnostic step** in this patient?

a) CT lumbar
b) CT lumbar + blood cultures
c) MRI lumbar
d) MRI lumbar + blood cultures
e) X-ray lumbar
f) X-ray lumbar + blood cultures

✅ **Correct answer:** b
📚 *2015 IDSA NVO:* RECOMMENDATIONS FOR CLINICAL DIAGNOSTICS, question 2

📚 *ACR spine infection: Variant 1*

### **Imaging:**

CT reveals **endplate irregularity at L5–S1**, suspicious for early osteomyelitis. MRI with STIR confirms **hyperintensity of L5 and S1 vertebral bodies**, consistent with **early discitis-osteomyelitis**.

Empiric therapy with **vancomycin and cefepime** is initiated. Blood cultures remain negative after 12 hours. Neurosurgery is consulted and raises the possibility of **CT-guided vertebral aspiration**.

### **Question 2**

What is the appropriate recommendation regarding aspiration?

a) Yes, but delay antibiotics for 48 hours
 b) Yes, proceed immediately
 c) No, change cefepime to meropenem
 d) No, cultures are likely false-negative due to prior antibiotics

✅ **Correct answer:** b
📚 *2015 IDSA NVO:* RECOMMENDATIONS FOR CLINICAL DIAGNOSTICS, question 3

📚 *2018 IDSA micro*

### **Culture Results:**

Vertebral aspiration yields **Staphylococcus lugdunensis**, pan-sensitive. Antimicrobial therapy is narrowed to **IV cefazolin**. The patient defervesces by day 3. Repeat physical exam shows no spinal tenderness or neurologic symptoms.

Internal medicine requests guidance on whether to involve orthopedics for surgical intervention.

### **Question 3**

What is the appropriate recommendation regarding surgery?

a) Yes, surgery is required for all cases of native vertebral osteomyelitis
 b) No, patient is stable and has no neurologic deficits
 c) Yes, because *S. lugdunensis* is isolated
 d) No, because *S. lugdunensis* infections are benign

✅ **Correct answer:** b
📚 2015 IDSA NVO: RECOMMENDATIONS FOR CLINICAL THERAPY, question 2

### **Treatment Response:**

The patient completes **6 weeks of cefazolin** without complications. She reports gradual improvement in pain and mobility. Repeat labs show normalization of WBC and CRP.

### **Question 4**

What is the **recommended duration** of antimicrobial therapy?

a) 2 weeks
 b) 4 weeks
 c) 6 weeks
 d) 12 weeks

✅ **Correct answer:** c
📚 2015 IDSA NVO: RECOMMENDATIONS FOR CLINICAL THERAPY, question 1

### **Follow-Up Visit (2 Weeks Post-Therapy):**

The patient is afebrile, walking independently, and reports no residual back pain. Labs remain normal. No neurologic changes.

### **Question 5**

What is the most appropriate next step?

a) Repeat MRI lumbar spine
b) Obtain PET-CT
c) Repeat blood cultures
d) No further work-up needed

✅ **Correct answer:** d
📚 *20*15 IDSA NVO: RECOMMENDATIONS FOR CLINICAL FOLLOW-UP, question 2

**CLINICAL CASE 2: Vertebral osteomyelitis (VO)**

**Literature:**

1. 2015 Infectious Diseases Society of America (IDSA) Clinical Practice Guidelines for the Diagnosis and Treatment of Native Vertebral Osteomyelitis in Adults (2015 IDSA NVO)
2. A Guide to Utilization of the Microbiology Laboratory for Diagnosis of Infectious Diseases: 2018 Update by the Infectious Diseases Society of America and the American Society for Microbiology (2018 IDSA micro)
3. ACR Appropriateness Criteria Suspected Spine Infection (ACR spine infection)

**Level of consensus:**

1. Strong recommendation (> 90%)
2. The guideline does not provide level of evidence
3. Usually appropriate (7-9 points) and May be appropriate (4-6 points)

**Prompt:**

**You are acting as a board-certified infectious disease consultant.**
 Your role is to evaluate clinical scenarios presented to you by a practicing physician who is exploring how large language models like you can support decision-making in infectious disease care.

You will be given:

- A clinical case (including background, patient data, and key findings)
- A multiple-choice question with four options (a–d), only **one of which is correct**

Your task is to:

1. **Identify the single best answer** based on the clinical scenario.
2. **Justify your choice** using expert-level clinical reasoning, as if explaining your thought process to a fellow physician.
3. Keep your explanation under **500 words**, and avoid repeating the question or answer choices.
4. If evidence is unclear, acknowledge uncertainty and explain your reasoning as you would in a real consult.

Please format your response like this:

makefile

CopyEdit

Answer: [a / b / c / d]

Justification:
[Your clinical reasoning – max 500 words]

**Consult Request:**
 A 64-year-old man with worsening back pain and elevated inflammatory markers, now with imaging findings consistent with vertebral osteomyelitis. ID is consulted to confirm diagnosis, guide microbiologic workup, and help determine next steps.

### **Clinical Course:**

Mr. Robert Martin, a retired schoolteacher, presented to the ED with **two weeks of gradually worsening low back pain**. He reported profound fatigue and night sweats but denied any focal neurologic symptoms, urinary complaints, or recent trauma. His past medical history includes **type 2 diabetes mellitus**, longstanding **degenerative disc disease**, and a history of **MSSA bacteremia** about three years ago. He also has GERD, for which he takes omeprazole.

On arrival, he was hemodynamically stable and afebrile at 37.9°C. His physical exam was notable for **point tenderness over the L2–L3 spinous processes** but no weakness, sensory loss, or sphincter disturbance. He could ambulate with discomfort.

Initial labs showed a WBC count of 10.8 x10⁹/L, **elevated CRP at 92 mg/L**, and ESR of 88 mm/h. Lumbar spine x-ray revealed only mild degenerative changes, but **MRI with contrast** showed disc space narrowing and vertebral enhancement at L2–L3, along with subtle paraspinal changes.

While awaiting blood culture results, the admitting team asked whether this was enough to confirm vertebral osteomyelitis—and what else might be needed to establish a definitive diagnosis.

### **Question 1**

Which of the following best supports clinical **suspicion for native vertebral osteomyelitis (NVO)?**

a) Fever, leukocytosis, and low back pain
 b) New or worsening back pain with elevated inflammatory markers
 c) Back pain with positive urinalysis and fever
 d) Radiographic evidence of disc degeneration, elevated D-dimer, and fever

✅ **Correct answer:** b
📚 *2015 IDSA NVO: RECOMMENDATIONS FOR CLINICAL DIAGNOSTICS, question 1*



**Culture Status:**

Blood cultures remain negative at 48 hours. MRI findings are strongly suggestive of discitis-osteomyelitis. The medical team asks about the next step to confirm the diagnosis.

### **Question 2**

What is the most appropriate next step to **establish a microbiologic diagnosis**?

a) Perform PCR on a blood sample
 b) Proceed with CT-guided aspiration biopsy
 c) Repeat blood cultures
 d) Test for latent tuberculosis

✅ **Correct answer:** b
📚 *2015 IDSA NVO: RECOMMENDATIONS FOR CLINICAL DIAGNOSTICS, question 3*



**Biopsy Results:**

CT-guided biopsy grows **Corynebacterium species**. The lab flags it as a likely contaminant. The patient remains febrile. Blood cultures remain negative.

### **Question 3**

How should this result be managed?

a) Start empiric broad-spectrum antibiotics
 b) Proceed with surgical debridement
 c) Repeat image-guided aspiration biopsy
 d) Start vancomycin and monitor for response

✅ **Correct answer:** c
📚 *2015 IDSA NVO: RECOMMENDATIONS FOR CLINICAL DIAGNOSTICS, question 6*



**Second Biopsy:**

Repeat CT-guided biopsy grows **anaerobic Gram-positive rods**, identified as **Cutibacterium acnes**. The patient is started on **ceftriaxone 2g IV daily**. After 10 days, his symptoms and inflammatory markers improve.

### **Question 4**

In suspected NVO with **skin flora growth**, which organism **does not** by itself require a repeat aspiration?

a) Coagulase-negative staphylococci (including *S. lugdunensis*) and Propionibacterium
 b) Propionibacterium and Brucella
 c) Mycobacteria and fungi
 d) Propionibacterium and diphtheroids

✅ **Correct answer:** d
📚 *2015 IDSA NVO: RECOMMENDATIONS FOR CLINICAL DIAGNOSTICS, question 6*

### **Follow-Up:**

The orthopedic team suggests a **follow-up MRI at 4 weeks** to assess early response. The patient is improving clinically.

### **Question 5**

What is the recommended role of **MRI during treatment** for NVO?

a) Repeat MRI at 4 weeks is routine to document response
 b) Only obtain MRI if CRP remains elevated
 c) Imaging is not routinely recommended if clinical response is evident
 d) MRI should always be repeated for medico-legal reasons

✅ **Correct answer:** c
📚 *2015 IDSA NVO: RECOMMENDATIONS FOR CLINICAL FOLLOW-UP, question 1*

**CLINICAL CASE 3: Vertebral osteomyelitis (VO)**

**Literature:**

1. 2015 Infectious Diseases Society of America (IDSA) Clinical Practice Guidelines for the Diagnosis and Treatment of Native Vertebral Osteomyelitis in Adults (2015 IDSA NVO)
2. A Guide to Utilization of the Microbiology Laboratory for Diagnosis of Infectious Diseases: 2018 Update by the Infectious Diseases Society of America and the American Society for Microbiology (2018 IDSA micro)
3. ACR Appropriateness Criteria Suspected Spine Infection (ACR spine infection)

**Level of consensus:**

1. Strong recommendation (> 90%)
2. The guideline does not provide level of evidence
3. Usually appropriate (7-9 points) and May be appropriate (4-6 points)

**Prompt:**

**You are acting as a board-certified infectious disease consultant.**
 Your role is to evaluate clinical scenarios presented to you by a practicing physician who is exploring how large language models like you can support decision-making in infectious disease care.

You will be given:

- A clinical case (including background, patient data, and key findings)
- A multiple-choice question with four options (a–d), only **one of which is correct**

Your task is to:

1. **Identify the single best answer** based on the clinical scenario.
2. **Justify your choice** using expert-level clinical reasoning, as if explaining your thought process to a fellow physician.
3. Keep your explanation under **500 words**, and avoid repeating the question or answer choices.
4. If evidence is unclear, acknowledge uncertainty and explain your reasoning as you would in a real consult.

Please format your response like this:

makefile

CopyEdit

Answer: [a / b / c / d]

Justification:
[Your clinical reasoning – max 500 words]



**Consult Request:**
 A 66-year-old man presents with acute neurologic symptoms and thoracic back pain. MRI shows an epidural phlegmon compressing the spinal cord. The ID team is consulted for diagnostic guidance and treatment strategy.

### **Clinical Summary:**

Mr. Michael Ramirez, a 66-year-old man with known coronary artery disease and chronic kidney disease, came to the hospital with **five days of progressive mid-thoracic pain**. On the day of presentation, he had begun noticing weakness in his left leg that made it difficult to walk.

He denied any recent trauma, procedures, or systemic infections. His vitals were notable for a low-grade fever (38.2°C) but otherwise stable parameters. On physical exam, he had **localized tenderness over the T7–T8 region**, weakness in the left lower limb (4/5 strength), and **hyperreflexia**, without sensory deficits.

Initial workup revealed a **WBC of 13.5 x10⁹/L**, **CRP of 106 mg/L**, and **ESR of 88 mm/h**. MRI with contrast demonstrated **destructive changes at T7–T8**, with a notable **epidural phlegmon compressing the cord**, and early disc space narrowing. Blood cultures were pending.

### **Question 1**

What is the most appropriate **next step** in management?

a) Start empiric antibiotics and await blood cultures
b) Start empiric antibiotics and consult surgery immediately
c) Defer antibiotics and arrange aspiration biopsy, as patient is stable
d) Repeat MRI in 48h to assess for radiologic progression

✅ **Correct answer:** b
📚 *2015 IDSA NVO: RECOMMENDATIONS FOR CLINICAL DIAGNOSTICS, question 4*



### **Clinical Course:**

The patient undergoes **urgent surgical decompression**. Cultures from surgical tissue grow **MSSA**. Blood cultures confirm the same organism. Histopathology is consistent with osteomyelitis. He improves postoperatively.

### **Question 2**

Is a **repeat biopsy** required in this setting?

a) Yes, to verify susceptibilities
b) Yes, if inflammatory markers persist
c) No, the microbiologic diagnosis is already established
d) No, unless bacteremia resolves before 72 hours

✅ **Correct answer**: c
📚 *2015 IDSA NVO: RECOMMENDATIONS FOR CLINICAL DIAGNOSTICS, question 3*

📚 2018 IDSA micro



**Therapy Initiated:**

IV **cefazolin** is started. After 2 weeks, the patient is afebrile, neurologically improved, and mobilizing with PT. Labs are trending down.

### **Question 3**

What is the guideline-based recommendation for **route of therapy**?

a) Always complete 6 weeks of IV therapy
b) At least 4 weeks IV, then oral if stable
c) Entire 6-week course may be oral if using bioavailable agents
d) Initial oral therapy should be avoided in all NVO cases

✅ **Correct answer:** c
📚 *2015 IDSA NVO: RECOMMENDATIONS FOR CLINICAL THERAPY, question 1*

### **Clinical Setback:**

At week 4, the patient develops new pain and low-grade fever. CRP rises from 12 to 78. Repeat MRI shows **progression of paraspinal phlegmon and new abscess**. Neurosurgery is reconsulted. A repeat sample is obtained.

### **Question 4**

When should samples be sent for **histopathology**?

a) Only if cultures are negative
b) Always, if adequate tissue can be safely obtained
c) Only in immunocompromised hosts
d) Only if unusual organisms (fungi, TB) are suspected

✅ **Correct answer:** b
📚 *2015 IDSA NVO: RECOMMENDATIONS FOR CLINICAL DIAGNOSTICS, question 5*

### **Question 5**

When performing imaging-guided aspiration, what should be included in the **microbiology workup**?

a) Aerobic cultures and histology only
b) Gram stain + aerobic culture; anaerobic optional
c) Gram stain, aerobic + anaerobic culture, and histopathology
d) No culture if antibiotics were given in the last 24 hours

✅ **Correct answer:** c
📚 2018 IDSA micro

**CLINICAL CASE 4: Vertebral osteomyelitis (VO)**

**Literature:**

1. 2015 Infectious Diseases Society of America (IDSA) Clinical Practice Guidelines for the Diagnosis and Treatment of Native Vertebral Osteomyelitis in Adults (2015 IDSA NVO)
2. A Guide to Utilization of the Microbiology Laboratory for Diagnosis of Infectious Diseases: 2018 Update by the Infectious Diseases Society of America and the American Society for Microbiology (2018 IDSA micro)
3. ACR Appropriateness Criteria Suspected Spine Infection (ACR spine infection)

**Level of consensus:**

1. Strong recommendation (> 90%)
2. The guideline does not provide level of evidence
3. Usually appropriate (7-9 points) and May be appropriate (4-6 points)

**Prompt:**

**You are acting as a board-certified infectious disease consultant.**
 Your role is to evaluate clinical scenarios presented to you by a practicing physician who is exploring how large language models like you can support decision-making in infectious disease care.

You will be given:

- A clinical case (including background, patient data, and key findings)
- A multiple-choice question with four options (a–d), only **one of which is correct**

Your task is to:

1. **Identify the single best answer** based on the clinical scenario.
2. **Justify your choice** using expert-level clinical reasoning, as if explaining your thought process to a fellow physician.
3. Keep your explanation under **500 words**, and avoid repeating the question or answer choices.
4. If evidence is unclear, acknowledge uncertainty and explain your reasoning as you would in a real consult.

Please format your response like this:

makefile

CopyEdit

Answer: [a / b / c / d]

Justification:
[Your clinical reasoning – max 500 words]

**Clinical presentation**

A 58-year-old man with diabetes and known bicuspid aortic valve is admitted with fever and new-onset lumbar pain. The primary team has asked ID to assist with evaluation for possible native vertebral osteomyelitis and to coordinate further diagnostic workup.

### **Clinical Course:**

Mr. Charles Daniels was in his usual state of health until approximately ten days ago, when he developed dull lower back pain that gradually intensified. Initially he attributed it to overuse, but over the last 48 hours he began experiencing fatigue, intermittent chills, and one documented fever of 38.5°C. He denied trauma, urinary symptoms, or leg weakness, but noted that walking had become increasingly uncomfortable.

He has a history of well-controlled type 2 diabetes, a bicuspid aortic valve diagnosed in his 40s, and long-standing psoriasis, for which he has occasionally used topical steroids and methotrexate.

On admission, he was hemodynamically stable and alert. He appeared uncomfortable while sitting or shifting in bed. There was marked tenderness to percussion at the L3–L4 level, but his neurologic exam was nonfocal: full strength and sensation in all extremities, and no urinary retention.

Laboratory evaluation revealed an elevated CRP of 116 mg/L and ESR of 96 mm/h. White blood cell count was 11.3 x10⁹/L. Two sets of blood cultures were obtained before empiric antibiotics and subsequently flagged positive for *Streptococcus sanguinis*.

### **Imaging and Cardiac Workup:**

MRI of the lumbar spine showed discitis-osteomyelitis involving the L3–L4 intervertebral space with adjacent vertebral body enhancement and no evidence of epidural abscess. Given the bloodstream infection and known valvular disease, a transthoracic echocardiogram was also performed and showed a mobile echodensity on the aortic valve, raising concern for early endocarditis.

### **Question 1**

Which aspect of this clinical picture most strongly supports a diagnosis of vertebral osteomyelitis?

a) New-onset low back pain and urinary tract infection
b) Back pain in a patient with bloodstream infection or suspected endocarditis
c) Chronic sciatica in the setting of lumbar disc degeneration
d) Mid-back pain in a patient with rheumatoid arthritis flare

✅ **Correct answer:** b
📚 *RECOMMENDATIONS FOR CLINICAL DIAGNOSTICS, question 1*

During morning rounds, your resident asks whether a full neurologic exam is really necessary in patients like this, given that he has no reported weakness or paresthesias.

### **Question 2**

What is the appropriate response?

a) Perform only motor testing
b) Focus on spinal percussion for localization
c) Full motor and sensory exam is required
d) Neurologic exam is unnecessary unless focal signs are reported

✅ **Correct answer:** c
*📚 RECOMMENDATIONS FOR CLINICAL DIAGNOSTICS, question 2*

You review the labs and MRI, which support the diagnosis, and the question now arises: was this diagnostic workup sufficient? Or should something more have been done initially?

### **Question 3**

Which workup approach is considered appropriate in this case?

a) Blood cultures only
b) MRI and blood cultures
c) ESR, CRP, and two sets of blood cultures
d) Plain lumbar X-ray and CRP

✅ **Correct answer:** c
📚 *RECOMMENDATIONS FOR CLINICAL DIAGNOSTICS, question 1*

After 5 days of IV penicillin, the patient remains intermittently febrile. A CT-guided aspiration biopsy is attempted but fails to yield a positive culture. Histology is inconclusive.

### **Question 4**

How should this be approached?

a) Stop antibiotics for 72 hours, repeat blood cultures, and restart therapy
b) Consider viral etiology and taper antimicrobials
c) Test for difficult-to-grow organisms (e.g., mycobacteria, fungi, Brucella)
d) Discontinue antimicrobials and reassess in 1 week

✅ **Correct answer:** c
📚 *RECOMMENDATIONS FOR CLINICAL DIAGNOSTICS, question 6*

A Brucella serology is sent based on epidemiologic history (he had frequent travel to Spain for business in past years) and returns positive for both IgM and IgG. Antibiotics are transitioned to **doxycycline and rifampin**.

### **Question 5**

What is the recommended total **treatment duration** for Brucella-related vertebral osteomyelitis?

a) 4 weeks
b) 6 weeks
c) 8 weeks
d) 12 weeks

✅ **Correct answer:** d
📚 *RECOMMENDATIONS FOR CLINICAL THERAPY, question 1*
